# The *BUD31* Homologous Gene in *Schizosaccharomyces pombe* Is Evolutionarily Conserved and Can Be Linked to Cellular Processes Regulated by the TOR Pathway

**DOI:** 10.3390/cells14211736

**Published:** 2025-11-05

**Authors:** Ildikó Vig, Lajos Acs-Szabo, Zsigmond Benkő, Silvia Bagelova Polakova, László Attila Papp, Juraj Gregan, Ida Miklós

**Affiliations:** 1 Department of Genetics and Applied Microbiology, Faculty of Science and Technology, Institute of Biotechnology, University of Debrecen, Egyetem tér 1, H-4032 Debrecen, Hungary; vig.ildiko@science.unideb.hu (I.V.); acs-szabo.lajos@science.unideb.hu (L.A.-S.); papp.laszlo.attila@science.unideb.hu (L.A.P.); 2 Department of Molecular Biotechnology and Microbiology, Faculty of Science and Technology, Institute of Biotechnology, University of Debrecen, Egyetem tér 1, H-4032 Debrecen, Hungary; benko.zsigmond@science.unideb.hu; 3 Department of Botany, Faculty of Science and Technology, Institute of Biology and Ecology, University of Debrecen, Egyetem tér 1, H-4032 Debrecen, Hungary; 4 Department of Chromosome Biology, University of Vienna, Vienna Biocenter (VBC), Dr. Bohr-Gasse 9, 1030 Vienna, Austria; silvia.bagelova@savba.sk (S.B.P.); juraj.gregan@univie.ac.at (J.G.); 5 Institute of Animal Biochemistry and Genetics, Centre of Biosciences, Slovak Academy of Sciences, 840 05 Bratislava, Slovakia; 6 Institute of Microbial Genetics, Department of Agricultural Sciences, BOKU University Vienna, Campus Tulln, Konrad Lorenz Strasse 24, 3430 Tulln an der Donau, Austria

**Keywords:** *BUD31* gene, splicing, TOR-pathway, *Schizosaccharomyces pombe*, interspecific complementation, cell division

## Abstract

The human *BUD31* gene has been associated with various processes including cancer. To better understand its function, we used genetic methods to study *Schizosaccharomyces pombe* cells lacking the *BUD31* homologous gene (*cwf14*) and performed sequence analysis using bioinformatics methods. Mutant cells lacking the *cwf14* gene showed cell size and division defects, altered stress response, rapamycin sensitivity, enhanced chronological aging, and increased sporulation tendency. These processes are known to be regulated by the TOR pathway. The *cwf14*-TOR link was also supported by further experiments. We demonstrated that most protein-coding genes affected by *cwf14* deletion are upregulated, encode hydrolases, oxidoreductases, and are often involved in transport. GO enrichment drew our attention to genes related to nitrogen transport, while additional data pointed to a nutrient/nitrogen (N) sensing problem. Although Cwf14 protein is associated with spliceosome complex, most genes affected by the absence of *cwf14* do not contain introns, suggesting that they are influenced indirectly by the *cwf14* gene. In silico experiments have revealed that *BUD31* orthologous genes are found from yeast to humans, are evolutionarily conserved with a high degree of sequence identity, conserved motifs, and structures. Since the human gene partially complemented the mutant phenotype of *S. pombe* cells, indicating functional homology, our data can help better understand pathological mechanisms observed in human cancer cells.

## 1. Introduction

The human BUD31 protein proved to be an important co-activator of the androgen receptor’s transcriptional activity [1], while other studies revealed that deletion or overexpression of this gene has been associated with various types of cancers [2,3,4,5,6]. In addition, high *BUD31* expression correlated with worse survival outcomes [6]. Knockout of mice *Bud31*, the homolog of the human *BUD31* gene (*hBUD31*), led to loss of spermatogonia and male infertility [7]. Further studies on model organisms revealed additional phenotypic changes in the absence of this gene. For example, the *Saccharomyces cerevisiae bud31* mutant strain showed abnormal budding patterns, altered cell shape and bud morphology, disorganized actin cytoskeleton, and decreased cell growth at non-optimal temperatures [8,9]. The mutant cells showed increased duration in the G1 phase of the cell cycle [10], while others revealed that this gene is involved in stress tolerance [11]. The mutant strain had decreased metal resistance [12], and endocytosis [13]. *Candida* mutant cells containing a mutation in the homologous gene (*cBUD31*) were altered in size [14], while the *S. pombe* cells carrying a mutation in the *BUD31* homologous gene (*cwf14*; Complexed With cdc Five) exhibited cell wall defects, abnormal morphology at 32 °C, and decreased cell growth on media with glucose as a carbon source [15,16]. The normal function of the *BUD31* homologous gene in *S. pombe* was also necessary for the pericentric heterochromatin integrity and assembly [17,18].

Analysis of human and yeast spliceosome complexes, which contain numerous proteins and are necessary for removing noncoding introns from precursor mRNAs [19,20], revealed that the *S. pombe* Cwf14 and its counterpart proteins belong to this complex [21,22,23]. This relation was supported by further results, for example, that *S. cerevisiae* Bud31 and the homologous *S. pombe* Cwf14 proteins were identified as splicing factors [9,17,22,24], or that *Arabidopsis* BUD31 protein can interact with transcriptional elongation proteins [25].

Although the above data can suggest that the various phenotypic changes found in mutant cells may be the result of altered splicing, the exact details of the changes caused by mutations in *BUD31* genes are unclear. Since *S. pombe* is an attractive model organism for studying human homologous genes [26], and since our preliminary data suggested that the *cwf14* mutant strain exhibited a multiseptated phenotype under certain conditions, similar to cell separation (sep) mutants isolated in our laboratory [27,28,29], we used this model organism to obtain further data on the function of the *cwf14* gene. We wanted to explore the relationship between the *cwf14* gene and cell division, and since its homologous genes can be found in both yeast and human cells, we also wanted to gain more information about its evolutionary conservation. We investigated the *cwf14* gene with in silico and molecular approaches.

## 2. Materials and Methods

### 2.1. Strains

Yeast strains used in this study are listed in Table S1. The *cwf14*Δ*::kanMX6 ade6-M216 ura4-D18 leu1-32 h^+^* (2-1480) mutant strain was purchased from the Bioneer Company (Daejeon, Republic of Korea) (M-3030H *S. pombe* Haploid Deletion Mutant Set ver2.0/3.0 in ED666 *h^+^ ade6-M210 ura4-D18 leu1-32*, or ED668 *h^+^ ade6-M216 ura4-D18 leu1-32*). The *cwf14*Δ*::kanMX6* is in ED668 *h^+^* strain, and its position code is V3-P08-12. The strain was G418 resistant, had adenine, uracil, and leucine auxothropic mutations. The Pombase code of *cwf14* is SPBC24C6.11 [30,31].

Since the 2-1480 strain contained many auxotrophic mutations, and we wanted to decrease their number, this strain was backcrossed with wild-type h^−^ (0-1) and *leu1-32 h^−^* (2-1199) strains, and G418 resistant (*cwf14*Δ*::kanMX6*) (2-1542) or G418 resistant plus leucine auxotrophic spore clones (*cwf14*Δ*::kanMX6 leu1-32*) (2-1530, 2-1532) were isolated (Table S1). These strains were used for phenotypic characterization. The *S. pombe* wild-type (0-1, 2-1201, 0-3) and *leu1-32 h^−^* (2-1199) strains were used as controls in the experiments (Table S1).

Strains used for sporulation tests: The 15831 strain was created by crossing the PP574 [32] and P138 [33] strains. Transformation of the 15831 strain, to introduce a CloneNat marker, resulted in the 15884 (B3 *h^+^* CEN1b). The 15953 (B3 *h^90^* CEN1b) strain is a spontaneous *h^90^* mutant of the strain 15884. Strain 16051 originated from strain 15953, after the insertion of h2a-mCherry-Hyg1 into the intergenic region between *snoz30* and *rpl8* close to *his7*.

For the preparation of strains suitable for studying homozygous meiotic chromosome segregation (homozygous for CEN1b), we crossed the *cwf14*Δ*::kanMX6 ade6-M216 ura4-D18 leu1-32 h^+^* (2-1480) and 16051 (B3 *h^90^* CEN1b h2a-mCherry) strains. The resulting *cwf14*Δ*::kanMX6 ade6-M216 ura4-D18 leu1-32* CEN1b h2a-mCherry *h^90^* spores were used for studying homozygous meiotic chromosome segregation.

For preparation of strain 2-1490 (SO9 *h^−^* CEN1b) suitable for studying heterozygous meiotic chromosome segregation (heterozygous for CEN1b), we crossed the *cwf14*Δ*::kanMX6 ade6-M216 ura4-D18 leu1-32 h^+^* (2-1480) and 16903 (B3 *h^−^* CEN1b) strains (Table S1).

For long-term preservation of plasmids, the DH5α *E. coli* strain was used.

### 2.2. Media

Generally, YEA (Yeast Extract Agar) (1% yeast extract, 2% glucose, 2% agar), YEL (YEA without agar), or YPG (Yeast Peptone Glucose) (1% yeast extract, 2% peptone, 2% glucose) media were used for culturing.

For backcrossing of the *cwf14*Δ*::kanMX6 ade6-M216 ura4-D18 leu1-32 h^+^* (2-1480) strain, a medium suitable for sporulation (SPAS) supplemented with 7.5 mM adenine, leucine, and uracil was used [34].

YEA + 400 µg/mL G418 medium (Sigma-Aldrich, St. Louis, MO, USA) was used for the selection of *cwf14*Δ::*kanMX6* strains, while leucine auxotrophy was tested on EMMA (Edinburgh Minimal Medium Agar) [35] and EMMA supplemented with 7.5 mM leucine.

The transformed *S. pombe* cells were grown on Synthetic Minimal Agar (SMA) and EMMA and supplemented with 15 μM thiamine (*nmt^+^* promoter of the vector repressed) [34,35,36]. Later, the transformants were investigated on SMA (*nmt^+^* promoter of the vector induced).

For spot assays, SMA + 12 mM caffeine, SMA + 5%, and 8% ethanol, or SMA + 100 ng/mL rapamycin (Sigma) were used.

*E. coli* cells were cultured on LB medium or after transformation with pREP vectors on LB + 50 mg/mL ampicillin (Sigma) [34].

The following culture media were used to examine the chromosome segregation and strain selection: YES (0.5% yeast extract, 3% glucose, 2% agar, with adenine (0.125 g/L), leucine (0.1 g/L), uracil (0.1 g/L), and histidine (0.1 g/L)) supplemented with G418 (150 mg/L) or Hygromycin (200 mg/L) or Nourseothricin (100 mg/L) if required; EMM supplemented with adenine (0.125 g/L), leucine (0.1 g/L), and histidine (0.1 g/L); PMG-N that is EMM without nitrogen containing only 10 g/L glucose and supplemented with leucine (0.1 g/L), histidine (0.1 g/L), uracil (0.1 g/L) and adenine (0.125 g/L).

### 2.3. Preparation of cwf14Δ::kanMX6 CEN1b GFP (Green Fluorescent Protein) Labeled Strains

The Bioneer KO (*cwf14* disrupted) (2-1480) *h^+^* strain was mixed with B3 *h^90^* (16051) cell suspension (prepared in sterile water). The cells of the mixed culture were spread on PMG-N + ade + leu + his + ura sporulation medium and incubated for 2 days at 25 °C. Afterward, to remove the vegetative cells, the agar plates were incubated at 42 °C for three days (the spores survived this temperature). The spores were first transferred with replica plating onto the surface of a nutrient-rich medium supplemented with geneticin (YES + G418) to allow germination of spores containing *kanMX6* deletion cassette (3–5 days at 32 °C). The resulting cells were further transferred to hygromycin-supplemented rich medium (YES + hyg) to select mCherry-labeled h2a cassette (2 days at 32 °C). In the third step, the cells were transferred to nourseothricin-supplemented rich medium (YES + nourseothricin) to select for lacI-GFP cassette (2 days at 32 °C). In the fourth step, the cells were transferred to minimal media lacking uracil (EMM-ura) to select for the lacOp cassette containing chromosome 1 (1 day at 32 °C). After this selection series, the cells containing the *cwf14*Δ*::kanMX6* deletion mutant (G418), the mCherry tagged h2a histone close to his7 locus (hygromycin), the GFP tagged LacI gene in the *his7* locus (nourseothricin) and several copies of the lacOp cassette in the dh1L locus of CEN1b region (no uracil) survived. The mating type of selected cells will be mostly *h^90^*. These cells were transferred onto the surface of a sporulation medium (PMG-N) to allow mating and sporulation for 20 h at 25 °C. The chromosome segregation in the asci was examined under a fluorescence microscope using GFP and Rhodamin filters (to visualize GFP-labeled chromosomal DNA and mCherry-tagged- histone h2a, respectively).

### 2.4. Study of Sporulation and Meiotic Chromosome Segregation

For checking conjugation and meiosis, we crossed the *cwf14*Δ*::kanMX6 leu1-32 h^−^* cells (2-1532) with the wild-type *h^+^* strain (2-1201) on SPAS + leucine [34], and the Petri dishes were incubated at 30 °C. After 1 day, asci were photographed.

To test sporulation frequency, the *cwf14*Δ*::kanMX6 leu1-32 h^90^* (2-1530) and wild-type *h^90^* (0-3) strains were streaked on YEA and EMMA-N. The agar plates were incubated at 30 °C, and sporulation was examined after 1 and 2 days under a microscope (Olympus BH2).

Meiotic chromosome segregation was investigated in 346 asci obtained from the cross of homothallic strains where chromosome 1 was labeled with GFP (homozygous CEN1b) (2-1480 *h^+^* x 16051 *h^90^*) and in 688 asci having heterozygous CEN1b (2-1490 *h^−^* x 2-1480 *h^+^*), while 52 zygotes were also immunostained (2-1490 *h^−^* x 2-1480 *h^+^*).

Immunostaining, to analyze chromosome segregation in *S. pombe* cells, was performed as previously described [37]. Slides were prepared using poly-L-lysine coated cover slip and Vectashield Mounting Medium for Fluorescence [37]. Asci with four GFP dots were considered wild-type asci, while asci with fewer than four GFP dots (indicating the absence of GFP labeled chromosome 1 in spores) were considered missegregated. The position of the GFP dots in asci was also taken into account, and the following categories (X- -X, -XX-, X-X-) were scored (“X” indicates spore containing GFP dot, while “-” indicates a spore with no GFP dot).

### 2.5. PCR Test to Prove Disruption of the cwf14 Gene

The presence of the *cwf14* disrupted allele was checked in the G418-resistant spore clones by the colony PCR method [38]. For PCR reaction Dream Taq DNA Polymerase (Thermo Fisher Scientific, Waltham, MA, USA), *cwf14* specific (906-907), or *kanMX6* cassette and *cwf14* specific (527-907) primers (Table S2), and the following parameters were used: 95 °C 2 min, 95 °C 30 s, 64 °C 1 min, 72 °C 2 min (25 cycles), 72 °C 10 min, 4 °C ∞. PCR products were investigated by agarose gel electrophoresis (1 × TBE, 1% agarose gel, 120 V).

### 2.6. PCR Amplification of the BUD31 Genes

For the amplification of *S. pombe cwf14* and *cBUD31* genes, genomic DNAs were used as a template. They were isolated from *S. pombe* L972 strain and obtained from Prof. Tamás Emri (*C. albicans* SC5314 strains). For *hBUD31*, cDNA was used as a template (obtained from Dr Erika Zilahi, University of Debrecen). The primers used are listed in Table S2. The cycling parameters were 98 °C 2 min, 98 °C 30 s, 64 °C 1 min (*S. pombe cwf14* gene), 72 °C 2 min, 4 °C ∞. We used the same parameters for amplifying the *cBUD31* and *hBUD31* genes, but the annealing temperature was 61.5 °C for the *cBUD31* gene and 65 °C for the *hBUD31* gene, instead of 64 °C.

Genomic DNA and total RNA were isolated using the protocols described in [39,40]. RNA was purified with Qiagen RNeasy mini spin columns, while the cDNA was transcribed by M-MLV reverse transcriptase (Promega, Madison, WI, USA) with oligo-dT primers according to the manufacturer’s instructions. The reverse transcription of the human RNA was carried out with the following parameters: 42 °C 15 min, 95 °C 5 min, 4 °C 5 min.

### 2.7. Cloning of the BUD31 Orthologous Genes

*BUD31* PCR products were cloned into the pJET1.2 cloning vector (Thermo Scientific™, K1231, Waltham, MA, USA) according to the manufacturer’s instructions and later into the XhoI-SmaI sites of the pREP vectors [41]. pREP vectors have inducible promoters (*nmt1^+^*), which can be regulated by thiamine. The gene cloning was checked by Sanger sequencing, and the vectors containing proper *cwf14* and *BUD31* genes were transformed into chemically competent *E. coli* DH5α cells (collection numbers: 575, 735, 737) (Table S3). The bacterial cells were transformed by the standard heat-shock method [39].

### 2.8. Transformation of the Yeast Cells

The pREP vector + *cwf14* or *cBUD31* or *hBUD31* constructions were introduced into *cwf14*Δ*::kanMX6 leu-32* (2-1530) strains. The *S. pombe* cells were transformed by electroporation (Bio-Rad Xcell Pulser, Hercules, CA, USA) using the manufacturer’s instructions based on Prentis [42]. After transformation, the cells were grown on minimal medium EMMA supplemented with thiamine (*nmt1^+^* promoter repressed) at 25 °C. Later, the transformant colonies were isolated, and they were tested with the plasmid loss test.

### 2.9. Stress Response Test

The cells were cultured in YEL to an OD_590_: 0.2 cell density. 10-fold dilution series (10×, 100×, 1000×) were prepared, and 5 µL was spotted from the dilutions onto the surface of the various media (SMA, SMA + 10 and 12 mM caffeine, SMA + 5% and 8% ethanol, SMA or YEA + 100 ng/mL rapamycin). The Petri dishes were incubated for 4 days at 30 °C, or in the case of temperature-sensitivity tests at 18 °C, 25 °C, and 37 °C. The experiments were repeated three times.

### 2.10. Investigation of Cell Morphology

The morphology of 100 cells grown on 12 mM caffeine-containing YEA (1 day, at 30 °C) was investigated under a microscope (Olympus BH2, Olympus Global, Tokyo, Japan).

### 2.11. Cell Length

The cells were incubated on SMA at 30 and 37 °C, for 24 h. The length of 100 cells was measured under a microscope (Olympus BH2). The data was subjected to statistical analysis.

### 2.12. Long-Term Survival Assay

To investigate the response to starvation, a long-term survival assay was performed. Cells were streaked onto the surface of the YPG medium, and they were incubated for 4 weeks at room temperature. A cell suspension was prepared with Milli-Q water (MQ), and an equal amount of the cells was spread onto YPG. The Petri dishes were incubated at 30 °C, and the number of colonies was counted after 4 days.

### 2.13. Growth in Complex Media

The *cwf14*Δ*::kanMX6*, *leu1-32* (2-1532) strain was inoculated into 20 mL YPL and incubated at 30 °C in a shaker. To avoid the possible effect of the *leu1* mutation, *leu1-32* (2-1199) was used as a control. Their cell density (OD_595_) was measured every 2 h. The results are the mean values of three separate experiments. Other *S. pombe* techniques were described in [43,44,45].

### 2.14. Bioinformatics Analyses

#### 2.14.1. Sequence Retrieval and Motif Analyses

*S. pombe cwf14* DNA and Cwf14 protein sequences were downloaded from Pombase (SPBC24C6.11) (https://www.pombase.org) (accessed on 14 June 2024) [30]. Motif search for the Cwf14 was performed with the InterProScan (release 100.0) (https://www.ebi.ac.uk/interpro/search/sequence-search, accessed on 30 October 2025) and with the ScanProsite tool (https://prosite.expasy.org/scanprosite, accessed on 30 October 2025). Signature motifs of the BUD31 protein were extracted from the PRINTS database (http://130.88.97.239/PRINTS/index.php, accessed on 30 October 2025) using the FPScan tool.

#### 2.14.2. Orthology Inference and Comparative Sequence Analyses

Putative orthologues were identified by BLASTp at the website of NCBI (v2.15.0) (https://blast.ncbi.nlm.nih.gov/Blast.cgi?PAGE=Proteins) (accessed on 10 June 2024) and by HMMER search at EMBL-EBI (https://www.ebi.ac.uk/Tools/hmmer/search/phmmer, accessed on 30 October 2025) using the Cwf14 protein sequence of *S. pombe* as a query with default parameters in both cases. Additional orthologous sequences were extracted from the Pfam protein profile database (https://pfam.xfam.org, accessed on 30 October 2025) (v37.0) and UniProt (release 2020_01) (https://www.uniprot.org) (accessed on 11 June 2024). The found sequences were validated by reciprocal BLASTp searches in the concerned *S. pombe* database (https://fungi.ensembl.org/Schizosaccharomyces_pombe/Tools/Blast?db=core, accessed on 30 October 2025). 

Global distribution of putative orthologues of the Cwf14 protein sequence was estimated with BLAST-EXPLORER using the non-redundant protein database of NCBI [46]. Comparative sequence analyses of the proteins were conducted by aligning them with Clustal Omega (https://www.ebi.ac.uk/Tools/msa/clustalo, accessed on 30 October 2025) and with MUSCLE v3.8.31 [47] (http://www.phylogeny.fr/one_task.cgi?task_type=muscle, accessed on 30 October 2025). To perform pairwise alignments, a Needleman–Wunsch algorithm at the website of EMBL-EBI (http://www.ebi.ac.uk/Tools/psa/emboss_needle/nucleotide.html, accessed on 30 October 2025) or the NCBI BLAST2p (http://blast.ncbi.nlm.nih.gov, accessed on 30 October 2025) were used. 

Sequence logos were generated from multiple alignments with the tool provided at the Weblogo server (http://weblogo.berkeley.edu/logo.cgi, accessed on 30 October 2025) [48].

#### 2.14.3. Protein Structure Analyses

To compare certain Cwf14 (BUD31) orthologous protein sequences at the secondary and tertiary structure level, 3D models were built with the Phyre^2^ server (v2.1) (http://www.sbg.bio.ic.ac.uk/phyre2/html/page.cgi?id=index) [49] and SWISS-MODEL (https://swissmodel.expasy.org) using c2my1A, c3jb9e, and c5mqfQ as templates (accessed on 13 July 2024). The predicted structures were visualized with UCSF Chimera software (v1.13) (http://www.rbvi.ucsf.edu/chimera, accessed on 30 October 2025) [50].

#### 2.14.4. Phylogenetic Tree Construction

Phylogenetic trees were created at the websites of Phylogeny.fr (http://www.phylogeny.fr) [51] and ATGC (http://www.atgc-montpellier.fr/phyml) [52] (accessed on 20 July 2024). The chosen protein sequences were aligned with MUSCLE (full mode, maximum iteration: 16) [47], and the ambiguous regions were removed with GBLOCKS v0.91b [53] (http://www.phylogeny.fr/one_task.cgi?task_type=gblocks, accessed on 30 October 2025). For phylogenetic tree inference, two algorithms were used: MrBayes v3.2.6 (Bayesian inference) [54] and PhyML v3.0 (maximum likelihood) [52].

The substitution model was chosen with Smart Model Selection (SMS) [55]. One of the best models suggested by the Akaike Information Criterion (3989.02952) and the Bayesian Information Criterion (4093.66594) was WAG + G [56]. MrBayes v3.2.6 was used as follows: the WAG model was used for amino acid residue substitution, while the rate variation across sites was adjusted to gamma distributed. The distribution is approximated using four categories. Four MCMC chains (one cold and three heated) were run for 100.000 generations, trees were sampled every 10 generations, and the first 250 sampled trees were discarded as “burn-in”. The average standard deviation of split frequencies was 0.007937 at the end of the analysis, indicating that a convergence had occurred. The average PSRF (potential scale reduction factor) for parameter values was 1.001. A consensus tree was derived from a total of 15,002 trees.

For the PhyML analysis, the WAG substitution model was also chosen. The number of substitution rate category was adjusted to 4, gamma distribution parameter was estimated, and the proportions of invariable sites were fixed to 0. Branch support was estimated with the approximate likelihood ratio test (aLRT SH-like) [57]. The trees were displayed with FigTree v1.4.4 (http://tree.bio.ed.ac.uk/software/figtree, accessed on 30 October 2025).

#### 2.14.5. GO Enrichment

GO enrichment of the protein-coding genes affected by *cwf14* mutation identified by Kallgren [17] was performed with the ShinyGO 0.80 and later 0.82 program (http://bioinformatics.sdstate.edu/go) (accessed on 19 August 2024) [58], using default settings.

#### 2.14.6. Presence of Introns in Genes Affected by *cwf14* Mutation

To identify the presence of introns in the genes affected by *cwf14*, the protein-coding genes that had at least +/− higher than 1.5 log_2_ value were selected from the supplemental material of Kallgren [17]. The DNA sequences and the introns of these genes were obtained from the Pombe database (http://www.pombase.org) (accessed on 1 July 2023) [30].

#### 2.14.7. Statistical Analyses

Normal distribution was tested by the Shapiro–Wilk test, and in the case of normal distribution one-tailed t probes were used to assess the significant discrepancy between the samples. Otherwise, the Mann–Whitney U test was used. For datasets that were not normally distributed, the Kruskal–Wallis test was used for multiple comparisons followed by the Bonferroni corrected pairwise Dunn test as post hoc tests. *p* values were considered significant below the alpha level of 0.05. All statistical analyses were performed with the Past v4.09 program [59].

## 3. Results

### 3.1. Disruption of cwf14 Gene Caused a Pleiotropic Phenotype

**Figure 1:**
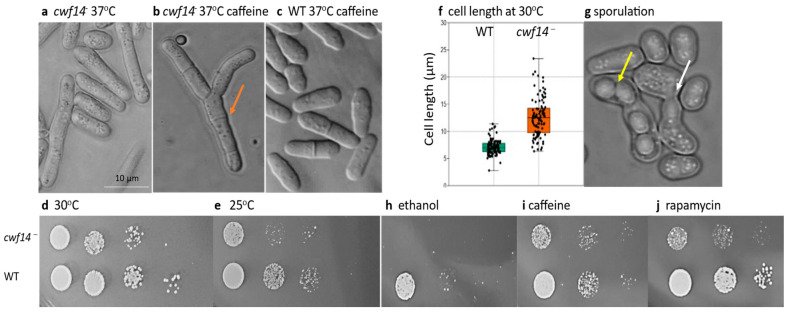
Phenotype of the mutant strain (*cwf14^−^*). Morphology: The mutant strain produced elongated cells at 37 °C (YEA) (2-1480) (**a**), and multiseptated morphology on caffeine-containing medium (YEA + 10 mM caffeine, 37 °C) (2-1480) (**b**), in contrast to control cells (WT: wild-type (0-1), which showed normal (unicellular) morphology (YEA + 10 mM caffeine, 37 °C) (**c**). The orange arrow shows one of the septa between the cells. Growth, cell size, and sporulation: The growth of cells (*cwf14^−^*: 2-1542; WT: 0-3) was tested at 30 °C (**d**) and 25 °C (SMA) (**e**), showing slower growth of mutant cells. The size of the cells was also measured at 30 °C (SMA) and was found to be significantly longer in the mutant than in the wild-type strain (**f**). The *cwf14^−^* cells (2-1530) were able to conjugate and sporulate (**g**) (SPAS + leucine; 30 °C). The yellow arrow shows ascus with four spores, and the white arrow shows conjugating cells. Stress response: Growth of the mutant strain was examined on media containing 8% ethanol (**h**), 12 mM caffeine (**i**), and 100 ng/mL rapamycin (**j**) (SMA, 30 °C, 4 days), and was compared to the control plates without additives (**d**). Cell density decreases from left to right because serial dilutions of *cwf14^−^* and WT cell suspensions were dropped on the culture medium.

Preliminary screening of the *S. pombe* mutant strains indicated that disruption of the *cwf14* gene (2-1480 strain) can lead to a slow-growth phenotype (S. Polakova, Z. Benko and J. Gregan, unpublished data) (Figure S1), longer cell size at 37 °C (Figure 1a), and production of multiseptated cells in the presence of caffeine (Figure 1b), compared to the unicellular wild-type cells (Figure 1c). Similar observations have been published in independent reports [15,60].

**Figure 2:**
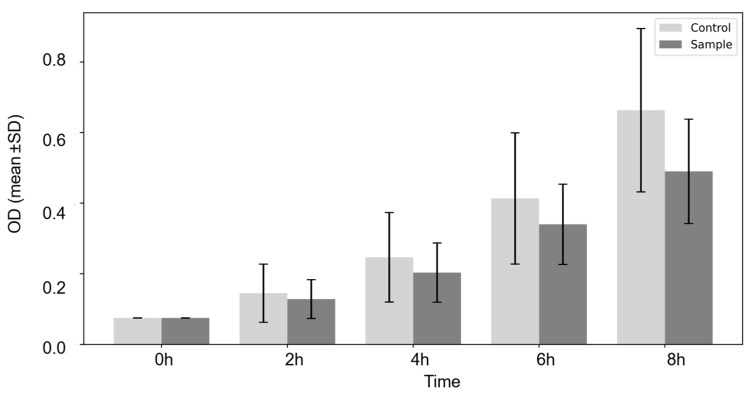
The growth of the *cwf14* mutant strain in liquid medium. Control: *leu1-32* (2-1199); sample: *cwf14*Δ*::kanMX6 leu1-32* (2-1532). The cells were cultured in YPL, in a shaker, at 30 °C. OD_595_ values were measured at inoculation (0 h), and 2, 4, 6, 8 h after inoculation. After 24 h, the difference between the strains was even greater: OD_595_ was 5.07 (control) and 4.30 (sample). The results are the mean values of three separate experiments.

Since the *cwf14* disrupted strain (2-1480) contained three auxotrophic mutations in its genome (Table S1), we wanted to eliminate their possible negative effects and clarify whether the phenotypic changes were indeed linked to the *cwf14* gene. Therefore, the mutant strain 2-1480 was backcrossed with wild-type and *leu1-32* strains (2-1199). G418-resistant spore clones were isolated with a leucine mutation (*cwf14*Δ*::kanMX6 leu1-32;* 2-1530, 2-1532), and without an auxotrophic marker (*cwf14*Δ*::kanMX6*) (2-1542) (Table S1), and their growth and morphology were also examined. Cell division of these strains was slower in both minimal (SMA), even at 30 °C (Figure 1d), and complex medium (YPL) (Figure 2). The difference in growth was more pronounced when the culture temperature was changed to 25 °C (Figure 1e) (similar results were obtained at 18 and 37 °C). The longer cell size of the mutant cells was confirmed at 37 °C, and this was also true at lower temperatures, not only in complex [15] but also in minimal medium (30 °C, SMA, pH 6.49, after 6 days) (Figure 1f). The multiseptated cell morphology of the *cwf14*Δ*::kanMX6* strain (2-1542) was also similar to that previously noticed on the caffeine-containing medium (Figure 1b).

Crossing *cwf14*Δ*::kanMX6 leu1-32* (2-1532) with the wild-type strain (2-1201) (SPAS + leucine) revealed that the *cwf14* mutant cells can conjugate and sporulate (Figure 1g). However, when the sporulation of a strain with chromosome 1 marked with GFP was examined, the meiotic chromosome segregation was not normal. In homozygous *cwf14*Δ*::kanMX6* strains carrying both copies of chromosome 1 sequences marked with GFP, only 89.6% of the asci contained GFP dots in all four spores (310 asci from 346), while 8.4% of asci contained three, and 1.9% of asci contained only two spores with GFP dots. In contrast, in strains carrying the wild-type allele of *cwf14*, 100% of the asci (66,992 asci) contained four spores with GFP dots. This meiotic chromosome missegregation phenotype was confirmed by the examination of a strain in which only one copy of chromosome 1 was marked by GFP (heterozygous GFP dots). In *cwf14::kanMX6* mutant strains, only 82.4% of asci (567 asci from 688) showed the expected phenotype (XX- -) (“X” indicates a spore containing GFP dot, while “-” indicates a spore with no GFP dot). 2.2% (15 asci) had only one spore with GFP dot (X- - -), while 15.5% (107 asci) contained two spores with GFP dots, but their positions in the asci were not proper (X-X-). As expected, control asci carrying wild-type allele of *cwf14* and heterozygous GFP dots (1110 asci) contained two spores with GFP dots (XX- -) in 97.57%. In addition, analysis of GFP dots in 52 asci by immunostaining revealed that missegregation of chromosomes in the *cwf14*Δ*::kanMX6* mutant occurred mainly in meiosis I.

Stress response assays also demonstrated that the *cwf14^−^* mutant strains (2-1532, 2-1480) are sensitive to rapamycin, ethanol, and caffeine (Figure 1h–j), confirming previous results [60,61].

### 3.2. The Protein-Coding Genes Affected by cwf14 Mutation Are Often Involved in Transport Processes, Encode Enzymes, and Rarely Contain Introns

To find out what molecular processes lie behind the observed phenotypic changes, we selected the protein-coding genes (log_2_ value +/− 1.5) affected by *cwf14* mutation from the genes identified by Kallgren [17] and determined the GO categories to which they belong (Table S4) (PomBase database) [30]. Unexpectedly, most genes were upregulated (106 out of 117 genes; 90%), and many of them encoded proteins with oxidoreductase, transferase, hydrolase, and transporter activity (Table S4 and Table 1).

The genes involved in transport processes were also supported by GO enrichment analysis, which primarily highlighted genes related to urea (GO:0015840) (Number of genes/pathway genes: 3/3) and putrescine transport (GO:0015847) (Number of genes/pathway genes: 3/3).

In addition, we also examined the presence of introns in the protein-coding genes (PomBase) [30], because the Cwf14 protein has been associated with the spliceosome complex [21], and physical interaction was found between the proteins encoded by *cwf14* and *cdc5* genes (Prp19 splicing complex subunit Cdc5/Cef1) [17,62]. Most of the protein-coding genes unexpectedly contained no introns (95 of the 117 genes (81%)) (Table S4; black letters).

### 3.3. The cwf14 Gene May Be Linked to TOR Pathway-Regulated Processes

Because of the sensitivity of the mutant strain to rapamycin (Figure 1j), and because the TOR (Target Of Rapamycin) pathway regulates cell growth, stress response, and nutrient transport (reviewed in [63,64]), we hypothesized a link between *cwf14* and TOR signaling. To obtain evidence for this relationship, we examined further processes in the mutant that are regulated by TOR, such as chronological senescence and sporulation efficiency. In a long-term survival assay, four-week-old *cwf14* mutant cells showed reduced colony-forming ability (57%) compared to the wild-type strain (99%), indicating a decrease in viability of mutant cells. Similarly, the sporulation of the *cwf14* deleted *h^90^* strain (2-1532) was also altered, and cells showed an increased tendency to sporulate. That is, the mutant cells conjugated and produced asci both on nutrient-rich (YEA) and minimal (EMMA-nitrogen) media after only 1 day, in contrast to the wild-type *h^90^* strain, which at that time contained only vegetative cells (Figure S2). This result was reminiscent of the behavior of the *tor2* mutant cells, which mimic N starvation, which induces sporulation [65,66,67]. Therefore, we wanted to know whether there were any genes responsive to N starvation among the genes affected by the *cwf14* mutation. Thus, we compared the genes in Table S4 with those genes whose mRNA [68] or protein levels [69] were increased after N starvation. As shown in Table 2, we found overlapping genes, and interestingly, they encoded proteins with hydrolase or oxidoreductase activity.

**Figure 3:**
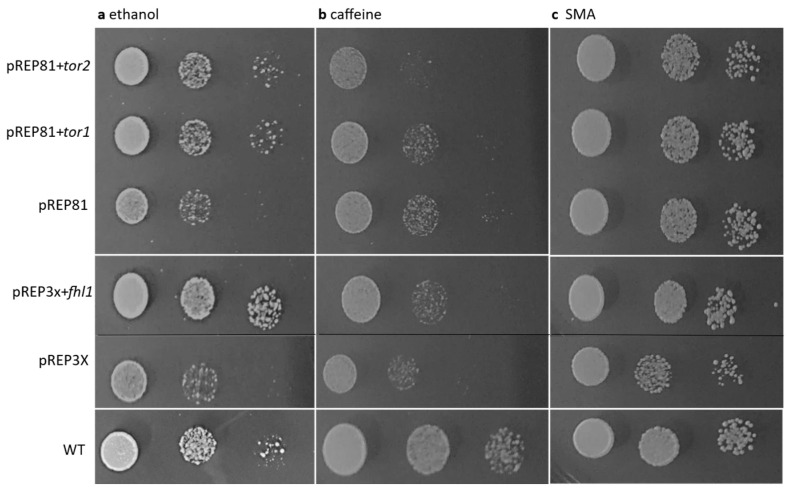
The effect of TOR-associated genes on the growth of *cwf14* mutant cells in the presence of ethanol and caffeine. *tor1*, *tor2*, and *fhl1* wild-type genes cloned into pREP vectors (pREP81, pREP3X) were transformed into *cwf14*Δ*::kanMX6 leu1-32* cells (2-1530). Growth of transformants was tested on ethanol and caffeine-containing minimal media (SMA + 8% ethanol) (**a**), (SMA + 12 mM caffeine) (**b**) (nmt promoter is induced). The strains were cultured on the same medium for 5 days at 30 °C. WT: wild-type strain (0-3). Cell density decreases from left to right because of serial dilutions of the OD_590_: 0.2 cell suspension. (**c**) shows the growth on the unsupplemented control medium (SMA).

To further test the possible *cwf14*-TOR link, we examined the effect of three regulatory genes which are involved in the TOR pathway on the *cwf14* mutant phenotype. The previously cloned *tor1*, *tor2*, and *fhl1* genes (pREP vector) [70] were transformed into *cwf14* disrupted *leu-32^-^* cells (2-1530). Although we did not analyze protein levels, we expected that the expression of *tor1*, *tor2*, and *fhl1* genes from the nmt-promoter containing pREP vector [41] would result in the overexpression of these genes. The *tor1* and *tor2* genes encode protein kinases, the components of TORC complexes [63,71,72], while the *fhl1* gene encodes a DNA-binding fork-head transcription factor, which is a downstream regulator of the TORC1 pathway [70]. Our results revealed that the presence of *S. pombe tor1*, *tor2*, and *fhl1* genes improved the growth on ethanol-containing medium in the mutant (*cwf14* disrupted) cells, compared to the control cells (transformed with empty pREP81, or pREP3X vectors) (Figure 3a). The results also indicated differences in promoter strength, as *fhl1* cloned into the pREP3X vector with a strong *nmt1* promoter resulting in stronger growth than genes cloned into the pREP81 vector with a weak promoter [41]. However, caffeine sensitivity was unchanged or increased in the presence of the *tor2* gene, which resulted in weaker growth compared to the strain transformed with the pREP81 empty vector (Figure 3b). The growth of all transformed strains was weaker than that of the wild-type strain and growth on unsupplemented control medium (Figure 3c).

Later, transcriptional profiling data obtained in *cwf14* and *tor1*, *tor2*, or *fhl1* mutants were also compared [17,65,70,73] to see if they might have overlapping genes. And indeed, there were overlaps between the genes influenced by the *cwf14* mutation and the genes associated with the TOR pathway (Table S5). The question also arose as to whether intron retention occurred in the case of the intron-containing genes (SPBPB21E7.11, SPBC1348.12) (Table S5). Examination of the exon–exon junction data obtained by Kallgren showed that there was no change in the removal of introns in these genes [17]. We also examined whether there were any TOR pathway-related genes whose RNA levels did not change significantly but which showed intron retention. As shown in Table S6, for some genes, such as *gad8*, *tco89*, and genes affected by *tor2* or *fhl1*, the ratio of exon–exon junctions in the RNA sequence was lower than 1, suggesting intron retention.

### 3.4. BUD31 Homologous Genes Are Found in Various Species, Are Evolutionarily Conserved, and Preserve Functional Homology

**Figure 4:**
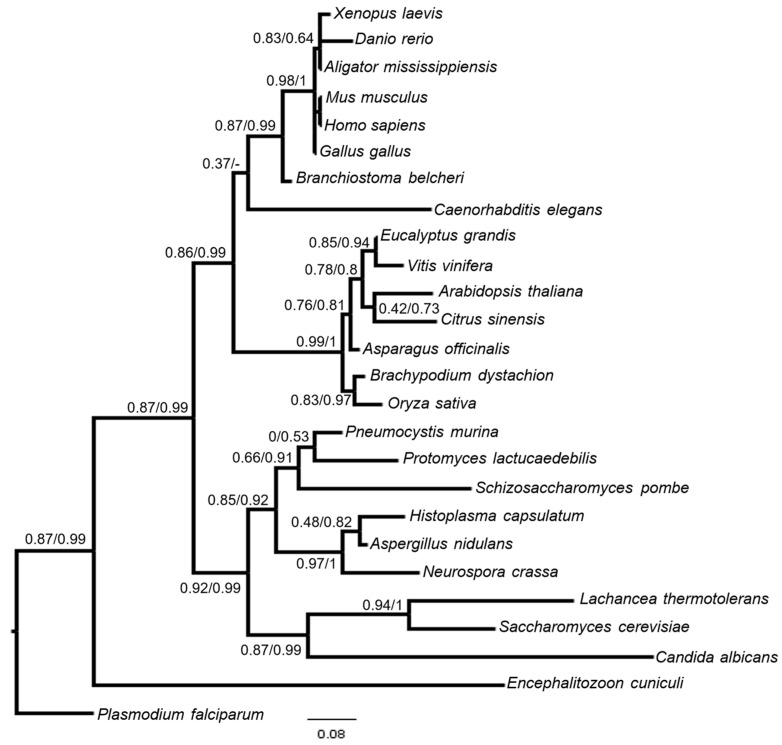
Phylogenetic analysis of Cwf14 (BUD31) putative orthologous protein sequences. The topology of the tree broadly coincided with the known phylogenetic positions of the species. Branch support came from aLRT in the case of Maximum likelihood analyses (PhyML) and posterior probability values in the case of Bayesian inference (MrBayes).

To obtain further data on BUD31 proteins and the genes encoding them, the protein sequences were investigated by in silico methods. Since the gene order and Locally Collinear Blocks (LCBs) in the fission yeast clade were previously determined [74,75], we were able to verify the localization of the *cwf14* gene in the closely related fission yeast species. It turned out that this gene is located in one of the aLCBs (ancestral Locally Collinear Blocks), which are characterized by the same gene order in the related fission yeast species and might be inherited from their last common ancestor [75]. Further BLASTp and HMMER searches revealed that putative orthologous sequences of the Cwf14 protein can be found most probably in every lineage of Eukaryotes, but not in Bacteria or Archaea, with high coverage and sequence similarity (Figure S3). We extracted 128 putative orthologs from the main kingdoms of Eukaryotes (Protista, Fungi, Animalia, and Plantae) (Table S7), and their sequences were validated by reciprocal BLASTp. The BUD31 proteins could be found in various species, from the microsporidia to the plants, animals, and humans (Figure 4 and Table S7).

The phylogenetic analyses of the 128 and 26 selected species were also performed with different algorithms (Figure 4 and Figure S4). The analyses showed that the evolution of the Cwf14 sequences broadly coincided with the evolution of the main eukaryotic kingdoms and divisions (Figure 4). However, the topologies of the phylogenetic trees within divisions did not correspond clearly to the known phylogenetic relationships of the species (Figure S4). The former phenomenon and the substantially different branch support values of the trees indicated that species-specific residue changes could occur (especially among fungi and protists) (Figure S4).

**Figure 5:**
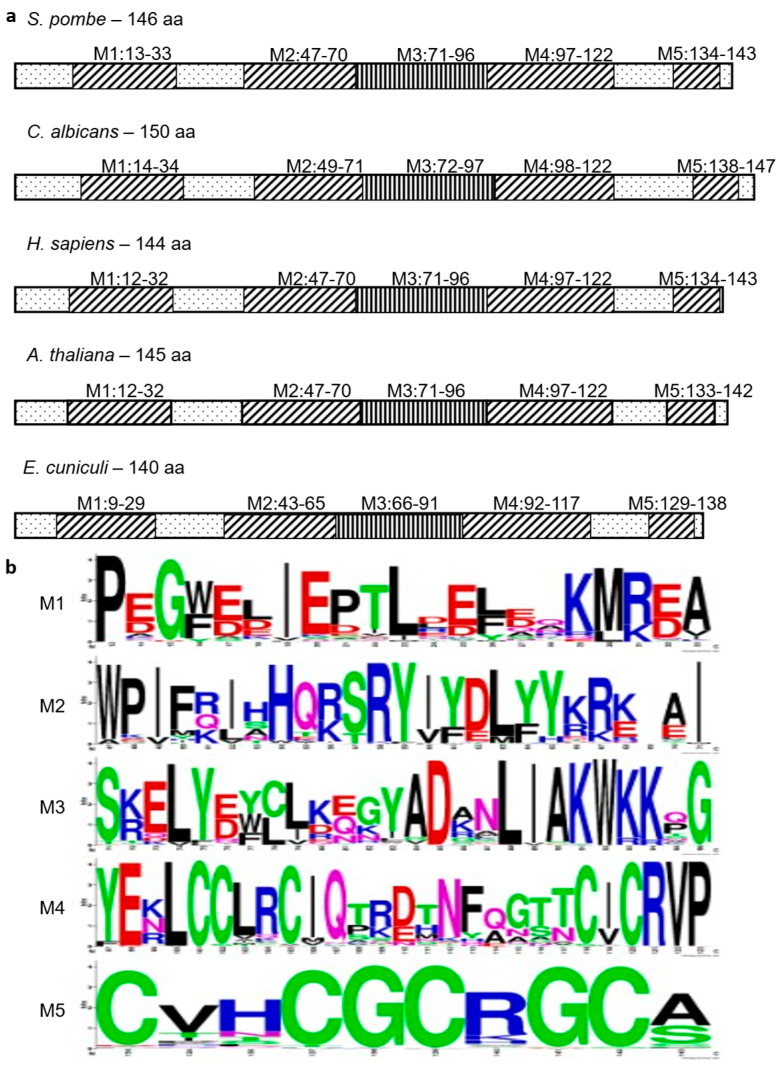
Localization of the five signature motifs in the *S. pombe* Cwf14 and in some other counterpart proteins (**a**). All the proteins contained the five motifs (M1–M5) among the investigated sequences (128 sequences were examined)(aa:amino acid). Sequence logos of the five signature motifs of the 128 examined Cwf14 putative orthologous sequences. The motif at the C-terminal of the proteins (M5) (zinc ion cluster) is the most conserved region (**b**).

Investigation of the selected protein sequences also showed that their size ranged from 129 aa to 184 aa, but in most cases, they were in the 144–146 aa size range. The sequences showed global conservation among the species, but with unique stretches and deletions (Figure S5). The comparative sequence analyses clearly showed that these proteins have 5 signature motifs (Figure 5a). Although the motifs can be found in almost every lineage, there are many lineage-specific sites (Figure 5b and Figure S5). The C-terminal cysteine residues are especially interesting and belong to the most conservative sites of the protein sequences (zinc ion cluster) (Figure 5b).

Later, the 3D predictions of the protein sequences were also tested. Phyre2 and Swissmodel predictions were created using different templates according to the best model for the concerned sequences. Since cryo-EM and MR templates exist (see Methods), 99% of the residues could be modeled at >90% confidence. These predictions showed extreme conservation among the protein structures of the different species and revealed that the structure of the Cwf14 protein is almost the same in the different eukaryotes (Figure S6).

**Figure 6:**
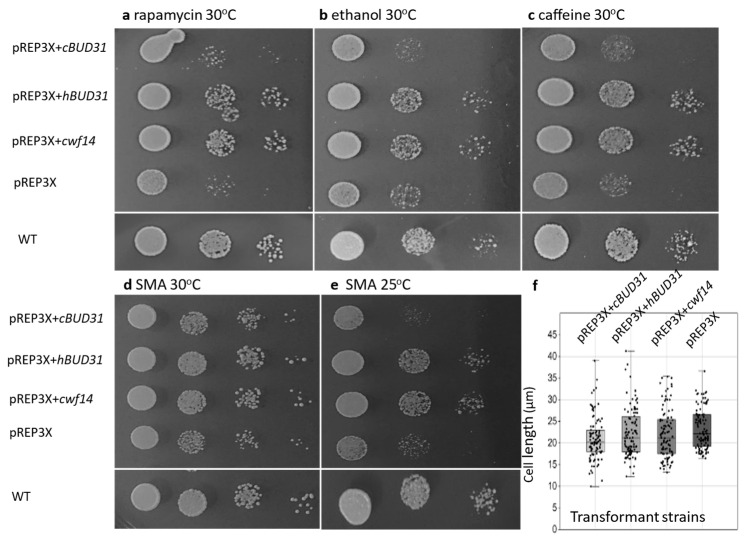
Interspecific complementation analysis. *S. pombe* mutant cells (2-1530) were transformed with pREP vectors containing human *BUD31* (pREP3X + *hBUD31*), *C. albicans BUD31* (pREP3X + *cBUD31*), and *S. pombe cwf14* genes (positive control). The empty vector (pREP3X) was used as a negative control. The cells were spread on SMA containing rapamycin (**a**), 8% ethanol (**b**), 12 mM caffeine (**c**) media, and SMA without any supplementation (**d**). The Petri dishes were incubated at 30 °C for 4 days) (**a**–**d**), and at 25 °C (**e**). WT: wild-type cells (0-3). Cell length was also measured for all transformants and analyzed statistically (**f**). Although the presence of the *cBUD31* gene did not improve propagation of transformant cells, it significantly reduced cell length at 37 °C (SMA) similar to *hBUD31* and *cwf14* and different from cells containing the empty pREP3X vector (Kruskal–Wallis test, *p* = 0.01143) (the *C. albicans* orthologous protein (*cBUD31*) resulted in a significantly reduced cell length compared to the others) (according to the Bonferroni corrected Dunn’s Post Hoc test (*p* = 0.0062).

Based on these bioinformatic results, the question arose as to whether these counterpart proteins have also preserved functional homology or not. To answer this question, an interspecific complementation test was performed with two putative orthologous genes of *cwf14* (*cBUD31*-*Candida albicans*, *hBUD31*-human), whose gene products showed a considerable sequence identity to the *S. pombe* Cwf14 protein (*S. pombe*-*C. albicans*: 48%, *S. pombe*-human: 59%) (Figure S7), were selected and cloned into pREP *S. pombe*-specific expression vectors [41]. The respective plasmids were transformed into *cwf14* mutant (2-1530) *S. pombe* cells. The growth of the transformant cells was tested with a drop assay test, while their cell size was examined under a microscope. Overexpression of the human *BUD31* gene (pREP3X-*hBUD31*) could partly complement the rapamycin—(Figure 6a), ethanol—(Figure 6b), and caffeine sensitivity (Figure 6c). These transformed cells grew better than cells transformed with the empty vector pREP3X. Their growth was similar to that of cells transformed with the wild-type *cwf14* gene of *S. pombe* (pREP3X-*cwf14*) (positive control) and growth on control medium without supplements (Figure 6d). The slow-growth phenotype of *cwf14* mutant cells at 25 °C (SMA) (Figure 6e) was again improved by *hBUD31*. In contrast, *cBUD31* did not complement (pREP3X-*cBUD31*) stress sensitivity. However, we cannot say that *cBUD31* did not work, because when we measured the cell size of the transformants, all three genes (*cwf14*, *cBUD31*, *hBUD31*) caused changes. Their overexpression resulted in significantly shorter cell size at 37 °C (Figure 6f), and longer size at 30 °C (the average cell size changed from 13 µm to 18–19 µm), compared to the control cells transformed with the empty vector. Similarly, the cell morphology on 12 mM caffeine-containing medium was also improved in all cases (ratio of the normal morphology was 51% in the strain transformed with the empty pREP3X vector, while more than 70% in the other cases (74%-pREP3X + *cwf14*, 71%-pREP3X + *hBUD31*, 74%-pREP3X + *cBUD31*). Although the superposition of the predicted protein structures indicates highly conserved 3D layouts (Figure S6), the different orthologs showed somewhat different complementation efficiency.

## 4. Discussion

Here, we focused on the *S. pombe cwf14* gene, which is homologous to the human *BUD31*. Previous studies have shown that mutations of this human gene are associated with various types of cancer, often with poor survival rates [1,2,3,4,5,6]. Therefore, there is a great need to understand how this gene functions and what can cause the aforementioned changes. Thus, the *S. pombe cwf14* mutant strain was examined using genetic and in silico methods.

Phenotypic characterization of *S. pombe* mutant strains revealed a slow growth phenotype (Figure 1d,e, Figure 2 and Figure S1) and longer cell size at different temperatures (Figure 1a,f), which confirms and complements previous results [15,16] and is similar to the changes found in budding yeast cells [8,9,14]. These data indicated cell cycle problems, which were supported by the fact that we found multiseptated morphology (Figure 1b) instead of single cells (Figure 1c) on caffeine-containing medium, which can exacerbate abnormalities in cytokinesis (cell separation after mitosis), if such a problem exists [76]. This morphology resembled that of *sep* mutants with cytokinesis defects [27,28,29]. Furthermore, examination of sporulation showed that although the mutant cells were capable of conjugating and forming spores (Figure 1g), meiosis was not completely regular, as chromosomes often segregated incorrectly in the spores. That is, *S. pombe cwf14* gene appears to be involved in cell division, which is consistent with cancerous changes in human cells [1,2,3,4,5], as cancer is a disease that involves abnormal cell growth and cell division.

To find the causes of cell division problems, protein-coding genes influenced by *cwf14* (higher log_2_ value than +/− 1.5) [17] were selected and examined. Interestingly, most genes were upregulated (106 out of 117 genes) in the *cwf14* mutant (Table S4). When we determined their GO categories based on PomBase [30], unexpectedly, most of them encoded proteins with transporter-, hydrolase-, oxidoreductase-, and transferase activity (Table 1 and Table S4). GO enrichment analysis also highlighted transporters, particularly involved in urea and putrescine transport, which are descendants of nitrogen compound transport (GO:0071705) and control the transport of nitrogen-containing compounds. Changes in transport processes may explain abnormalities in cell division and growth, as nutrient supply, especially its limitation, alters the regulation of cell division [77,78]. That is, nutrient supply, cell size, and cell division are related to each other reviewed in [77].

However, the question arose as to why genes encoding transporters and enzymes are upregulated compared to control cells when the *cwf14* strain was grown on YES medium [17], which is a nutrient-rich medium and provides optimal conditions for growth and division. One scenario could be that the cells feel as if they are starving despite nutrient-rich conditions and therefore increase transport processes. Since GO enrichment analysis highlighted genes involved in nitrogen compound transport, and because we found several N-sensitive genes among the genes affected by *cwf14* mutation (Table 2), we assume that there may be a nitrogen-sensing problem in the absence of the *cwf14* gene. Most of the latter genes were upregulated, similar to previous experiments where mRNA or protein levels encoded by these genes increased after N deprivation [68,69]. The problem of N sensing is also supported by our observation that *cwf14* mutant cells formed spores after only one day on both nutrient-rich (YEA) and N-deficient media (EMMA-N) (which contained only vegetative cells in the case of the wild-type strain) (Figure S2), since nitrogen deficiency induces sporulation in *S. pombe* [67]. A similar phenotype was observed in *tor2* mutant cells, where loss of *tor2* function mimicked nitrogen starvation and induced transporter genes [65,66]. Increased transporter activity can compensate for real or mimicked nutrient deficiencies, but why did hydrolases or oxidoreductases appear (Table 1)? We assume that they may play a role in nitrogen mobilization, like in plants, where N deficiency can induce degradation of N-containing compounds (reviewed in [79]); however, this idea requires further investigation.

The similarity to the behavior of *tor2* mutant cells drew our attention to the TOR pathway, which is a complex network and a master regulator of various cellular processes, e.g., growth and response to nutrient signals (reviewed in [64]). The *cwf14*-TOR relationship was supported by the sensitivity of the *cwf14*Δ*::kanMX6* strain to rapamycin (Figure 1j), which is consistent with Doi’s previous results [60]. However, it should be noted that this differs from the results of Rodríguez-López, who demonstrated rapamycin resistance under other conditions [61]. The *cwf14*-TOR link was also indicated by the findings that additional cellular processes regulated by the TOR pathway, such as stress sensitivity and aging [80,81], were also altered in the *cwf14* mutant strain. It was sensitive to ethanol and caffeine (Figure 1h,i), which is in good agreement with previous results [60,61] and with data from the *S. cerevisiae* mutant strain that also showed an altered stress response to ethanol [11,82,83]. The increased chronological aging of *cwf14*^-^ cells also confirmed the results of a high-throughput assay that identified aging-associated genes [84].

To obtain further evidence for the *cwf14*-TOR link, we examined the effect of overexpression of *tor1*, *tor2* (key regulators of the TOR pathway) (reviewed in [64]), as well as *fhl1* (an additional TORC1-related gene) [70] on the *cwf14* mutant phenotype. Our data suggest that the presence of TOR-associated genes improved the growth of mutant cells on ethanol-containing medium (Figure 3a). Furthermore, the extent of complementation depended on the strength of the promoters, as *fhl1*, which was cloned into a vector with a stronger *nmt1* promoter (pREP3X), caused more vigorous growth than the *tor1* and *tor2* genes (which were cloned into a vector with a weaker promoter) (pREP81) [41]. Regarding caffeine sensitivity, it was only affected by the *tor2* gene (member of the TORC1 pathway), which increased sensitivity (Figure 3b).

Since *S. pombe* has two TORC complexes (TORC1 containing Tor2 protein and TORC2 containing Tor1p) (reviewed in [64]), we were curious to see which one *cwf14* might be closer to. Our results suggest that this cannot be clearly determined because the *cwf14* gene shows a relationship with both TORC complexes. However, the increase in sporulation propensity (Figure S2) and rapamycin sensitivity of *cwf14*^-^ cells (Figure 1j) more closely resembled those of *tor2* mutant cells [65,66]. Namely, the *tor1* and *tor2* mutants show opposite phenotypes after N-starvation (*tor1*Δ cells fail to undergo meiosis, while the *tor2* mutant strongly sporulates) [85], and *tor2* cells are more sensitive, while *tor1* mutants are less sensitive or completely insensitive to rapamycin inhibition [65], reviewed in [63,64]. At the same time, we also found a correlation between *cwf14* and *tor1*. When we compared the genes affected by *cwf14* with those affected by *tor1*, *tor2*, or *fhl1* [17,65,70], we found overlapping genes not only with *tor2* and *fhl1* (TORC1) but also with *tor1* (TORC2) (Table S5). These results reflect the complexity of environmental sensing and TOR signal transduction and may arise from the fact that the functions of the TORC1 and TORC2 complexes are partly different and partly overlapping (reviewed in [63,64]).

Since the *cwf14* gene and its homologues were required for splicing of certain mRNAs [9,17,21,24,86,87], we hypothesized that abnormal removal of introns from genes affected by *cwf14* deletion caused the observed phenotypic changes. However, this is contradicted by the fact that when we examined the DNA sequence of genes affected by the *cwf14* mutation, most of them contained no introns (Table S4). Thus, we assume that most protein-coding genes were only indirectly affected by the *cwf14* mutation, since in their cases we cannot speak of defective intron removal. Presumably, the altered transcription of these genes could have been caused by the defect of splicing of another intron-containing gene or genes. This is supported by the fact that based on data from Kallgrens’s, genes associated with the TOR pathway, such as *gad8*, *tco89*, and further genes affected by *tor2* or *fhl1* [65,70], had a ratio of exon–exon junctions in the RNA sequence lower than 1, which may indicate that intron retention occurred in the mRNAs of these genes (Table S6) [17]. It is possible that a change in intron retention in key genes (or in others) occurred in the *cwf14* mutant, even without changing the transcript RNA levels. These defects can also have a major influence on the phenotype and indirectly the entire transcriptome. In addition, intron retention has also been demonstrated in *ago1* and *arb2* [17]. Since *ago1* and *arb2* genes are involved in chromatin organization [88], it may be possible that changes in genome integrity caused the transcriptional changes in the *cwf14*-affected intronless genes, including, e.g., N-responsive genes (Table 2). Although this requires further investigation, it may also explain the TOR pathway–*cwf14* association (Figure 3, Table S5), as the TOR pathway is an important regulator of environmental sensing. Furthermore, not only the *cwf14* gene, but also TORC2 (*tor1*) plays a critical role in chromatin-mediated gene silencing and genome integrity [17,73]. Although it should be noted that, interestingly, the results of Kallgren’s showed that the majority of introns were properly processed in the *cwf14* mutant strain, which indicates that the Cwf14 protein only moderately affected the activity of the spliceosome [17].

These data are interesting because our bioinformatic analyses have revealed that orthologues of the *cwf14* gene can be found in various species, from microsporidia to humans (Figure 4 and Figure S4). They have high sequence and structural homology (Figure 5 and Figures S5–S7). Interspecific complementation analyses have also shown that, for example, the *Candida* and human *BUD31* genes have preserved their functional homology. That is, the overexpression of the *hBUD31* gene “cured” the sensitivity of the *S. pombe cwf14*Δ*::kanMX6* cells to rapamycin, ethanol, and caffeine (Figure 6a–d). Interestingly, the *hBUD31* gene functioned better than the *Candida* gene in the mutant *S. pombe* cells in terms of stress responses (Figure 6a–c). This is because the sequences of *S. pombe* and human proteins are more similar to each other than the sequences of *S. pombe* and *Candida* (Figure S7). However, cell length was significantly influenced by both these genes (Figure 6f).

In summary, we have shown here that the *S. pombe cwf14/bud31* gene may be involved in cell division, stress response, and chronological aging, which are processes regulated by the TOR pathway. This association was also confirmed by experiments; however, further research is needed to uncover details of the *cwf14*-TOR relationship. We also revealed that there must be nutrient/nitrogen sensing problems in the mutant cells. Since the in silico and interspecific complementation analyses showed that the *BUD31* gene was evolutionarily conserved from yeast to humans and retained its functional homology, these data may contribute to a better understanding development of human tumor cells.

## 5. Conclusions

*BUD31* and its homologous genes are evolutionarily conserved and have preserved their functional homology.

The *BUD31* homologous gene in *S. pombe* is involved in cellular processes regulated by the TOR pathway.

Most of the protein-coding genes affected by the *cwf14* mutation were upregulated, involved in N-sensing, or encoded oxidoreductases and hydrolases.

## Figures and Tables

**Table 1:** GO categories and the number of their genes.

Number of Genes	GO Categories
13	oxidoreductase activity (GO:0016491)
13	hydrolase activity (GO:0016787), hydrolase activity, acting on glycosyl bonds (GO:0016798), hydrolase activity, acting on carbon-nitrogen (but not peptide) bonds (GO:0016810), hydrolase activity, acting on ester bonds (GO:0016788), ATP hydrolysis activity (GO:0016887)
12	nucleotidyltransferase activity (GO:0016779), glycosyltransferase activity (GO:0016757), acyltransferase activity (GO:0016746), transferase activity, transferring one-carbon groups (GO:0016741), transferase activity, transferring alkyl or aryl (other than methyl) groups (GO:0016765), transferase activity, transferring one-carbon groups (GO:0016741)
9	transmembrane transporter activity (GO:0022857)
1	vesicle-mediated transport (GO:0016192)
3	endomembrane system (GO:0012505), plasma membrane (GO:0005886)
2	lyase activity (GO:0016829)
2	isomerase activity (GO:0016853)

**Table 2:** Nitrogen-responsive genes influenced by *cwf14* mutation.

Gene Identifier	Gene Name	Description	GO Category	Source
SPAC869.04		formamidase-like protein, implicated in cellular detoxification	hydrolase activity, acting on carbon-nitrogen (but not peptide) bonds (GO:0016810)	[68]
SPBC1683.06c	*urh1*	uridine ribohydrolase Urh1	hydrolase activity, acting on glycosyl bonds (GO:0016798)	[68]
SPBC1683.02		adenine deaminase	hydrolase activity, acting on glycosyl bonds (GO:0016798)	[68]
SPAC11D3.14c	*oxp2*	5-oxoprolinase (ATP-hydrolizing)	hydrolase activity, acting on carbon-nitrogen (but not peptide) bonds (GO:0016810)	[68]
SPAC186.06		phenazine biosynthesis PhzF protein family	isomerase activity (GO:0016853)	[68]
SPBPB2B2.06c	*efn1*	extracellular 5′-nucleotidase, human NT5E family	hydrolase activity, acting on ester bonds (GO:0016788)	[69]
SPAC3A11.10c	*dpe1*	dipeptidyl peptidase, unknown specificity, implicated in glutathione metabolism	peptidase activity (GO:0008233)	[69]
SPBC725.03		pyridoxamine 5′-phosphate oxidase	oxidoreductase activity (GO:0016491)	[69]
SPAC23C11.06c		vacuolar membrane hydrolase, implicated in protein catabolism or lipid metabolism	hydrolase activity (GO:0016787)	[69]
SPAC139.05	*ssd2*	succinate-semialdehyde dehydrogenase	oxidoreductase activity (GO:0016491)	[69]
SPBC16A3.02c		mitochondrial CH-OH group oxidoreductase, human RTN4IP1 ortholog, implicated in mitochondrial organization or tethering	oxidoreductase activity (GO:0016491)	[69]

mRNA levels [68] or protein levels encoded by these genes [69] increased after N starvation. With the exception of SPAC186.06, mRNA levels were upregulated [17].

## Data Availability

All data generated or analyzed during this study are included in this published article and its Supplementary Information Files.

## Supplementary Materials

The following supporting information can be downloaded at: https://www.mdpi.com/article/10.3390/cells14211736/s1, Figure S1: The growth of *cwf14* mutant cells (2-1480) (YEL, at 30 °C). Control: wild-type cells (0-1); Figure S2: Increased sporulation efficiency of the mutant strain (2-1530, h^90^ mating type) (**a**,**c**). Control: the wild-type h^90^ strain (0-3) (**b**,**d**). The cells were cultured on YEA (30 °C, 1 day) (**a**,**b**), and EMMA-N (30 °C, 1 day) (**c**,**d**). The white arrows show the zygotes and asci; Figure S3: BLAST EXPLORER results using the *S. pombe* Cwf14 protein sequence as query. The search was performed in the non-redundant protein database of NCBI, and it resulted in 1197 hits. (**a**) The histogram shows the distribution of the coverage among the hits. More than 80% of the found sequences had a query coverage larger than 90%. (**b**). The histogram depicts the distribution of sequence similarity among the found sequences. Most of the sequences showed more than 55% similarity to the query sequence; Figure S4: The large-scale phylogeny of 128 putative Cwf14 (BUD31) orthologous protein sequences. The created tree indicates that the Cwf14 sequences are common among the Eukaryotes, and the topology of the tree coincides largely with the current phylogenetic distribution of the main groups. However, many species within the main divisions are placed incorrectly (e.g., common branching of *Encephalitozoon cuniculi* and *Plasmodium falciparum* with *Saccharomycotina* yeasts). Apart from the misplacements, the main kingdoms are well separated. The brownish highlight indicates the kingdom Animalia, the greenish depicts the kingdom Plantae, and the orange-yellowish highlights the kingdom Fungi. Other non-highlighted branches are simple Eukaryotes. The tree is created with PhyML 3.0 (maximum likelihood) and is a cladogram; therefore, the lengths of the branches are not informative. Branch support came from aLRT analysis; Figure S5: Weblogos generated from the muscle alignments of the Cwf14 (BUD31) putative orthologous protein sequences. The created Weblogos show the most abundant amino acid residues per site within the Cwf14 orthologous sequences; Figure S6: Protein structure prediction of several Cwf14 (BUD31) putative orthologs. As cryo-EM and MR templates exist, 99% of the residues could be modeled with >90% confidence. The depicted structures show that the *S. pombe* Cwf14 and its putative orthologous proteins exhibit almost identical protein structures. Structure predictions were made with the Phyre2 server and visualized with the UCSF Chimera software (v 1.13); Figure S7: Significant sequence identity was found between the *S. pombe* Cwf14 protein and human and *Candida* BUD31p (BLASTp analysis of protein sequences). *Schizosaccharomyces pombe* Cwf14p (Query)-*Candida albicans* BUD31p (Sbjct) (**a**), *Schizosaccharomyces pombe* Cwf14p (Query)-human BUD31p (Sbjct) (**b**); Table S1: Strains used in this study; Table S2: Primers used in this study; Table S3: Plasmids used in this study; Table S4: Protein-coding target genes of *cwf14**. The majority of genes do not contain introns (black letters) (genes containing introns are marked in green letters).* [17]; ** [26,30]; WT: wild type); Table S5: Selected genes with significantly altered mRNA levels in the *cwf14* and, in *tor1*, *tor2*, or *fhl1* mutant strains; Table S6: TOR pathway-associated genes showing intron retention; Table S7: BUD31 homologous sequences of the various species.
