# Supplementary material for: The BUD31 Homologous Gene in Schizosaccharomyces pombe Is Evolutionarily Conserved and Can Be Linked to Cellular Processes Regulated by the TOR Pathway
Source: Cells. 2025 Nov 5;14(21):1736. doi: 10.3390/cells14211736 (PMC12610034; doi:10.3390/cells14211736)
Supplement: Supplementary file 1 [file cells-14-01736-s001.zip › Supplemental figures.pdf]

Supplemental figures

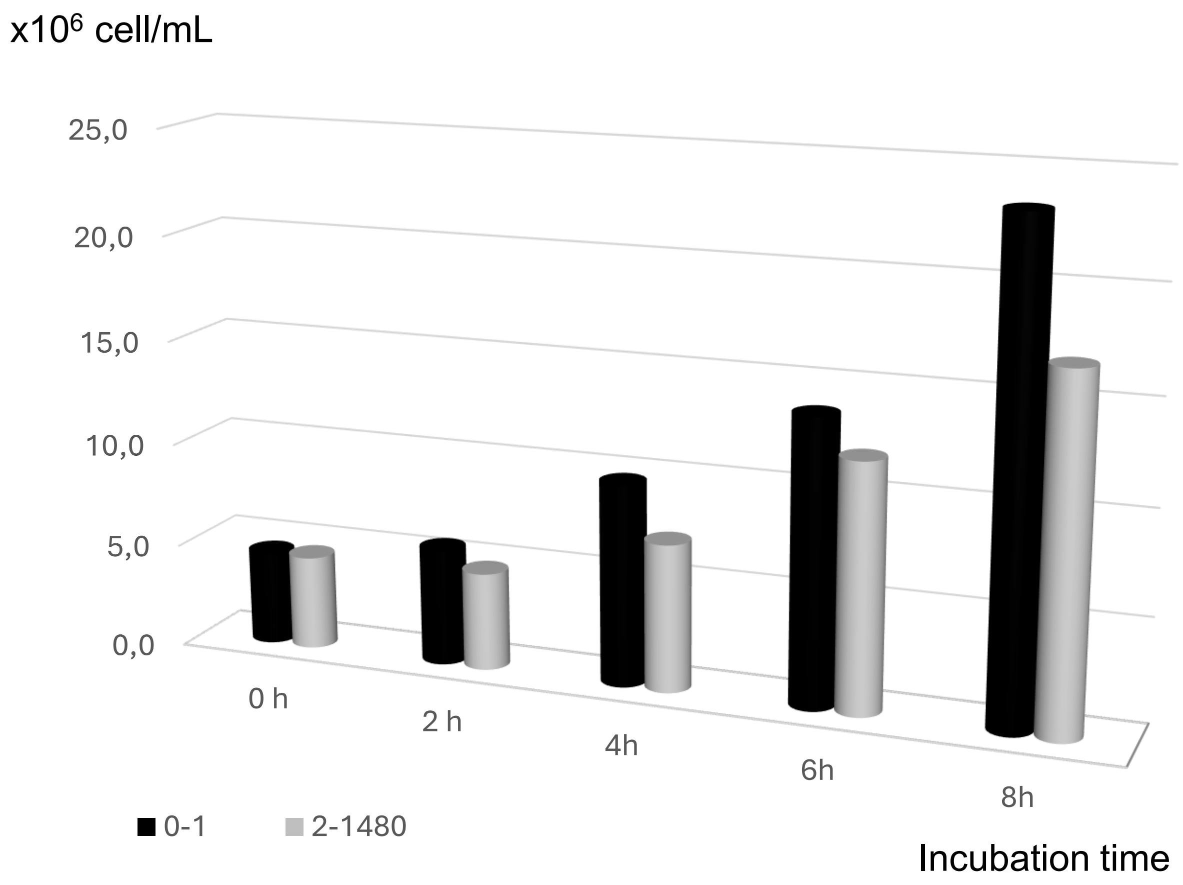

Figure S1. The growth of *cwf14* mutant cells (2-1480) (YEL, at 30°C). Control: wild-type cells (0-1).

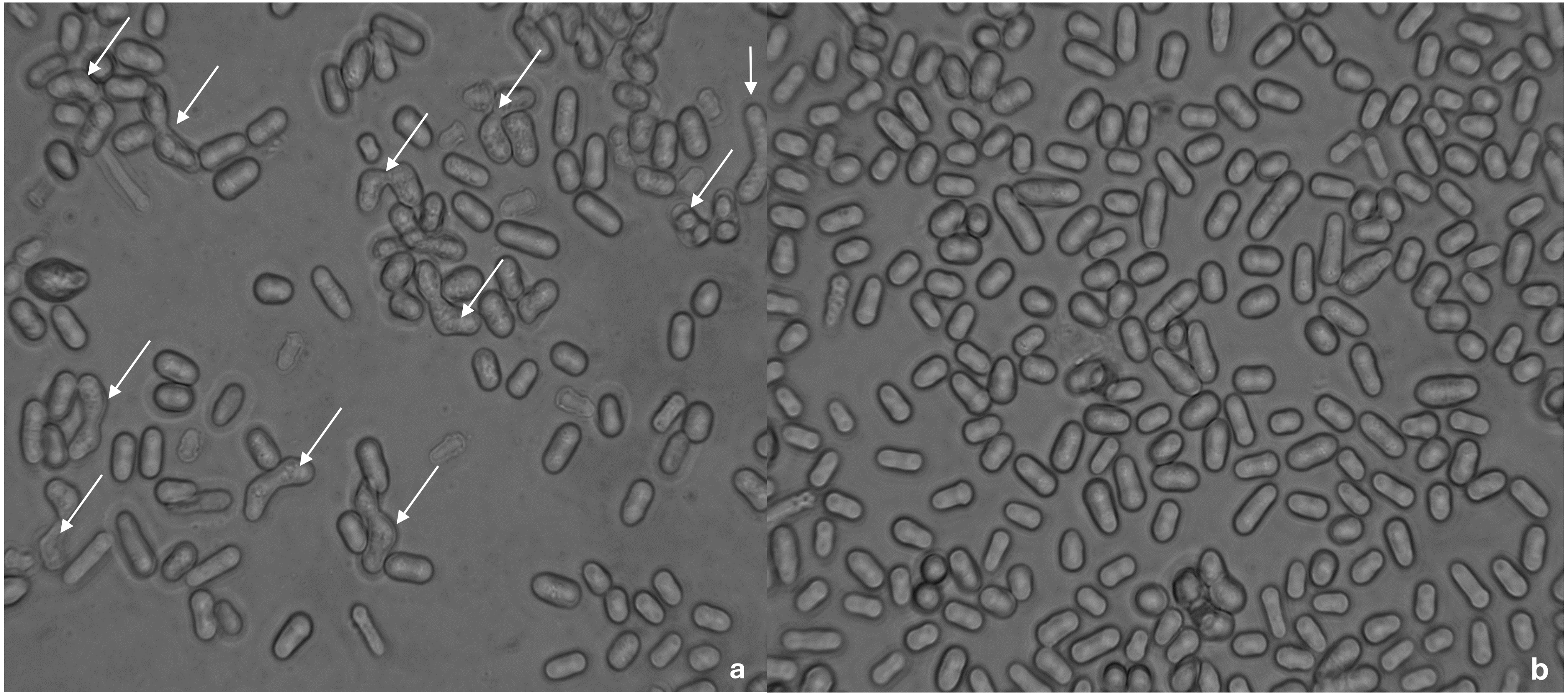

Figure S2. Increased sporulation efficiency of the mutant strain (2-1530, *h*[90] mating type) (a, c). Control: the wild-type *h*[90] strain (0-3) (b, d). The cells were cultured on YEA (30°C, 1 day) (a, b), and EMMA-N (30°C, 1 day) (c, d). The white arrows show the zygotes and asci.

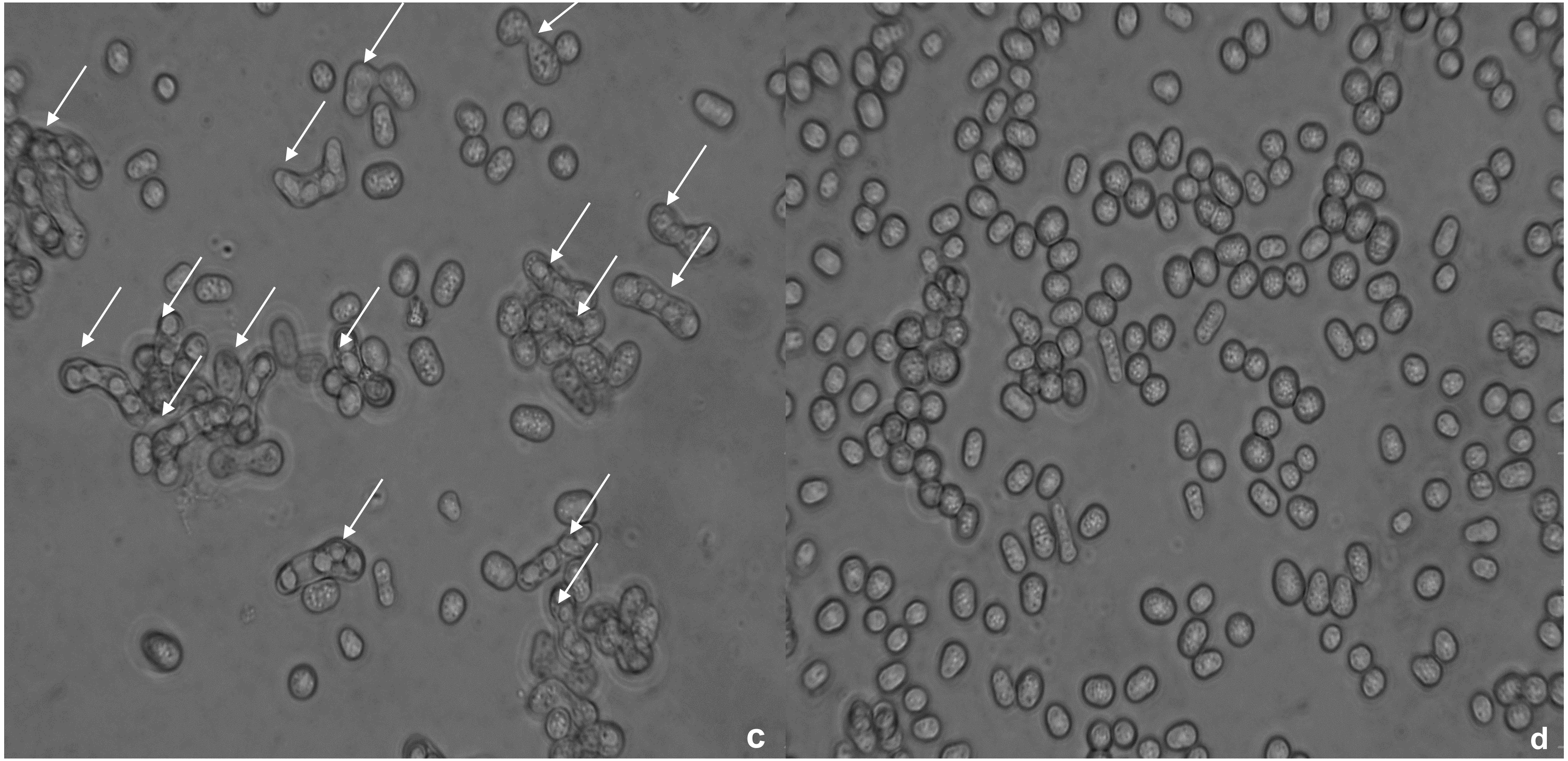

Figure S2. Increased sporulation efficiency of the mutant strain (2-1530, h^{90} mating type) (a, c). Control: the wild-type h^{90} strain (0-3) (b, d). The cells were cultured on YEA (30°C, 1 day) (a, b), and EMMA-N (30°C, 1 day) (c, d). The white arrows show the zygotes and asci.

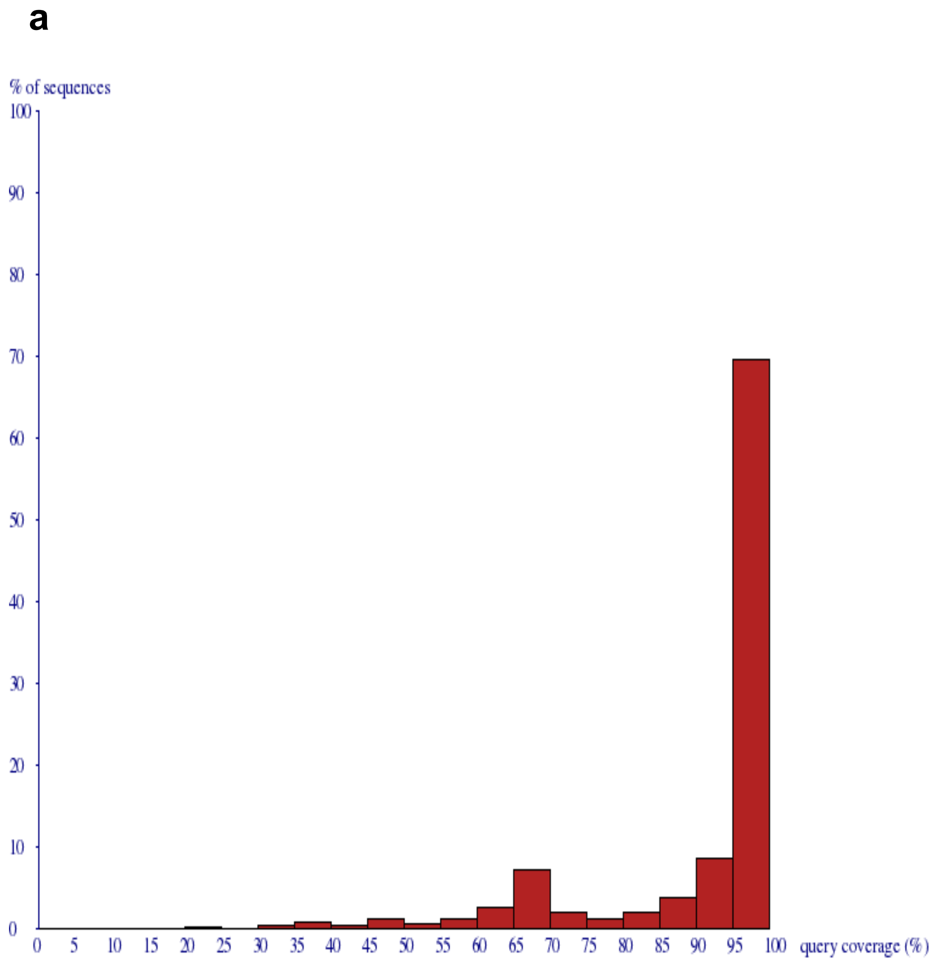

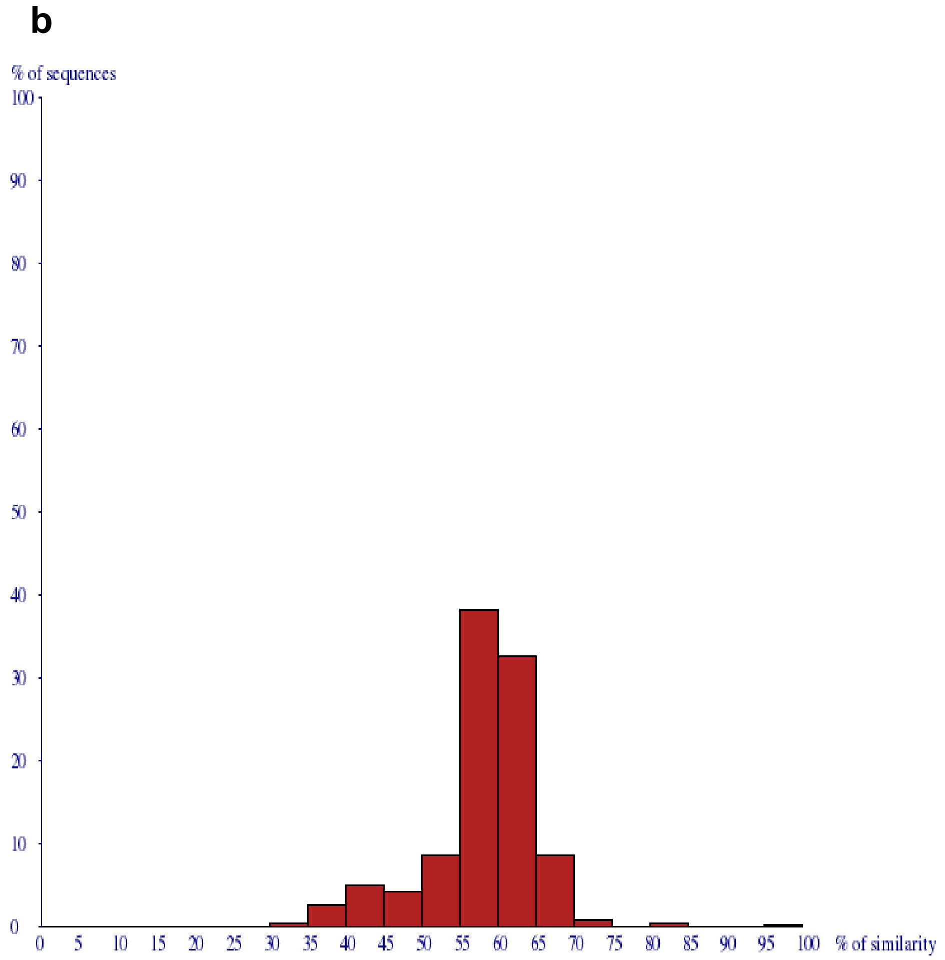

Figure S3. BLAST EXPLORER results using the *S. pombe* Cwf14 protein sequence as query. The search was performed in the non-redundant protein database of NCBI, and it resulted in 1197 hits. (a) The histogram shows the distribution of the coverage among the hits. More than 80% of the found sequences had a query coverage larger than 90%. (b). The histogram depicts the distribution of sequence similarity among the found sequences. Most of the sequences showed more than 55% similarity to the query sequence.

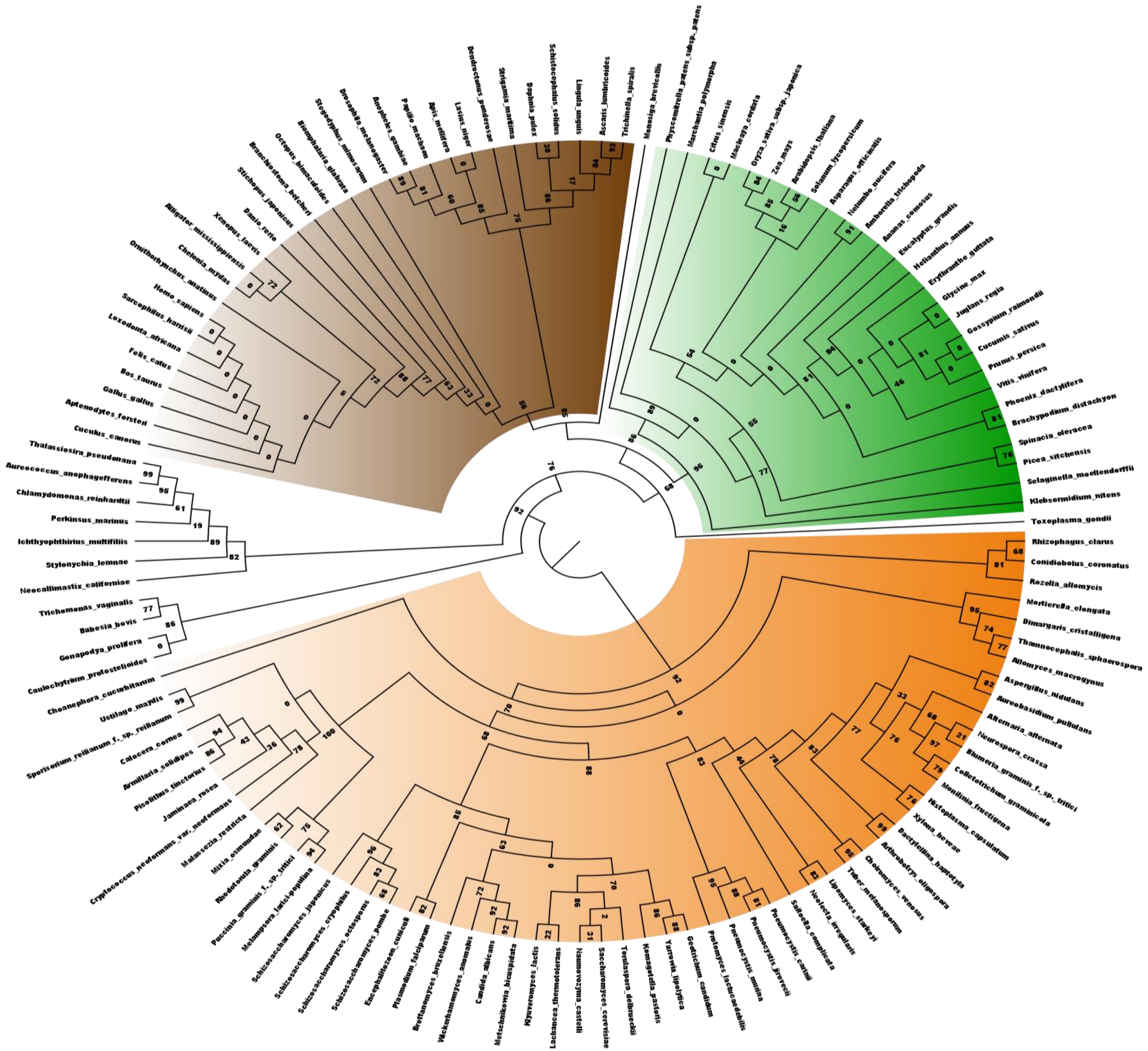

Figure S4. The large-scale phylogeny of 128 putative Cwf14 (BUD31) orthologous protein sequences. The created tree indicates that the Cwf14 sequences are common among the Eukaryotes, and the topology of the tree coincided largely with the current phylogenetic distribution of the main groups. However, many species within the main divisions are placed incorrectly (e.g., common branching of *Encephalitozoon cuniculi* and *Plasmodium falciparum* with *Saccharomycotina* yeasts). Apart from the misplacements, the main kingdoms are well separated. The brownish highlight indicates the kingdom Animalia, the greenish depicts the kingdom Plantae, and the orange-yellowish highlights the kingdom Fungi. Other non-highlighted branches are simple Eukaryotes. The tree is created with PhyML 3.0 (maximum likelihood) and is a cladogram; therefore, the lengths of the branches are not informative. Branch support came from aLRT analysis.

Figure S5. Weblogos generated from the Muscle alignments of the Cwf14 (BUD31) putative orthologous protein sequences. The created Weblogos show the most abundant amino acid residues per site within the Cwf14 orthologous sequences.
The examined species are the following:

Schizosaccharomyces (*S. japonicus, S. pombe, S. octosporus, S. cryophilus*)

Taphrinomycotina (*Neolecta irregularis, S. japonicus, S. pombe, S. octosporus, S. cryophilus, Saitoella complicata, Protomyces lactucaedebilis, Pneumcystis jirovecii, P. murina, P. carinii*)

Saccharomycotina (*Candida albicans, Metschnikowia bicuspidata, Brettanomyces bruxellensis, Wickerhamomyces anomalus, Klyuveromyces lactis, Lachancea thermotolerans, Naumovozyma castelli, Saccharomyces cerevisiae, Torulaspora delbrueckii, Komagotella pastoris, Yarrowia lipolytica, Lipomyces starkeyi, Geotrichum candidum*)

Pezizomycotina (*Monilinia fructigena, Blumeria graminis f. sp. tritici, Colletotrichum graminicola, Neurospora crassa, Arthrobotrys oligospora, Dactylellina haptotyla, Xylona heveae, Alternaria alternata, Tuber melanosporum, Choiromyces venosus, Aureobasidium pullulans, Histoplasma capsulatum, Aspergillus nidulans*)

Basidiomycota (*Rhodotorula graminis, Mixia osmundae, Puccinia graminis f. sp. tritici, Melampsora larici-populina, Malassezia restricta, Ustilago maydis, Sporisorium reilianum f. sp. reilianum, Cryptococcus neoformans var. neoformans, Calocera cornea, Jaminaea rosea, Armillaria solidipes, Pisolithus tinctorius*)

Other fungi (*Encephalitozoon cuniculi, Rozella allomycis, Caulochytrium protostelioides, Gonapodya prolifera, Allomyces macrogynus, Conidiobolus coronatus, Dimargaris cristalligena, Mortierella elongata, Thamnocephalis sphaerospora, Rhizophagus clarus, Neocallimastix californiae, Choanephora cucurbitarum*)

Kingdom Fungi (as can be seen above)

Simple eukaryotic organisms (*Trichomonas vaginalis, Thalassiosira pseudonana, Aureococcus anophagefferens, Chlamydomonas reinhardtii, Monosiga brevicollis, Toxoplasma gondii, Perkinsus marinus, Klebsormidium nitens, Stylonychia lemnae, Ichthyophthirius multifiliis, Plasmodium falciparum, Babesia bovis*)

Animalia (*Trichinella spiralis, Schistocephalus solidus, Lingula unguis, Strigamia maritima, Daphnia pulex, Stegodyphus mimosarum, Danio rerio, Xenopus laevis, Chelonia mydas, Alligator mississippiensis, Homo sapiens, Sarcophilus harrisii, Loxodonta africana, Felis catus, Bos taurus, Gallus gallus, Aptenodytes forsteri, Cuculus canorus, Octopus bimaculoides, Biomphalaria glabrata, Papilio machaon, Anopheles gambiae, Drosophila melanogaster, Stichopus japonicus, Branciostoma belcheri, Dendroctonus ponderosae, Lasius niger, Apis mellifera*)

Plantae (*Selaginella moellendorffii, Physcomitrella patens subsp. patens, Solanum lycopersicum, Marchantia polymorpha, Citrus sinensis, Arabidopsis thaliana, Picea sitchensis, Spinacia oleracea, Erythranthe guttata, Glycine max, Oryza sativa subsp. japonica, Asparagus officinalis, Brachypodium distachyon, Ananas comosus, Phoenix dactylifera, Amborella trichopoda, Nelumbo nucifera, Vitis vinifera, Juglans regia, Prunus persica, Cucumis sativus, Gossypium raimondii, Helianthus annuus, Eucalyptus grandis*)

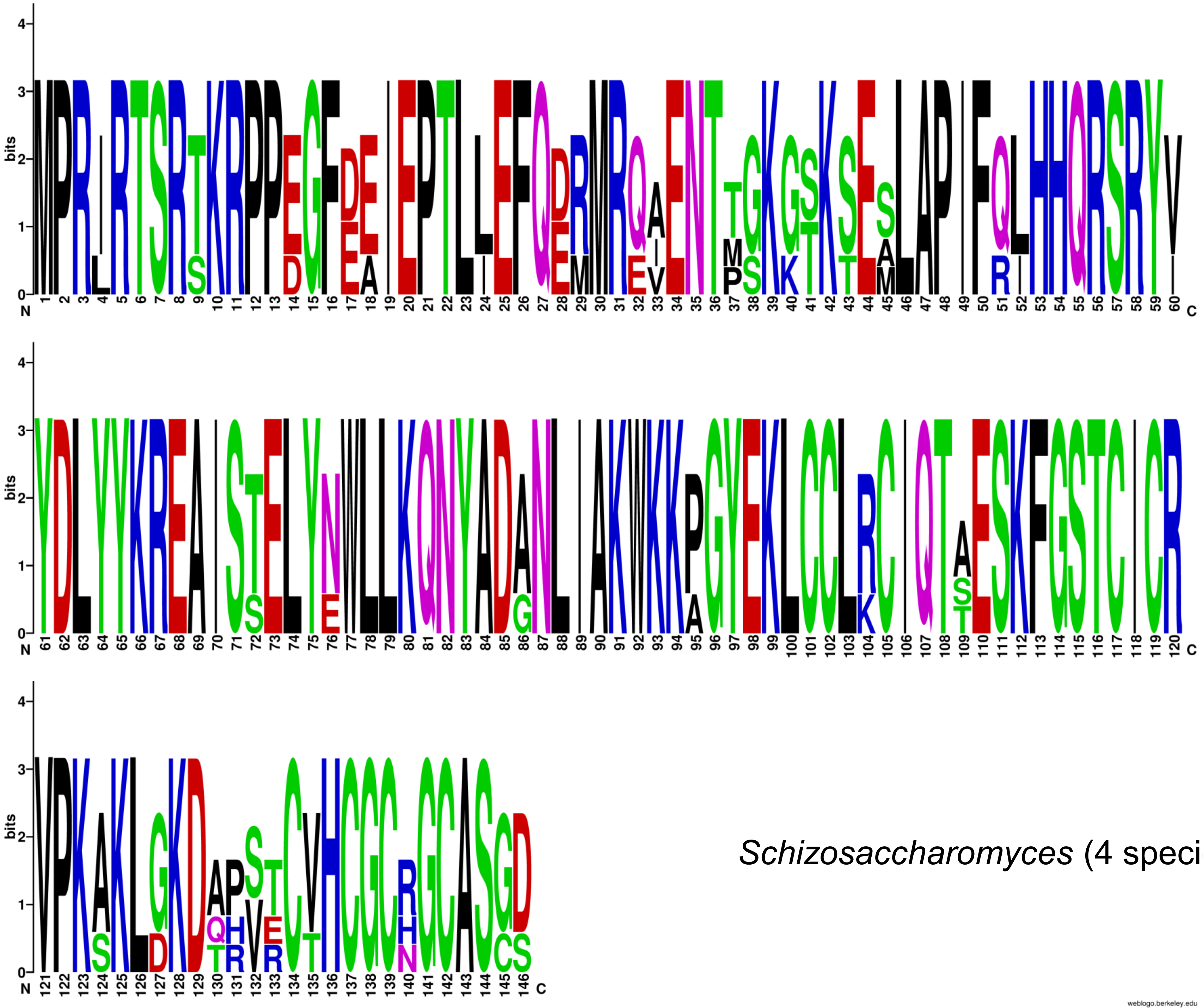

Schizosaccharomyces (4 species)

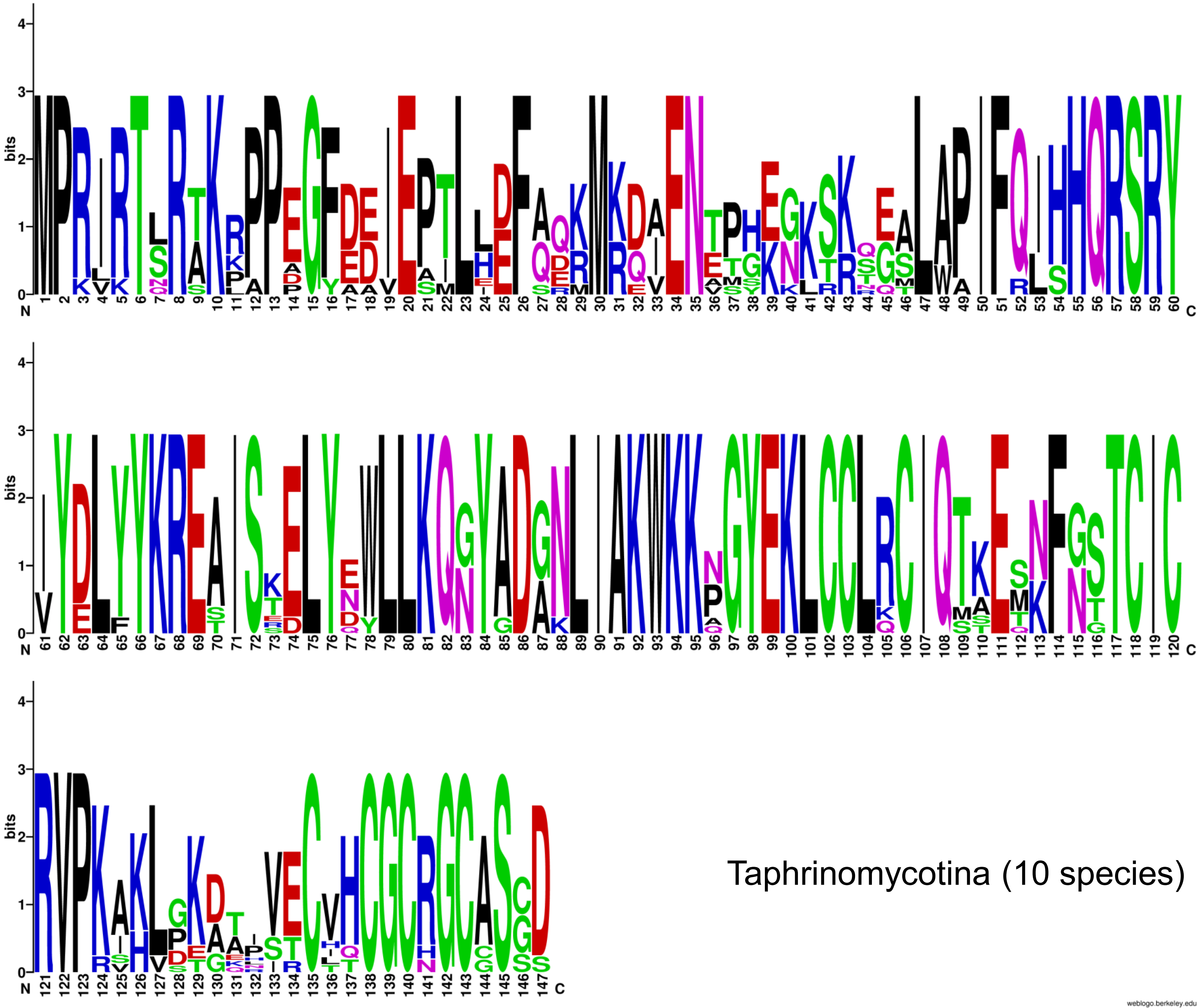

Taphrinomycotina (10 species)

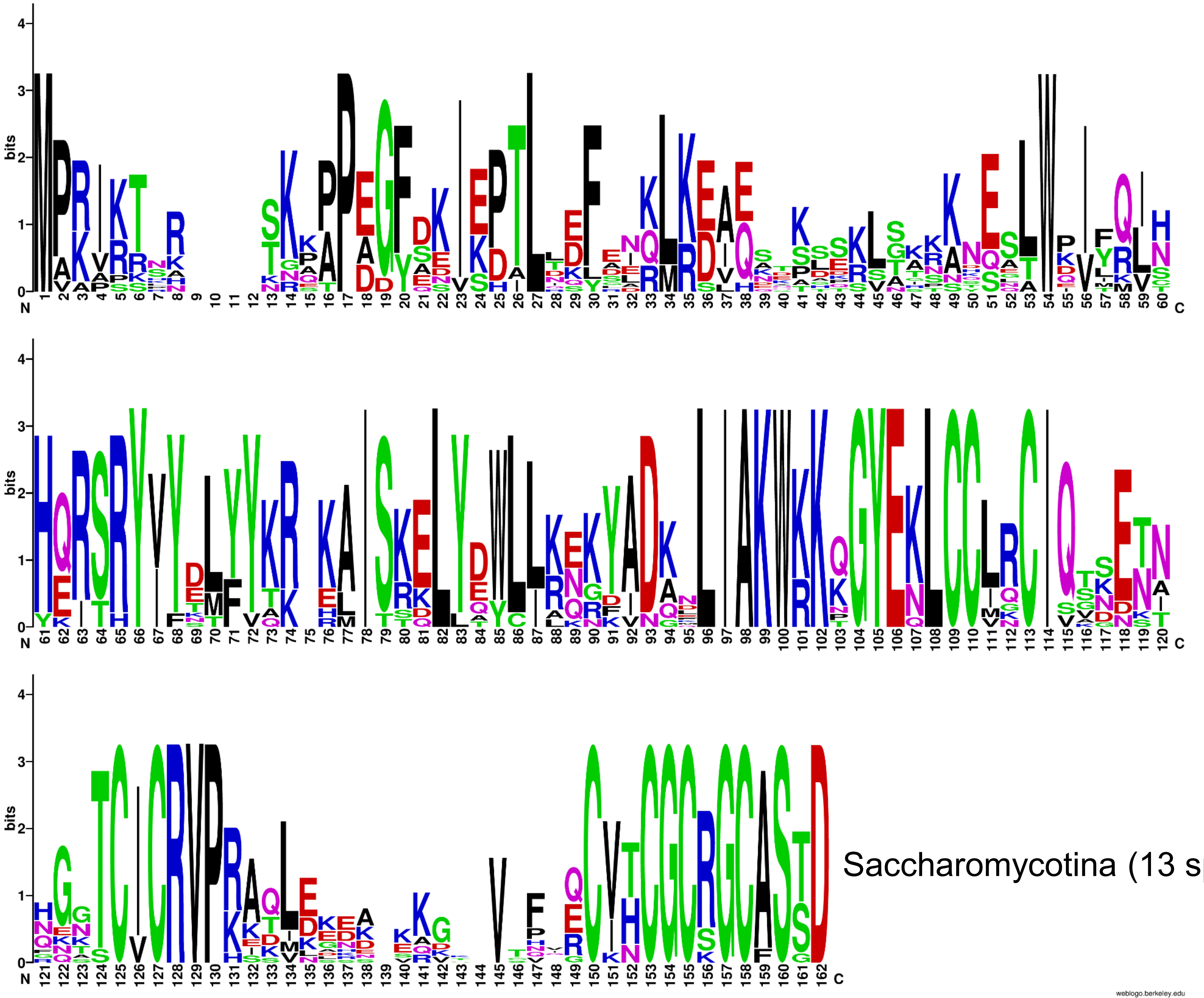

Saccharomycotina (13 species)

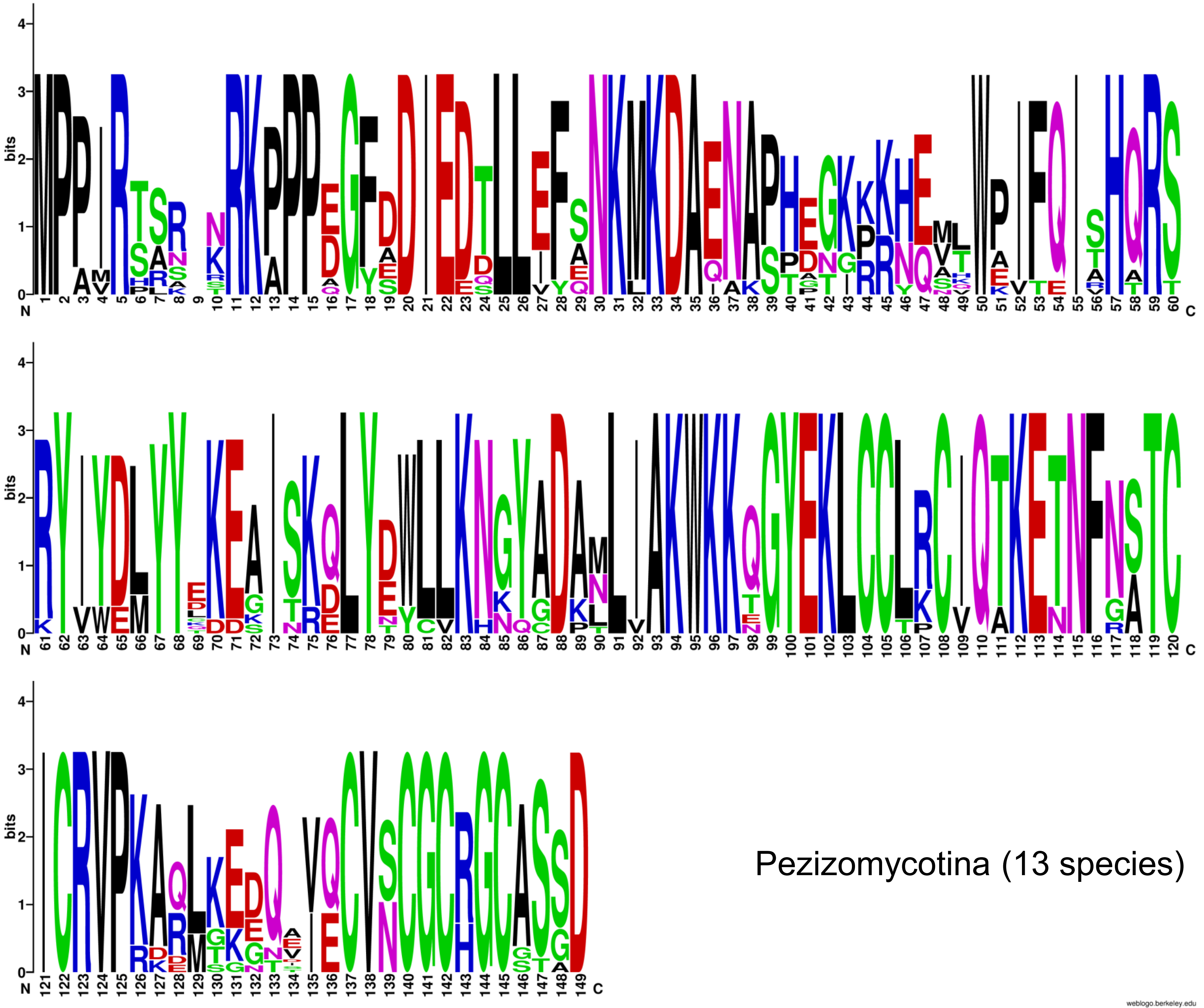

Pezizomycotina (13 species)

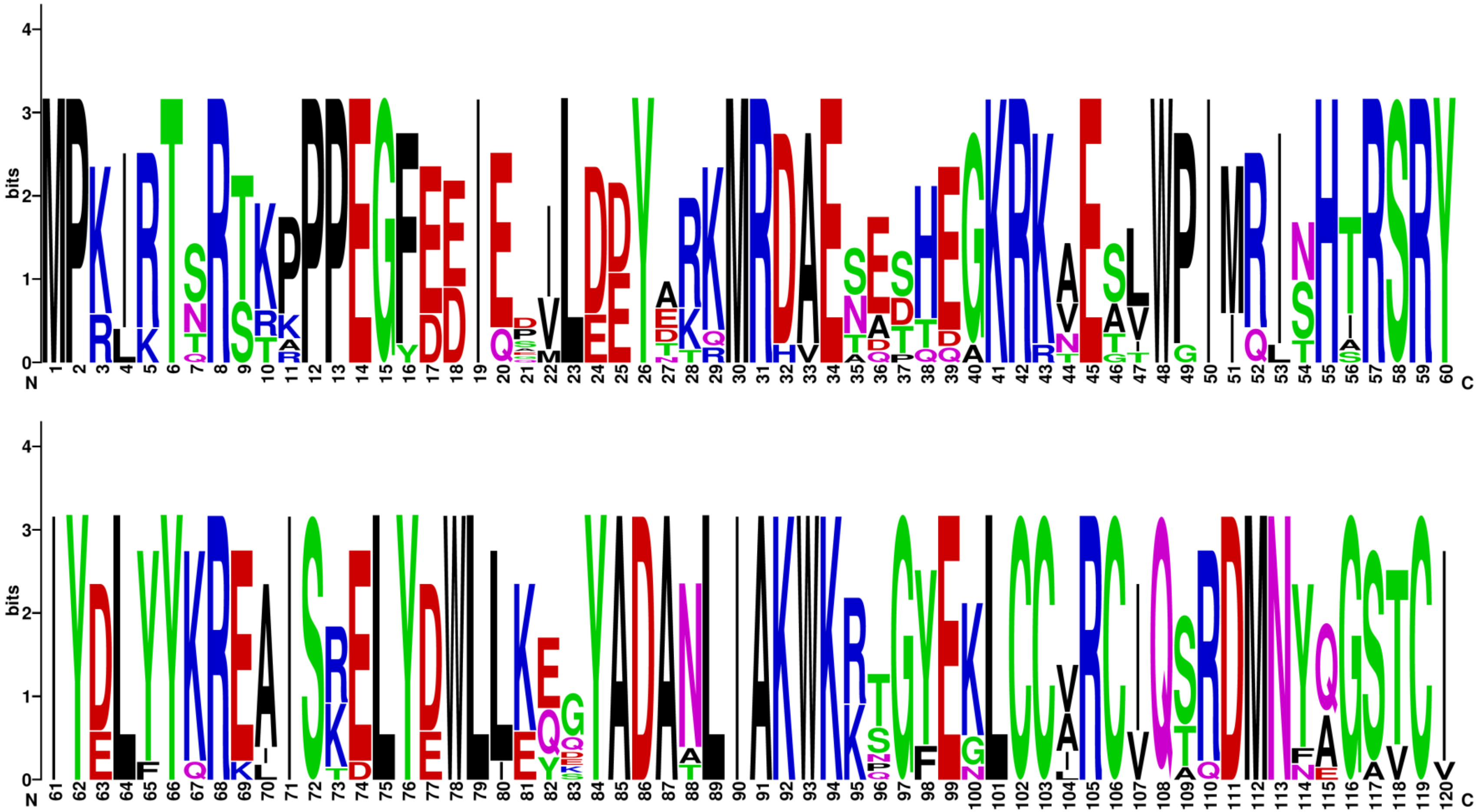

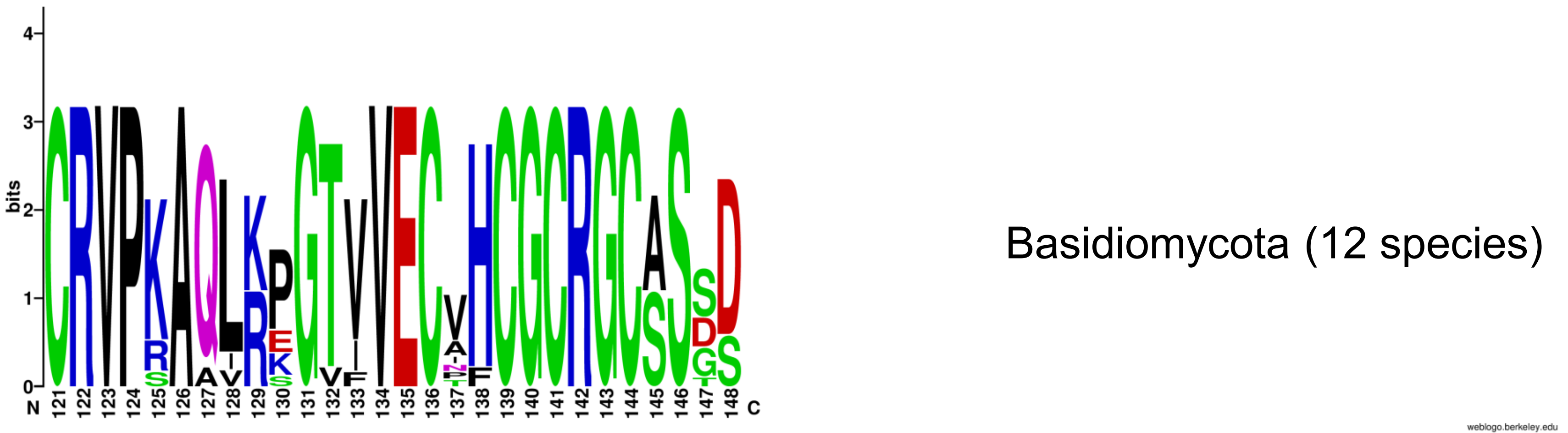

Basidiomycota (12 species)

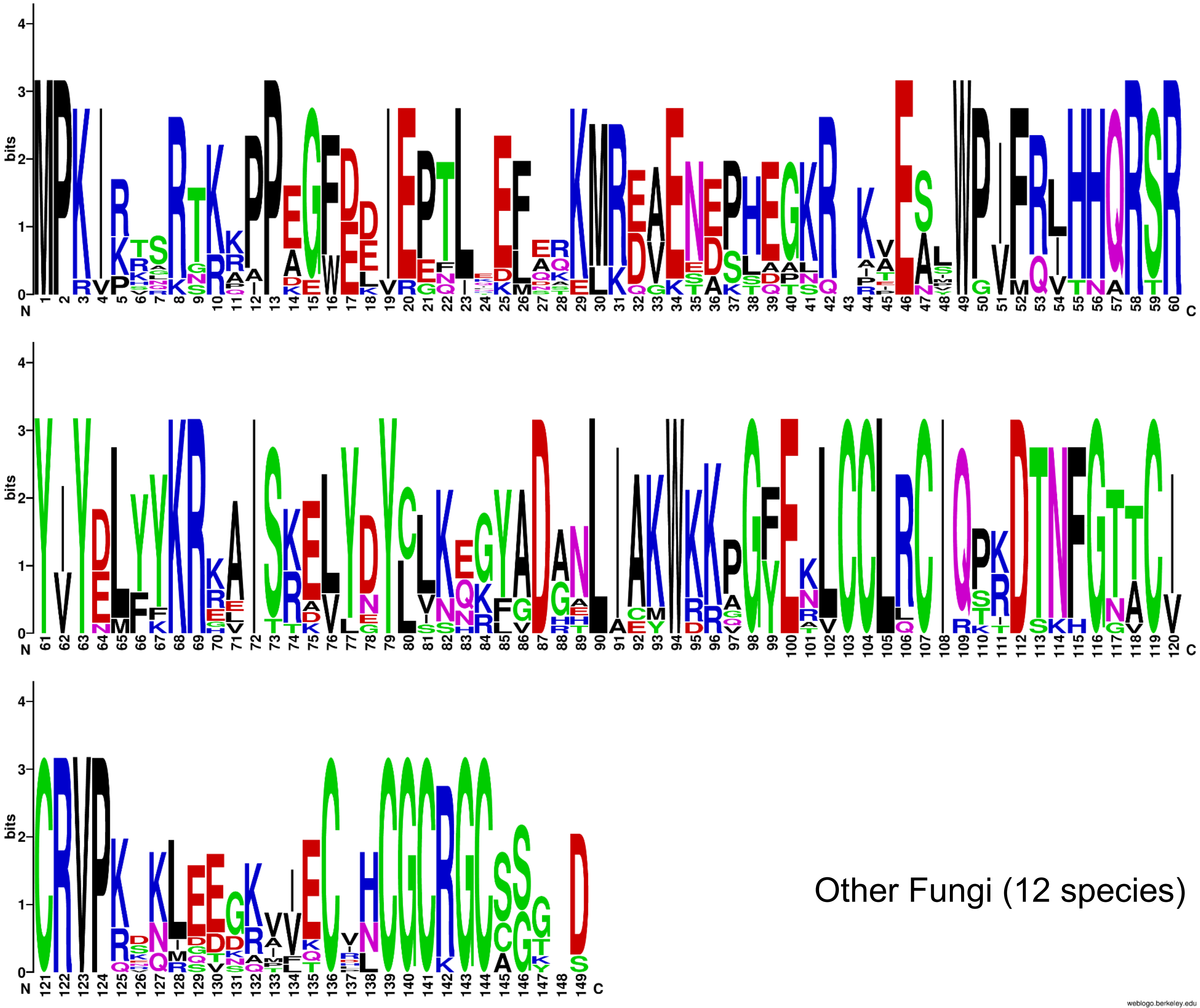

Other Fungi (12 species)

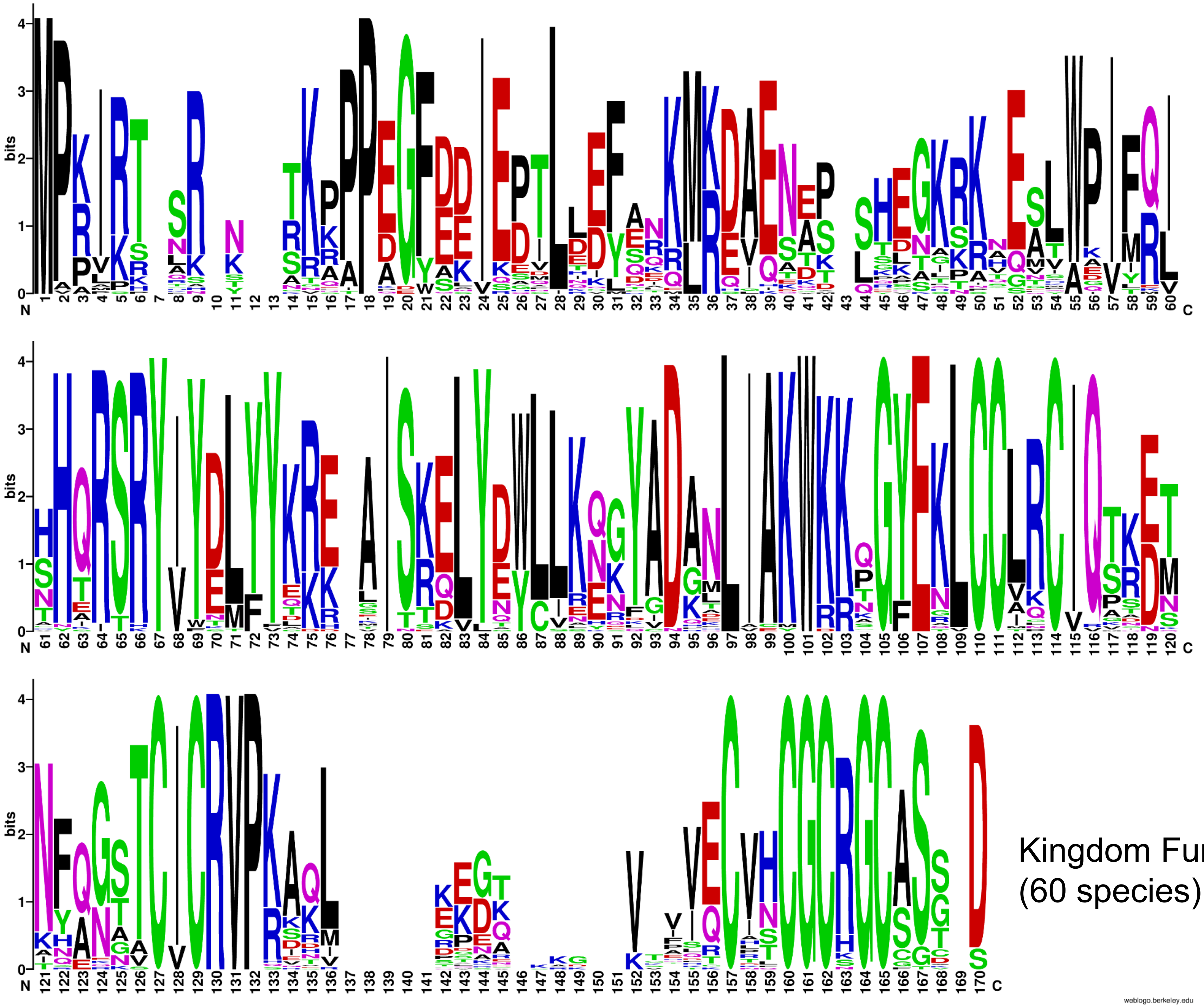

Kingdom Fungi
(60 species)

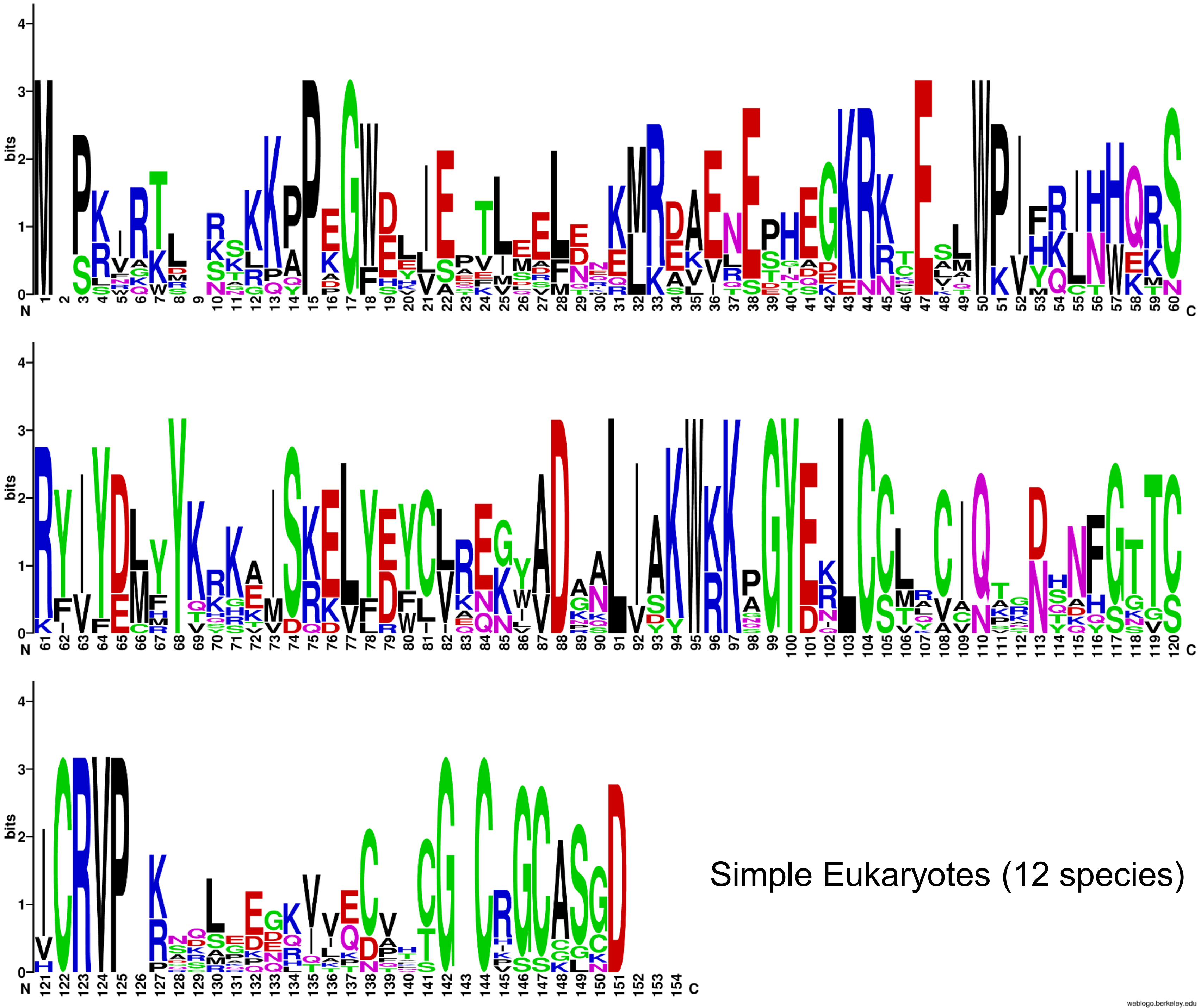
Simple Eukaryotes (12 species)
weblogo.berkeley.edu

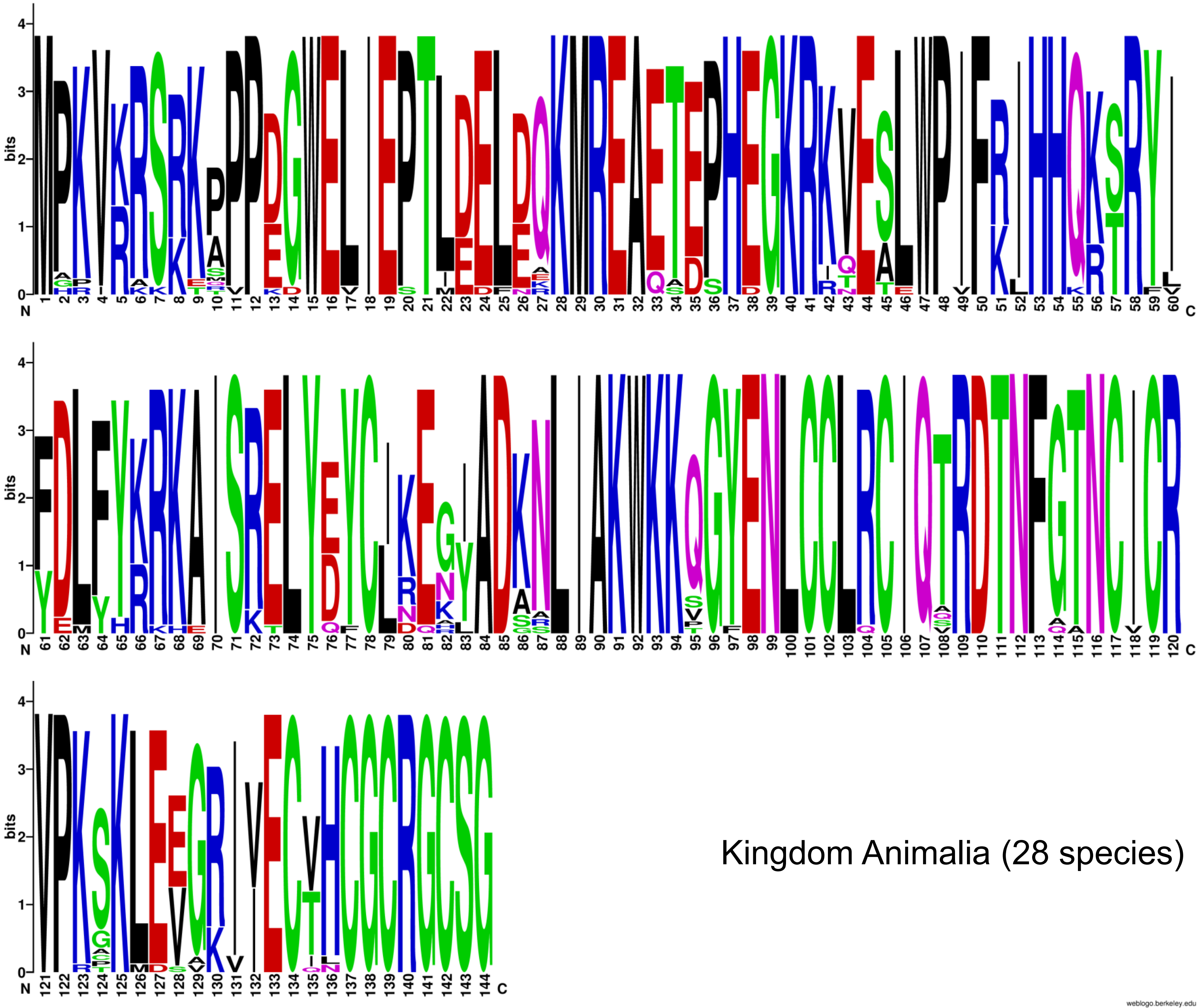

Kingdom Animalia (28 species)

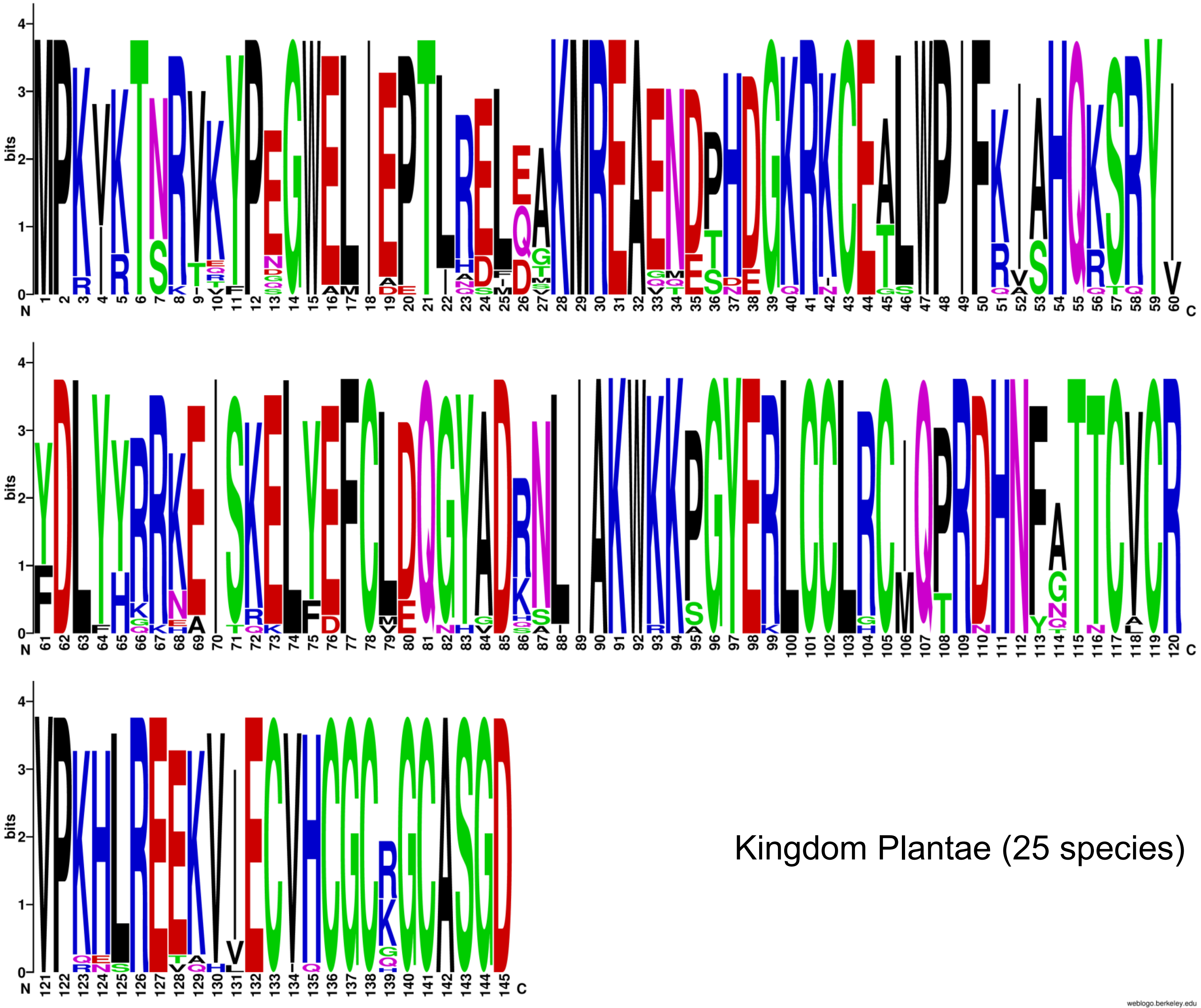

Kingdom Plantae (25 species)

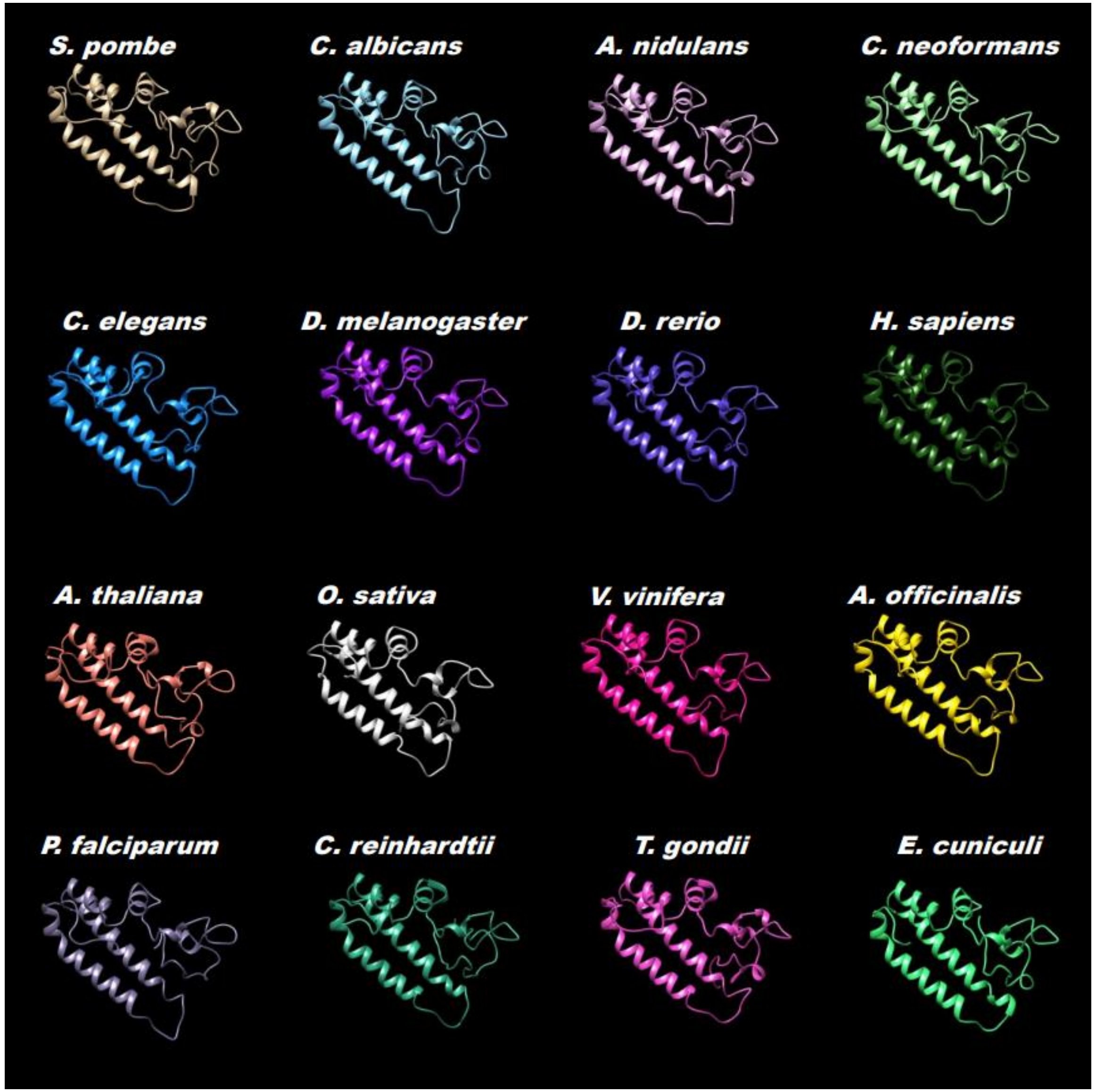

Figure S6. Protein structure prediction of several Cwf14 (BUD31) putative orthologs. As cryo-EM and MR templates exist, 99% of the residues could be modeled with >90% confidence. The depicted structures show that the *S. pombe* Cwf14 and its putative orthologous proteins exhibit almost identical protein structures. Structure predictions were made with the Phyre2 server and visualized with the UCSF Chimera software.

a

Alignment statistics for match #1

Score	Expect	Method	Identities	Positives	Gaps
126 bits(317)	5e-43	Compositional matrix adjust.	66/138(48%)	94/138(68%)	7/138(5%)

```
Query  14   DGFDEIEPTLIEFQDRMRQIE-NTMGKGTKTEMLAPIFQLHHQRSRYIYDLYYKREAIST  72
            +G+ +IEPTL +++++++   + N      +K    L   I+QL+++ +RY+YD Y   +   IS
Sbjct  15   EGYSKIEPTLTKYRNKLKSAQANPDPTKSKQSSLWIIYQLNYKITRYVYDTYVAKR-ISK  73

Query  73   ELYNWLLKQNYADGNLIAKWKKPGYEKLCCLRCIQTAESKFGSTCICRVPKSK-LDKDQ-  130
            ELY+WLL QN   + +LIAKWKKPGYEKLCC+ CI T   +   G TC+CRVPK+K L+KD
Sbjct  74   ELYDWLLLQNDINKDLIAKWKKPGYEKLCCINCIST-NTNGGGTCVCRVPKAKLLEKDPE  132

Query  131  --RVRCTHCGCNGCASCD  146
               + C   CGC GCAS D
Sbjct  133  KVNIECITCGCRGCASSD  150
```

Figure S7. Significant sequence identity was found between the *S. pombe* Cwf14 protein and human and *Candida* BUD31p (BLASTp analysis of protein sequences). ***Schizosaccharomyces pombe* Cwf14p (Query)-*Candida albicans* BUD31p (Sbjct)(a),** *Schizosaccharomyces pombe* Cwf14p (Query)-human BUD31p (Sbjct) (b).

b

Score	Expect	Method	Identities	Positives	Gaps
**181 bits(459)**	9e-65	Compositional matrix adjust.	86/145(59%)	111/145(76%)	2/145(1%)

```
Query  1    MPRLRTSRTKRPPDGFDEIEPTLIEFQDRMRQIENTMGKGT-KTEMLAPIFQLHHQRSRY  59
            MP+++ SR K PPDG++ IEPTL E   +MR+ E    +G  K E L PIF++HHQ++RY
Sbjct  1    MPKVKRSR-KAPPDGWELIEPTLDELDQKMREAETEPHEGKRKVESLWPIFRIHHQKTRY  59

Query  60   IYDLYYKREAISTELYNWLLKQNYADGNLIAKWKKPGYEKLCCLRCIQTAESKFGSTCIC  119
            I+DL+YKR+AIS ELY + +K+ YAD NLIAKWKK GYE LCCLRCIQT ++ FG+ CIC
Sbjct  60   IFDLFYKRKAISRELYEYCIKEGYADKNLIAKWKKQGYENLCCLRCIQTRDTNFGTNCIC  119

Query  120  RVPKSKLDKDQRVRCTHCGCNGCAS  144
            RVPKSKL+  + + CTHCGC GC+
Sbjct  120  RVPKSKLEVGRIIECTHCGCRGCSG  144
```

Figure S7. Significant sequence identity was found between the *S. pombe* Cwf14 protein and human and *Candida* BUD31p (BLASTp analysis of protein sequences). *Schizosaccharomyces pombe* Cwf14p (Query)-*Candida albicans* BUD31p (Sbjct)(a), ***Schizosaccharomyces pombe* Cwf14p (Query)-human BUD31p (Sbjct) (b).**
