# Supplementary material for: The BUD31 Homologous Gene in Schizosaccharomyces pombe Is Evolutionarily Conserved and Can Be Linked to Cellular Processes Regulated by the TOR Pathway
Source: Cells. 2025 Nov 5;14(21):1736. doi: 10.3390/cells14211736 (PMC12610034; doi:10.3390/cells14211736)
Supplement: Supplementary file 1 [file cells-14-01736-s001.zip › Supplemental tables 1,2,3,5,6,7.pdf]

Table S1. Strains used in this study.

Collection number	Genotype	Source
0-1 (L972)	wild-type h^-	Labstock
0-3 (L968)	wild-type h^{90}	Labstock
2-1201 (SA21)	wild-type h^+	Labstock
2-1199	*leu1-32* h^-	Labstock
2-1480 (SO9)	*cwf14*Δ::*kanMX6 ade6-M216* $ura4^-$*-D18 leu1-32* h^+	Bioneer M-3030H, V3-P08-12
2-1530	*cwf14Δ::kanMX6 leu1-32* h^{90}	this study
2-1532	*cwf14Δ::kanMX6 leu1-32* h^-	this study
2-1542	*cwf14*Δ::*kanMX6* h^-	this study
PP574	*dh1L<<lacO-*$ura4^+$ $his7^+$*<<Pdis1-GFP-lacI leu1 ura4-D18 CΔ::hyg.Br<<Padh15-CFP-atb2* h^+	[32]
P138	*leu1-32 ura4-D18 ade6-M210* h^-	[33]
15831 (B3 h^+)	*dh1L<<lacO-*$ura4^+$ *his7+<<Pdis1-GFP-lacI leu1 ura4-D18 ade6-M216* h^+	this study
15884 (B3 h^+ CEN1b)	*dh1L<<lacO-*$ura4^+$ *his7+<<CloneNat Pdis1-GFP-lacI leu1 ura4-D18* h^+	this study
15953 (B3 h^{90} CEN1b)	*dh1L<<lacO-*$ura4^+$ $his7^+$*<<CloneNat Pdis1-GFP-lacI leu1 ura4-D18* h^{90}	this study
16051 (B3 h^{90} CEN1b h2a-mCherry)	*leu1 ura4-D18 dh1L<<lacO-*$ura4^+$ $his7^+$*<<CloneNat Pdis1-GFP-lacI chr2 (2044894-2044963)::h2a-mCherry-Hyg1* (insertion of *h2a-mCherry-Hyg1* into intergenic region between *snoz30 and rpl8* close to *his7)* h^{90}	this study
16903 (B3 h^- CEN1b)	*dh1L<<lacO-*$ura4^+$ $his7^+$*<<CloneNat Pdis1-GFP-lacI leu1 ura4-D18 ade6* h^-	this study
2-1490 (SO9 h^- CEN1b)	*cwf14*Δ::*kanMX6*, *ade6 leu1* $ura4^-$*-D18 dh1L::lacOp-*$ura4^+$ *his7Δ::NatMX6 lacI-GFP* h^-	this study

Table S2. Primers used in this study.

Collection number of the primers	Name of the primers	Sequence 5'-3'	Restriction enzyme site
906	*S. pombe cwf14* Forward	GGG*CTCGAG*ATGCCTCGTCTTCGAACGTC	XhoI
907	*S. pombe cwf14* Reverse	TTT*CCCGGG*TTAATCACAGCTTGCACAAC	SmaI
910	Human *BUD31* (*hBUD31*) Forward	GGGGGG*CTCGAG*ATGCCTAAAGTCAAAAGAA	XhoI
911	Human *BUD31* (*hBUD31*) Reverse	TTT*CCCGGG*TCAGCCAGAGCAGCCACGAC	SmaI
912	*C. albicans BUD31* (*cBUD31*) Forward	GGG*CTCGAG*ATGCCAAAGATAAAAAAACC	XhoI
913	*C. albicans BUD31* (*cBUD31*) Reverse	AAA*CCCGGG*TTAATCACTAGAAGCACAGC	SmaI
527	KanMX6 Forw	AATGTCGGGCAATCAGGTGC	

Recognition sites of the restriction enzyme are in italics.

Table S3. Plasmids used in this study.

Collection number	Name of the plasmid	Source
26	pREP3x	[41]
27	pREP81	[41]
572	pJET1.2+*cBUD31*	this study
573	pJET1.2+*cwf14*	this study
574	pJET1.2+*hBUD31*	this study
575	pREP3X+*cBUD31*	this study
735	pREP3X+*cwf14*	this study
737	pREP3X+*hBUD31*	this study
420	pREP3x+*fhl1*	[70]
443	pREP81+*tor1*	[70]
442	pREP81+*tor2*	[70]

Table S5. Selected genes with significantly altered mRNA levels in the *cwf14* and, in *tor1*, *tor2*, or *fhl1* mutant strains.

Gene identifier	Gene name	Description	*cwf14* * log_2 value	*tor1* ** log_2 value	*tor2**** changes in mRNA level	*fhl1***** changes in mRNA level
SPBPB21E7.04c	*cmt2*	*O*-methyltransferase, human COMT catechol homolog 2	7.18	4.60		
SPBC19C7.04c		DUF2406 family conserved fungal protein	5.60	2.01	up	
SPBPB21E7.11		*Schizosaccharomyces pombe*- specific protein	5.20	1.90		
SPCC737.04		UPF0300 family protein 6	4.50	2.21	up	
SPAC977.02	*ftm2*	sub-telomeric 5Tm protein family Ftm2	4.10	3.53		
SPBPB8B6.02c		plasma membrane urea transmembrane transporter	3.73	5.68		
SPBPB2B2.08		conserved fungal protein	3.14	-2.51		down[m]
SPBC1348.03	*ftm6*	sub-telomeric 5Tm protein family Ftm6	2.77	6.30		
SPBPB2B2.06c	*efn1*	extracellular 5'-nucleotidase, human NT5E family	2.19	-2.51		
SPBC1348.12		DNA-binding transcription factor	1.92	2.03		down[m]
SPAC869.02c	*yhb1*	nitric oxide dioxygenase Yhb1	1.72	2.43		up[m]
SPBC1348.08c		cell surface glycoprotein, adhesion molecule	1.63	3.84		
SPAC186.05c	*gdt1*	Golgi calcium and manganese antiporter Gdt1	-3.29			down[cm]
SPAPB24D3.07c		*Schizosaccharomyces pombe*- specific protein	1.97			up[m]
SPAC869.01		hydrolase activity, implicated in cellular detoxification	3.98			up[m]
SPAC869.03c		plasma membrane urea transmembrane transporter	3.99			up[m]
SPAC869.04		formamidase-like protein, implicated in cellular detoxification	7.36			up[m]
SPAC869.05	*sul2*	plasma membrane sulfate transmembrane transporter Sul2	2.07			down[m]
SPAC2E1P3.05c	*cbm1*	fungal cellulose binding domain protein	-1.65		up	up[c]
SPCC663.08c		short chain dehydrogenase, unknown specificity	2.22			down[c]
SPCC794.04c		transmembrane transporter	3.03		up	
SPBC1683.12		carboxylic acid transmembrane transporter	1.99		up	
SPBC1683.06c	*urh1*	uridine ribohydrolase Urh1	3.02		up	
SPAC3G6.07		*Schizosaccharomyces*- specific protein	3.72		up	
SPAC3A11.10c		dipeptidyl peptidase, unknown specificity, implicated in glutathione metabolism	1.65		up	
SPAPJ691.02	*moh1*	yippee-like protein	2.33		up	
		**non-coding RNAs**				
SPNCRNA.1303			5.02	3.89		
SPNCRNA.1304			6.03	3.53		
SPNCRNA.1527			4.47	2.74		
SPNCRNA.944			-2.80	1.92		
SPNCRNA.1315			1.98	1.70		

up: upregulated; down: downregulated; c: grown in complex medium; m: grown in minimal medium. data obtained from:*[17] (RNA sequencing); **[73] (RNA sequencing); ***[65] (microarray); ****[70] (microarray).

Table S6. TOR pathway-associated genes showing intron retention.

**Ratios of exon-exon junction counts from RNA-seq *cwf14Δ*/WT***	**Associated common name**	**Associated systematic name**	**Function**	**Relation to the TOR pathway**
0,83	*gad8*	SPCC24B10.07	serine/threonine protein kinase (AGC family) Gad8	phosphorylated by Tor1 [64]
0,76	*tti2*	SPBC1604.17c	TTT co-chaperone complex subunit Tti2	affected by the mutation in *tor1* [30]
0,90	*tco89*	SPCC162.12	TORC1 subunit Tco89	TORC1 subunit [64]
0,00		SPAC1039.01	plasma membrane amino acid transmembrane transporter family, related to 7-keto 8-aminopelargonic acid transporter	Genes induced by loss of *tor2* ** [65]
0,29	*wtf21*	SPCC1739.15	wtf meiotic drive antidote-like Wtf21	**
0,75		SPCC569.03	tdk family toxin-antidote meiotic driver	Genes with altered mRNA levels in the *fhl1* mutant strain *** [70]
0,57		SPCC1620.06c	ribose-phosphate pyrophosphokinase	***
0,38		SPCC330.03c	cytochrome b5 reductase	***
0,00		SPAC1039.01	plasma membrane amino acid transmembrane transporter family, related to 7-keto 8-aminopelargonic acid transporter	***
0,57		SPCC1620.12c	GTPase activating protein	***
0,00		SPAC212.04c	S. pombe specific DUF999 family protein 1	***
0,00	*pfl7p*	SPBC359.04c	cell surface glycoprotein, flocculin Pfl7, DIPSY family	***
0,34	*mcp7*	SPAC13A11.03	meiosis specific coiled-coil protein Mcp7	***

*Data obtained from Kallgren *et al.*, 2014 [17]

Table S7. BUD31 homologous sequences of the various species.

>XP_020260311.1 protein BUD31 homolog 2 [Asparagus officinalis]

MPKIKTSRVKFPEGWELIEPTLRELEAKMREAENDPHDGKRKCEALWPIFQISHQKSRYIYDLYYRRKEISKELYEFCLDQGYADKN
LIAKWKKPGYERLCCLRCIQPRDHNFGTTCVCRVPKHLREEKVIECVHCGCRGCASGD

>XP_012075869.1 protein BUD31 homolog 1 [Jatropha curcas]

MPKVRTNRVKYPDGWELIEPTLRELDAKMREAENDPHDGKRKCEALWPIFKIAHQRSRYIFDLYYKRNEISKELYEFCLEQGYGDQN
LIAKWKKPGYERLCCLRCIQSRDHNYGTTCVCRVPKNLREEKVVECVHCGCGGCASGD

>NP_001130110.1 uncharacterized protein LOC100191203 [Zea mays]

MPKIKTSRVKYPEGWELIEPTIRELDAKMREAENDPHDGKRKCEALWPIFRISHQRSRYIYDLYYRRKEISRELYEFCLDQSYADRN
LIAKWKKPGYERLCCLRCIQTRDHNFATTCVCRVPKHLREEQVIECVHCGCRGCASGD

>NP_001148940.1 G10-like protein [Zea mays]

MPKIKTSRVKYPEGWELIEPTIRELDAKMREAENDPHDGKRKCEALWPIFRISHQRSRYIYDLYYRRKEISQELYEFCLDQGYADRN
LIAKWKKPGYERLCCLRCIQTRDHNFATTCVCRVPKHLREEQVIECVHCGCRGCASGD

>XP_017255506.1 PREDICTED: protein BUD31 homolog 1-like [Daucus carota subsp. sativus]

MPKVRTNRVKVPEGFELIEPTLLELQAKMREAENDTHDGKRKCETMWPIFKIAHQKSRYVFDLYHRRKEISKELYEFCLEQGYADRN
LIAKWKKPGYERLCCLRCMQPRDHNFQTTCVCRVPKHLREEKVIECVHCGCNGCASGD

>XP_018480865.1 PREDICTED: protein BUD31 homolog 1-like isoform X2 [Raphanus sativus]

MPKVKTNRIKYPEGWELIEPTLREFEAKMREAEVDTHDGKRKCEALWPIFKLSHQRSRYVYDLYYRREEISKELYEFCLDQNYADRN
LIAKWKKSGYERLCCLRCIQPRDHNYGTTCVCRVPKHLREEKVVECVHCGCQGCASGD

>XP_006471391.1 PREDICTED: protein BUD31 homolog 2 [Citrus sinensis]

MPKVRTNRTKYPDGWELIAPTLREMEAKMREAENDPHDGKRKCETLWPIFKIAHQRSQYIFDLYYKRNEISKELYEFCLDQGYGDSN
LIAKWKKPGYERLCCLRCIQPRDHNFGTTCVCRVPKHLREEKHVECVHCGCKGCASGD

>XP_003568344.1 PREDICTED: protein BUD31 homolog 2 [Brachypodium distachyon]

MPKIKTSRVKYPEGWELIEPTLRDLEAKMREAENDTHDGKRKCEALWPIFRISHQKSRYIYDLYYRRKEISKELYEFCLDQGYADKN
LIAKWKKPGYERLCCLRCIQTRDHNFATTCVCRVPKHLREEKVIECVHCGCKGCASGD

>OMO57678.1 G10 protein [Corchorus capsularis] >OMO89080.1 G10 protein [Corchorus olitorius]

MPKVRTNRIKYPDGWELIEPTLRELDAKMREAENDPHDGKRKCEALWPIFKIAHQRSRYIFDLYYKRNEISKELYEFCLEQGYGDSN
LIAKWKKPGYERLCCLRCIQPRDHNFGTTCVCRVPQHLREEKVVECVHCGCRGCASGD

>XP_017238894.1 PREDICTED: protein BUD31 homolog 1 [Daucus carota subsp. sativus]

MPKVRTNRVKVPEGFELIEPTLLELQAKMREAENDTHDGKRKCETLWPIFKIAHQKSRYVYDLYHRRKEISKELYEFCLDQGYADRN
LIAKWKKPGYERLCCSRCMQPRDHNFQTTCVCRVPKHLREEKVIECVHCGCNGCASGD

>XP_006654481.1 PREDICTED: protein BUD31 homolog 2 [Oryza brachyantha]

MPKIKTSRVKYPEGWELIEPTLRDLEAKMREAENDSHDGKRKCEALWPIFRISHQKSRYIYDLYYRRKEISKELYEFCLDQGYADKN
LIAKWKKPGYERLCCLRCIQTRDHNFATTCVCRVPKHLREEKVIECVHCGCRGCASGD

>XP_003564654.1 PREDICTED: protein BUD31 homolog 1 [Brachypodium distachyon]

MPKIKTSRVKYPEGWELIEPTIRDLDAKMREAENDTHDGKRKCEALWPIFRISHQRSRYIYDLYYRRKEISKDLYEFCLDQGYADRN
LIAKWKKPGYERLCCLRCIQTRDHNFATTCVCRVPKHLREEQVIECVHCGCKGCASGD

>XP_018480864.1 PREDICTED: protein BUD31 homolog 1-like isoform X1 [Raphanus sativus]

MIYQKTHIKPNPNLKRGFNDDAGSYLSSSSSNSLVLRQLVSPKKKMPKVKTNRIKYPEGWELIEPTLREFEAKMREAEVDTHDGKRK
CEALWPIFKLSHQRSRYVYDLYYRREEISKELYEFCLDQNYADRNLIAKWKKSGYERLCCLRCIQPRDHNYGTTCVCRVPKHLREEK
VVECVHCGCQGCASGD

>XP_004139476.1 PREDICTED: protein BUD31 homolog 1 [Cucumis sativus]

MPKVKTSKIKYPNGWELIEPTLRELDAKMREAENDPQDGKRKCEALWPIFKISHQRSRYIFDLFYKRSEISRELYEFCLEQGYADAN
LIAKWKKPGYERLCCLRCIQPRDHNFGTTCVCRVPKHLREEKVVECVHCGCRGCASGD

>XP_018447829.1 PREDICTED: protein BUD31 homolog 1-like isoform X2 [Raphanus sativus]

MPKIKTNRVKYPEGWELIEPTLLEIEAKMREAEIDTHDGKRKCEALWPIFKLSHQRSRYVYDLYYRREEISKDLYEFCLDQNYADRN
LIAKWKKSGYERLCCLRCIQPRDHNYGTTCVCRVPKHLREEKVVECVHCGCQGCASGD

>XP_006645047.1 PREDICTED: protein BUD31 homolog 1 [Oryza brachyantha]

MPKIKTSRVKYPEGWELIEPTIRDLDAKMREAENDTHDGKRKCEALWPIFRISHQRSRYIYDLYYRRKEISKELYEFCLDQGYADRN
LIAKWKKPGYERLCCLRCIQTRDHNFATTCVCRVPKHLREEKVIECVHCGCRGCASGD

>XP_020149391.1 protein BUD31 homolog 1 [Aegilops tauschii subsp. tauschii

MPKIKTSRVEYPEGWVLIEPTIRELDAKMREAENDTHDGKRKCEALWPIFRISHQRSRYIYDLFYKRKEISKELYEFCLDQGYADRN
LIAKWKKPGYERLCCLRCIQTRDHNFATTCVCRVPKHLREEQVIECVHCGCKGCASGD

>XP_004961818.1 PREDICTED: protein BUD31 homolog 2 [Setaria italica]

MPKIKTSRVKYPEGWELIEPTLRDLEAKMREAENDPHDGKRKCEALWPIFRISHQKSRYIYDLYYRRKEISKELYEFCLDQGYADRN
LIAKWKKPGYERLCCLRCIQTRDHNFATTCVCRVPKHLREEKVIECVHCGCKGCASGD

>XP_021303684.1 protein BUD31 homolog 2 [Sorghum bicolor]

MPKIKTSRVKYPEGWELIEPTLRDLEAKMREAENDPHDGKRKCEALWPIFRISHQKSRYIYDLYYRRKEISKELYEFCLDQGYADRN
LIAKWKKPGYERLCCLRCIQTRDHNFATTCVCRVPKHLREEKVIECVHCGCRGCASGD

>XP_006424308.1 hypothetical protein CICLE_v10029519mg [Citrus clementina]

MPKVRTNRTKYPDGWELIAPTLREMEAKMREAENDPHDGKRKCETLWPIFKIAHQRSQYIFELYYKRNEISKELYEFCLDQGYGDSN
LIAKWKKPGYERLCCLRCIQPRDHNFGTTCVCRVPKHLREEKHVECVHCGCKGCASGD

>BAJ96105.1 predicted protein [Hordeum vulgare subsp. vulgare]

MPKIKTSRVEYPEGWALIEPTIRELDAKMREAENDTHDGKRKCEALWPIFRISHQRSRYIYDLFYRRKEISKELYEFCLDQGYADRN
LIAKWKKPGYERLCCLRCIQTRDHNFATTCVCRVPKHLREEQVIECVHCGCKGCASGD

>XP_010049717.1 PREDICTED: protein BUD31 homolog 2 [Eucalyptus grandis]

MPKVKTNRVKYPEGWELIEPTLRELQAKMREAENDPHDGKRKCEALWPIFKIAHQKSRYIYDLYYRRKEISKELYEFCLDQGYADKN
LIAKWKKPGYERLCCLRCIQPRDHNFATTCVCRVPKHLREEKVIECVHCGCRGCASGD

>XP_004970642.1 PREDICTED: protein BUD31 homolog 1-like [Setaria italica]
MPKIKTSRVSYPEGWELIEPTIRELDAKMREAENDPHDGKRKCEALWPIFRISHQRSRYIYDLYYRRKEISQKLYEFCLDQGYADRN
LIAKWKKPGYERLCCLRCIQTRDHNFATTCVCRVPKHLREEQVIECVHCGCKGCASGD

>XP_020691053.1 protein BUD31 homolog 2 isoform X2 [Dendrobium catenatum]

MPKVKTSRIKYPEGWELIEPTFRELEGKMREAENDPHDGKRKCEALWPIFKIAHQKSRYVYDLYYRRKEISKDLYEFCLDQGYADSN
LIAKWKKPGYERLCCLRCIQPRDHNFGTTCVCRVPKHLREEKVIECVHCGCGGCASGD

>XP_008783991.1 PREDICTED: protein BUD31 homolog 2 [Phoenix dactylifera]

MPKIKTSRVKYPEGWELIEPTLRDLEAKMREAENDPHDGKRKCEALWPIFKIAHQKSRYIYDLYYRRKEISKELYEFCLDQGYADRN
LIAKWKKPGYERLCCLRCMQTRDHNFATTCVCRVPKHLREEKVIECVHCGCRGCASGD

>XP_015637596.1 PREDICTED: protein BUD31 homolog 2 [Oryza sativa Japonica Group]
MPKIKTSRVKYPEGWELIEPTLRDLEAKMREAENDPHDGKRKCEALWPIFRISHQKSRYIYDLYYRRKEISKELYEFCLDQGHADKN
LIAKWKKPGYERLCCLRCIQTRDHNFATTCVCRVPKHLREEKVIECVHCGCRGCASGD

>XP_020185022.1 protein BUD31 homolog 2 [Aegilops tauschii subsp. tauschii

MPKIKTSRVKYPEGWELIEPTLRDLEAKMREAENDTHDGKRKCEALWPIFRISHQKSRYIYDLYYRRKEIKRELYEFCLDQGYADKN
LIAKWKKPGYERLCCLRCIQTRDHNFATTCVCRVPKHLREETVIECVHCGCKGCASGD

>XP_020108362.1 protein BUD31 homolog 2 [Ananas comosus]

MPKIKTSRVKYPEGWELIEPTLRELEAKMREAENDPHDGKRKCEALWPIFRIAHQKSRYIYDLYHRRKEISKELYEFCLDQGYADRN LIAKWKKPGYERLCCLRCMQTRDHNFATTCVCRVPKHLREEKVIECVHCGCGGCASGD

>XP_010912251.1 PREDICTED: protein BUD31 homolog 2 [Elaeis guineensis]

MPKIKTSRVKYPEGWELIEPTLRELEAKMREAENDPHDGKRKCEALWPIFKIAHQKSRYIYDLYYRRKEISKELYEFCLDQGHADRN LIAKWKKPGYERLCCLRCMQTRDHNFATTCVCRVPKHLREEKVIECVHCGCRGCASGD

>XP_006663819.1 PREDICTED: protein BUD31 homolog 3 [Oryza brachyantha

MPKIKTSGVKYPDGWELIEPTLSELQSKMREAENDPHDGKRKCEALWPIFKINHQRSRYLYDLYYNRKEISQELYEFCLDQGHADRN LIAKWKKQGYERLCCLRCIQTRDHNFATTCVCRVPRHLREEQVIECVHCGCKGCASGD

>OWM64868.1 hypothetical protein CDL15_Pgr028585 [Punica granatum]

MPKVKTSRVRYPEGWELIEPTLRELQAKMREAENDPHDGKRKCEALWPIFKIEHQKSRYIFDLYHRRKEISKELYEFCLDQRYADKN LIAKWKKPGYERLCCLRCIQPRDHNFGTTCACRVPKHLREEKVIECVHCGCRGCASGD

>KCW89225.1 hypothetical protein EUGRSUZ_A01529 [Eucalyptus grandis]

MCLVKENPYYGNKQFKQRQKTPQKNHSVTSYPPLSLDLPKPLFSPAKQPILSPLTVSSRSPSGRKSLSRQGISMPKVKTNRVKYPEG WELIEPTLRELQAKMREAENDPHDGKRKCEALWPIFKIAHQKSRYIYDLYYRRKEISKELYEFCLDQGYADKNLIAKWKKPGYERLC CLRCIQPRDHNFATTCVCRVPKHLREEKVIECVHCGCRGCASGD

>XP_009411965.1 PREDICTED: protein BUD31 homolog 2 [Musa acuminata subsp. malaccensis]

MPKIKTSRVKYPEGWELIEPTLRELEAKMREAENDPHDGKRKCEALWPIFRIAHQKSRYIYDLYHRRKEISKELYEFCLDQGYADRN LIAKWKKPGYERLCCLRCMQTRDHNFATTCVCRVPKHLREEKVIECVHCGCRGCASGD

>XP_020581573.1 protein BUD31 homolog 2 [Phalaenopsis equestris

MPKVKTNRVKYPEGWELIEPTLRELEGKMREAENDPHDGKRKCEALWPIFKISHQKSRYIYDLYYRRKEISKDLYEFCLDQGYADRN LVAKWKKPGYERLCCLRCIQPRDHNFGTTCVCRVPKHLREEKVIECVHCGCGGCASGD

>OAY67731.1 Protein BUD 2 [Ananas comosus]

MPKIKTSRVKYPEGWELIEPTLHELEAKMREAENDPHDGKRKCEALWPIFRIAHQKSRYIYDLYHRRKEISKELYEFCLDQGYADRN LIAKWKKPGYERLCCLRCMQTRDHNFTTTCVCRVPKHLREEKVIECVHCGCGGCASGD

>XP_008461749.1 PREDICTED: protein BUD31 homolog 1-like [Cucumis melo]

MPKVKTSKIKYPNGWELIEPTLRELDAKMREAENDPQDGKRKCEALWPIFKISHQRSRYIFDLFHKRSEISRELYEFCLEQGYADAN IIAKWKKPGYERLCCLRCIQPRDHNFGTTCVCRVPKHLREEKVVECVHCGCRGCASGD

>XP_003578906.1 PREDICTED: protein BUD31 homolog 3 [Brachypodium distachyon]

MPKVKTSGVKYPDGWEVIEPTLSELHSKMREAENDPHDGKRKCEALWPIFKINHQRSRYLYDLYYNRKEISRELYEFCLDQGHADRN LIAKWKKPGYERLCCLHCIQTRDHNFATTCACRVPKHLREEQVIECVHCGCKGCASGD

>XP_020106729.1 protein BUD31 homolog 2-like [Ananas comosus]

MPKIKTSRVKYPEGWELIEPTLRELEAKMREAENDPHDGKRKCEALWPIFRIAHQKSRYIYDLYHRRKEISKELYEFCLDQGYADRN LIAKWKKPGYERLCCLRCMQTRDHNFTTTCVCRVPKHLREEKVIECVHCGCGGCASGD

>XP_015620231.1 PREDICTED: protein BUD31 homolog 3 [Oryza sativa Japonica Group]

MPKIKTSGVKYPDGWELIEPTLSELHSKMREAENDPHDGRRKCEALWPIFKINHQRSRYLYDLYYNRKEISQELYEFCLDQGHADRN LIAKWKKQGYERLCCLRCIQTRDHNFATTCVCRVPKHLREEQVIECVHCGCKGCASGD

>XP_021627390.1 protein BUD31 homolog 1-like isoform X2 [Manihot esculenta] >XP_021627392.1 protein BUD31 homolog 1-like isoform X2 [Manihot esculenta] >XP_021627393.1 protein BUD31 homolog 1-like isoform X2 [Manihot esculenta] >OAY38170.1 hypothetical protein MANES_11G159100 [Manihot esculenta] >OAY38171.1 hypothetical protein MANES_11G159100 [Manihot esculenta]

MPKVKTNRIKYPDGWELIEPTLRELDAKMREAENDSHDGKRKCETLWPIFRIAHQRSRYIFDIYYKTNEISKELYEFCLE

QGYGDRNLIAKWKKPGYERLCCLRCIQPRDHNFGTTCVCRVPKHLREEKVVECVHCGCKGCASGD

>XP_021627389.1 protein BUD31 homolog 1-like isoform X1 [Manihot esculenta]

MEKGQFFELGRTSRYLSFTEYAEMPKVKTNRIKYPDGWELIEPTLRELDAKMREAENDSHDGKRKCETLWPIFRIAHQRS

RYIFDIYYKTNEISKELYEFCLEQGYGDRNLIAKWKKPGYERLCCLRCIQPRDHNFGTTCVCRVPKHLREEKVVECVHCG

CKGCASGD

>XP_013458166.1 cell cycle control protein, G10 family protein [Medicago truncatula] >KEH32197.1 cell cycle control protein, G10 family protein [Medicago truncatula]

MPKVKTSRVKYPEGWELIEPTLRELQGKMREAENDPHDGKRKCETLWPIFKIAHQKSRYVFELYHKRKEISKELYEFCLD

QGYADRNLIAKWKKPGYERLCCLRCIQPRDHNFATTCVCRVPKELREEKVIECVHCGCKGCASGD

>XP_001756359.1 predicted protein [Physcomitrella patens] >EDQ78755.1 predicted protein [Physcomitrella patens]

MPKVRTNRTVYPEGWELIEPTLRELETKMREAENETHEGKRKCEALWPIFKISHQKSRYIYDLFYRRKAISRALFDFCLE

QGHADKNLIAKWKKSGYERLCCLRCIQPRDHNFGTTCVCRVPKHLREEKVIECVHCGCHGCASGD

>XP_020248741.1 protein BUD31 homolog 2-like [Asparagus officinalis] >ONK57167.1 uncharacterized protein A4U43_C10F17290 [Asparagus officinalis]

MPKIKTSRVKYPEGWELIEPTLRELEAKMREAENDPHDGKRKCEALWPIFRISHQKSRYIFDLYYRRKEISKELYEFCLD

QGYADRNLIAKWKKSGYERLCCLRCIQPRDHNFGTTCVCRVPKHLREEKVIECVHCGCRGCASGD

>EEE52776.1 hypothetical protein OsJ_35232 [Oryza sativa Japonica Group]

MWRSGSLQRGGGGSGRRMPAKGPLASLAAEGASNPVGRRSIMPKIKTSGVKYPDGWELIEPTLSELHSKMREAENDPHDG

RRKCEALWPIFKINHQRSRYLYDLYYNRKEISQELYEFCLDQGHADRNLIAKWKKQGYERLCCLRCIQTRDHNFATTCVC

RVPKHLREEQVIECVHCGCKGCASGD

>XP_021658818.1 protein BUD31 homolog 1-like [Hevea brasiliensis] >XP_021658819.1 protein BUD31 homolog 1-like [Hevea brasiliensis] >XP_021658820.1 protein BUD31 homolog 1-like [Hevea brasiliensis] >XP_021658821.1 protein BUD31 homolog 1-like [Hevea brasiliensis]

MPKVKTNRIKYPDGWELIEPTLRELDAKMREAENDPHDGKRKCETLWPIFRIAHQRSRYIFDIYYKTNEISKELYEFCLE

QGYGDRNLIAKWKKPGYERLCCLRCIQPRDHNFGTTCVCRVPKHLREEKVVECVHCGCRGCASGD

>XP_004504370.1 PREDICTED: protein BUD31 homolog 2 [Cicer arietinum]

MPKVKTSRVKYPEGWELIEPTLRELQAKMREAENDPHDGKRKCETLWPIFKIAHQKSRYVFDLYHRRKEISKELYEFCLD

QGYADRNLIAKWKKPGYERLCCLRCMQPRDHNFATTCVCRVPKQLREEKVIECVHCGCKGCASGD

>XP_018447828.1 PREDICTED: protein BUD31 homolog 2-like isoform X1 [Raphanus sativus]

MPKIKTNRVKYPEGWELIEPTLLEIEAKMREAEIDTHDGKRKCEALWPIFKLSHQRSRYVYDLCYRREEISKELYEFCLD

QNHADRNLIAKWKKSGYERLCCLRCIQPRDHNYGTTCVCRVPKHLREEKVVECVHCGCQGCASGD

>XP_004508375.1 PREDICTED: protein BUD31 homolog 2-like [Cicer arietinum]

MPKVKTSRVQYPEGWELIEPTLRELQAKMREAENDPHDGKRKCETLWPIFKIAHQKSRYVFDLYHRRKEISKELYEFCLD

QGYADRNLIAKWKKPGYERLCCLRCIQPRDHNFATTCVCRVPKQLREEKVIECVHCGCKGCASGD

>XP_009137165.1 PREDICTED: protein BUD31 homolog 1-like [Brassica rapa] >CDY58485.1 BnaA03g58540D [Brassica napus]

MPKVKTNRVKYPEGWELIEPTLRELDAKMREAEMDEHDGKRKCEALWPIFKLSHQRSRYVYDLYYRREEISKELYEFCLD

QGYADRNLIAKWKKSGYERLCCLRCIQPRDHNYGTTCVCRVPKHLREEKVVECVHCGCQGCASGD

>XP_006840386.1 protein BUD31 homolog 1 isoform X2 [Amborella trichopoda] >ERN02061.1 hypothetical protein AMTR_s00045p00137520 [Amborella trichopoda]

MPKIKTNRVQYPGGWELIEPTLRELEAKMREAENDTHDGKRKCEALWPIFRIAHQKSRYIYDLYYRRKEISKELYEFCLE

QGYADRNLIAKWKKPGYERLCCLRCIQPRDHNFNTTCVCRVPKHLREEKVIECVHCGCRGCASGD

>GAU39510.1 hypothetical protein TSUD_68740 [Trifolium subterraneum]

MPKVKTSRVKYPEGWELIEPTLRELQGKMREAENDPHDGKRKCETLWPIFKIAHQKSRYVFDLYHRRKEISKELYEFCLD

QGYADRNLIAKWKKPGYERLCCLRCMQPRDHNFATTCVCRVPKQLREEKVIECVHCGCKGCASGD

>OAY42871.1 hypothetical protein MANES_08G022800 [Manihot esculenta]

MYKRKMPKVKTNRVKYPEGWELIEPTLRELQAKMREAENDTHDGKRKCEALWPIFKIAHQKSRYIFDLYHRRKEISKELY

EFCLDQGYADRNLIAKWKKPGYERLCCLRCMQPRDHNFATTCVCRVPKNLREEKVIECVHCGCRGCASGD

>XP_021620759.1 protein BUD31 homolog 1 [Manihot esculenta] >XP_021620760.1 protein BUD31 homolog 1 [Manihot esculenta]

MPKVKTNRVKYPEGWELIEPTLRELQAKMREAENDTHDGKRKCEALWPIFKIAHQKSRYIFDLYHRRKEISKELYEFCLD

QGYADRNLIAKWKKPGYERLCCLRCMQPRDHNFATTCVCRVPKNLREEKVIECVHCGCRGCASGD

>GAU12006.1 hypothetical protein TSUD_196300 [Trifolium subterraneum]

MPKVMTSRVKYPEGWELIEPTLRELQGKMREAENDPHDGKRKCETLWPIFKIAHQKSRYVFDLYHRRKEISKELYEFCLD

QGYADRNLIAKWKKPGYERLCCLRCIQPRDHNFATTCVCRVPKQLREEKVIECVHCGCKGCASGD

>KDO36895.1 hypothetical protein CISIN_1g032189mg [Citrus sinensis]

MPKVKTNRVKIPEGWELIEPTLRELQAKMREAENDPHDGKRKCETLWPIFKIAHQKSRYIFDLYYRRKEISKELYEFCLD

QGYADRNLIAKWKKPGYERLCCLRCMQPRDHNFQTTCVCRVPKNLREEKVIECVHCGCRGCASGD

>XP_020520470.1 protein BUD31 homolog 1 isoform X1 [Amborella trichopoda] >XP_020520471.1 protein BUD31 homolog 1 isoform X1 [Amborella trichopoda]

MLLVILTWFSTHWEDTMPKIKTNRVQYPGGWELIEPTLRELEAKMREAENDTHDGKRKCEALWPIFRIAHQKSRYIYDLY

YRRKEISKELYEFCLEQGYADRNLIAKWKKPGYERLCCLRCIQPRDHNFNTTCVCRVPKHLREEKVIECVHCGCRGCASG

D

>EMS52447.1 Protein BUD31-like protein 2 [Triticum urartu]

MPKIKTSRVKYPEGWELIEPTLRDLEAKMREAENDTHDGKRKCEALWPIFRISHQKSRYIYDLYYRRKEIKKELYEFCLD

QGYADKNLIAKWKKVGYERLCCLRCIQTRDHNFATTCVCRVPKHLREETVIECVHCGCKGCASGD

>XP_012066455.1 protein BUD31 homolog 2 [Jatropha curcas]

MPKVKTNRVKYPEGWELIEPTLRELQSKMREAENDPHDGKRKCEALWPIFKIAHQKSRYIFDLYHRRKEISKELYEFCLD

QGYADRNLIAKWKKPGYERLCCLRCMQPRDHNFATTCVCRVPKNLREEKVIECVHCGCRGCASGD

>XP_010679052.1 PREDICTED: protein BUD31 homolog 2 [Beta vulgaris subsp. vulgaris] >XP_010685537.1 PREDICTED: protein BUD31 homolog 2 [Beta vulgaris subsp. vulgaris] >KMT05040.1 hypothetical protein BVRB_7g172000 [Beta vulgaris subsp. vulgaris] >KMT10174.1 hypothetical protein BVRB_5g119350 [Beta vulgaris subsp. vulgaris]

MPKVKTNRVKYPEGWELIEPTLQELESKMREAQLDSHDGKRKCETLWPIFKIAHQKSRYLYDLYYRRNEISKELYEFCLD

QGYADRNLIAKWKKPGYERLCCLRCIQPRDHNFGTTCVCRVPKHLREEKVVECVHCGCGGCASGD

>XP_006471594.1 PREDICTED: protein BUD31 homolog 2 [Citrus sinensis] >XP_006471595.1 PREDICTED: protein BUD31 homolog 2 [Citrus sinensis]

MPKVKTNRVKIPEGWELIEPTLRELQAKMREAENDPHDGKRKCETLWPIFKIAHQKSRYIFDLYYRRKEISKELYEFCLD

QGYADCNLIAKWKKPGYERLCCLRCMQPRDHNFQTTCVCRVPKNLREEKVIECVHCGCRGCASGD

>XP_008373460.1 PREDICTED: protein BUD31 homolog 1 [Malus domestica]

MPKVKTNRVKYPDGWELIEPTLRDLQAKMREAENDTHDGKRKCETLWPIFKIAHQKSRYIFDLYHRRKEISKELYEFCLD

QGYADRNLIAKWKKPGYERLCCLRCMQPRDHNFATTCVCRVPKHLREEKVIECVHCGCXGCASGD

>CBX24472.1 hypothetical_protein [Oryza glaberrima]

MSPRGLTVGQTSRTHQAASSSFRPGPRSSVRPLLPPTCGDGTLKNFFFPFSSGQRKELYGDAATSPQISNLLMAGAGGFL

IVSPYAARRSIMPKIKTSGVKYPDGWELIEPTLSELHSKMREAENDPHDGRRKCEALWPIFKINHQRSRYLYDLYYNRKE

ISQELYEFCLDQGHADRNLIAKWKKQGYERLCCLRCIQTRDHNFATTCVCRVPKHLREEQVIECVHCGCKGCASGD

>XP_018839780.1 PREDICTED: protein BUD31 homolog 2 [Juglans regia] >XP_018839781.1 PREDICTED: protein BUD31 homolog 2 [Juglans regia] >XP_018839783.1 PREDICTED: protein BUD31 homolog 2 [Juglans regia] >XP_018839784.1 PREDICTED: protein BUD31 homolog 2 [Juglans regia] >XP_018839785.1 PREDICTED: protein BUD31 homolog 2 [Juglans regia] >XP_018839786.1 PREDICTED: protein BUD31 homolog 2 [Juglans regia] >XP_018839787.1 PREDICTED: protein BUD31 homolog 2 [Juglans regia]

MPKVKTNRVKYPEGWELIEPTLRELQAKMREAENDPHDGKRKCEALWPIFKIAHQKSRYIFDLYHRRKEISKELYEFCLD

QGYADRNIIAKWKKPGYERLCCLRCIQPRDHNFATTCVCRVPKHLREEKVIECVHCGCKGCASGD

>KMZ72633.1 putative Cell cycle control protein Cwf14/Bud31 [Zostera marina]

MPKVKTNRVKYPEGWELIEPTLRELEGKMREAENDPHDGKRKCEALWPIFKIAHQKSRYIYDLYYRRKEISKELYEFCLD

QGHADRNLIAKWKKPGYERLCCLRCIQPRDHNFQTTCVCRVPKHLREEKAIECVHCGCRGCASGD

>XP_009365750.1 PREDICTED: protein BUD31 homolog 1 [Pyrus x bretschneideri] >XP_009374754.1 PREDICTED: protein BUD31 homolog 1 [Pyrus x bretschneideri]

MPKVKTNRVKYPEGWELIEPTLRELQAKMREAENDTHDGKRKCETLWPIFKIAHQKSRYIFDLYHRRKEISKELYEFCLD

QGYADRNLIAKWKKPGYERLCCLRCMQPRDHNFATTCVCRVPKHLREEKVIECVHCGCRGCASGD

>XP_020225632.1 protein BUD31 homolog 2 [Cajanus cajan]

MPKVKTNRVKYPEGWELIEPTLRELQGKMREAENDPHDGKRKCETLWPIFKIAHQKSRYIFDLYHRRKEISKELYEFCLD

QGYADRNLIAKWKKPGYERLCCLRCMQPRDHNFATTCVCRVPKQLREEKVIECVHCGCKGCASGD

>XP_013598898.1 PREDICTED: protein BUD31 homolog 1-like [Brassica oleracea var. oleracea] >XP_013717276.1 PREDICTED: protein BUD31 homolog 1-like [Brassica napus] >XP_013717277.1 PREDICTED: protein BUD31 homolog 1-like [Brassica napus] >XP_013717482.1 PREDICTED: protein BUD31 homolog 1-like [Brassica napus] >XP_013717483.1 PREDICTED: protein BUD31 homolog 1-like [Brassica napus]

MPKVKTNRVKYPQGWELIEPTLRDLDAKMRQAEMDEHDGKRKCEALWPIFKLSHQRSRYVYDLYYRREEISKELYEFCLD

QGYADRNLIAKWKKSGYERLCCLRCIQPRDHNYGTTCVCRVPKHLREEKVVECVHCGCQGCASGD

>NP_001237676.1 uncharacterized protein LOC100305597 [Glycine max] >XP_003524924.1 PREDICTED: protein BUD31 homolog 2 [Glycine max] >XP_007160948.1 hypothetical protein PHAVU_001G030700g [Phaseolus vulgaris] >XP_014503731.1 PREDICTED: protein BUD31 homolog 2 [Vigna radiata var. radiata] >XP_014633798.1 PREDICTED: uncharacterized protein LOC100305597 isoform X1 [Glycine max] >XP_015958231.1 protein BUD31 homolog 2 [Arachis duranensis] >XP_016187703.1 protein BUD31 homolog 2 isoform X1 [Arachis ipaensis] >XP_017428294.1 PREDICTED: protein BUD31 homolog 2 [Vigna angularis] >XP_017428295.1 PREDICTED: protein BUD31 homolog 2 [Vigna angularis] >XP_017428296.1 PREDICTED: protein BUD31 homolog 2 [Vigna angularis] >ACU13359.1 unknown [Glycine max] >ESW32942.1 hypothetical protein PHAVU_001G030700g [Phaseolus vulgaris] >KHN12347.1 Protein BUD31

like 2 [Glycine soja] >KOM48926.1 hypothetical protein LR48_Vigan07g263000 [Vigna angularis] >KRH42745.1 hypothetical protein GLYMA_08G108500 [Glycine max] >KRH42746.1 hypothetical protein GLYMA_08G108500 [Glycine max] >KRH42747.1 hypothetical protein GLYMA_08G108500 [Glycine max] >KRH58844.1 hypothetical protein GLYMA_05G151700 [Glycine max] >BAT82572.1 hypothetical protein VIGAN_03261100 [Vigna angularis var. angularis]

MPKVKTNRVKYPEGWELIEPTLRELQAKMREAENDPHDGKRKCETLWPIFKIAHQKSRYIFDLYHRRKEISKELYEFCLD

QGYADRNLIAKWKKPGYERLCCLRCMQPRDHNFATTCVCRVPKQLREEKVIECVHCGCKGCASGD

>XP_011626777.1 protein BUD31 homolog 2 [Amborella trichopoda]

MPKIKTNRVQYPSGWELIEPTLREIEAKMREAENDPHDGKRKCEGLWPIFRIAHQKSRYIYDLYYRRKEISKELYEFCLE

QGYADRNLIAKWKKPGYERLCCLRCIQPRDHNFNTTCVCRVPKHLREEKVIECVHCGCRGCASGD

>XP_006397000.1 hypothetical protein EUTSA_v10028987mg [Eutrema salsugineum] >ESQ38453.1 hypothetical protein EUTSA_v10028987mg [Eutrema salsugineum]

MSSKRRGKRRTAGSDHKLKMYQGPVCSDPSLPQNSLILSSLNFSQKNMPKVKTNRVKYPEGWELIEPTLRELDAQMREAE

MDSHDGKRKCETLWPIFKVSHQRSRYVYDLYYRREEISKELYEFCLDQGYADRNLIAKWKKSGYERLCCLRCIQPRDHNY

GTTCVCRVPKNLREEKAVECVHCGCQGCASGD

>XP_010264950.1 PREDICTED: protein BUD31 homolog 2 [Nelumbo nucifera]

MPKIKTNRVKYPNGWELIEPTLRELEAKMREAENDPHDGKRKCEALWPIFKIAHQKSRYIYDLYYRRKEISKELYEFCLE

QGYADRNLIAKWKKSGYERLCCLRCIQPRDHNFNTTCVCRVPKHLREEKVIECVHCGCRGCASGD

>XP_002313660.2 G10 family protein [Populus trichocarpa] >XP_011044694.1 PREDICTED: protein BUD31 homolog 2 isoform X1 [Populus euphratica] >XP_011044700.1 PREDICTED: protein BUD31 homolog 2 isoform X2 [Populus euphratica] >XP_011044709.1 PREDICTED: protein BUD31 homolog 2 isoform X1 [Populus euphratica] >EEE87615.2 G10 family protein [Populus trichocarpa]

MPKVRTNRVKYPEGWELIEPTLRELDGKMREAELDPHDGKRKCEALWPIFKITHQKSRYVYDLYYRRSEISKELYEFCLD

QGYGDRNLIAKWKKPGYERLCCLRCIQPRDHNFGTTCVCRVPKHLREEKVVECVHCGCGGCASGD

>XP_012475822.1 PREDICTED: protein BUD31 homolog 2 [Gossypium raimondii] >XP_016716203.1 PREDICTED: protein BUD31 homolog 2 [Gossypium hirsutum] >XP_016703313.1 PREDICTED: protein BUD31 homolog 2 [Gossypium hirsutum] >XP_017624217.1 PREDICTED: protein BUD31 homolog 2 [Gossypium arboreum] >KHG22385.1 hypothetical protein F383_27796 [Gossypium arboreum] >KJB25466.1 hypothetical protein B456_004G193300 [Gossypium raimondii]

MPKVKTNRVKYPEGWELIEPTLRELQAKMREAENDPHDGKRKCETLWPIFKIAHQKSRYIFDLYHRRKEISKELYEFCLD

QGYADRNLIAKWKKPGYERLCCLRCMQPRDHNFATTCVCRVPKHLREEKVIECVHCGCKGCASGD

>XP_003629613.1 cell cycle control protein, G10 family protein [Medicago truncatula] >AET04089.1 cell cycle control protein, G10 family protein [Medicago truncatula] >AFK42295.1 unknown [Medicago truncatula] >AFK45259.1 unknown [Medicago truncatula]

MPKVKTNRVKYPEGWELIEPTLRELQGKMREAENDPHDGKRKCETLWPIFKIAHQKSRYVFDLYHRRKEISKELYEFCLD

QGYADRNLIAKWKKPGYERLCCLRCMQPRDHNFATTCVCRVPKQLREEKVIECVHCGCKGCASGD

>XP_015873429.1 PREDICTED: protein BUD31 homolog 2-like [Ziziphus jujuba] >XP_015873430.1 PREDICTED: protein BUD31 homolog 2-like [Ziziphus jujuba]

MPKVRTNRIKYPDGWELVEPTLGELDAKMREVQNDPHDGKRNCEGLWPIFKITHQKSRYIYDLYYKRNEISKELYEFCLD

QGHADRNLIAKWKKSGYERLCCLRCMQPRDHNFGTTCVCRVPKHLREEKIVECVHCGCKGCASGD

>XP_001771571.1 predicted protein [Physcomitrella patens] >EDQ63601.1 predicted protein [Physcomitrella patens]

MPKVRTNRTVYPEGWELIEPTLRELETKMREAENETHEGKRKCEALWPIFKISHQKSRYIYDLFYRRKAITRKLFDFCLE

QGHADKNLIAKWKKSGYERLCCLRCIQPRDHNYGTTCVCRVPKHLREEKVIECVHCGCHGCASGD

>XP_021655135.1 protein BUD31 homolog 1 [Hevea brasiliensis] >XP_021655136.1 protein BUD31 homolog 1 [Hevea brasiliensis]

MPKVKTNRVKYPEGWELIEPTLRELQAKMREAENDTHDGKRKCEALWPIFKIAHQKSRYIFDLYHRQKEISKELYEFCLD

QGYADRNLIAKWKKPGYERLCCLRCMQPRDHNFATTCVCRVPKNLREEKVIECVHCGCRGCASGD

>GAV57382.1 G10 domain-containing protein [Cephalotus follicularis]

MPKVKTNRVNYPEGWELIEPTLRELQAKMREAENDPHDGKRKCEALWPIFKIAHQKSRYIFDLYHRRKEISKELYEFCLD

QGYADRNLIAKWKKPGYERLCCLRCIQPRDHNFATTCVCRVPKNLREEKVIECVHCGCRGCASGD

>XP_009365369.1 PREDICTED: protein BUD31 homolog 1-like [Pyrus x bretschneideri]

MPKVKTNRVKYPEGWELIEPTLRELQAKMREAENDTHDGKRKCETLWPIFKIAHQKSRYIFDLYHRRKEISKELYEFCLD

QGYADRNLIAKWKKPGYERLCCLRCMQPRDHNFATTCVCRVPKYLREEKVIECVHCGCRGCASGD

>XP_007039701.1 PREDICTED: protein BUD31 homolog 2 [Theobroma cacao] >EOY24202.1 G10 family protein [Theobroma cacao]

MPKVKTNRVKYPEGWELIEPTLRELQAKMREAENDPHDGKRKCETLWPIFKIAHQKSRYIFDLYHRRKEISKELYEFCLD

QAYADRNLIAKWKKPGYERLCCLRCMQPRDHNFATTCVCRVPKHLREEKVIECVHCGCKGCASGD

>XP_015901947.1 PREDICTED: protein BUD31 homolog 2 [Ziziphus jujuba]

MPKVKTNRVKYPEGWELIEPTLRELQAKMREAENNPHDGKRKCEALWPIFKIAHQKSRYIFDLYHRRKEISKELYEFCMD

QGYADRNLIAKWKKPGYERLCCLRCMQPRDHNFATTCVCRVPKHLREEKVIECVHCGCRGCASGD

>XP_018469991.1 PREDICTED: protein BUD31 homolog 1 [Raphanus sativus] >XP_018469992.1 PREDICTED: protein BUD31 homolog 1 [Raphanus sativus] >XP_018466519.1 PREDICTED: protein BUD31 homolog 1 [Raphanus sativus] >XP_018466520.1 PREDICTED: protein BUD31 homolog 1 [Raphanus sativus]

MPKVKTNRVKYPEGWELIEPTLRELDAKMREAEMDTHDGKRKCEALWPIFKLSHQRSRYVYDLYYRRKEISKELYEFCLD

QGYADRNLIAKWKKSGYERLCCLRCIQPRDHNYGTTCVCRVPKHLREEKAIECVHCGCQGCASGD

>ERN15246.1 hypothetical protein AMTR_s00056p00205810 [Amborella trichopoda]

MFVGEDNRQRAGTMPKIKTNRVQYPSGWELIEPTLREIEAKMREAENDPHDGKRKCEGLWPIFRIAHQKSRYIYDLYYRR

KEISKELYEFCLEQGYADRNLIAKWKKPGYERLCCLRCIQPRDHNFNTTCVCRVPKHLREEKVIECVHCGCRGCASGD

>XP_009134394.1 PREDICTED: protein BUD31 homolog 1 [Brassica rapa] >XP_013639795.1 PREDICTED: protein BUD31 homolog 1 [Brassica oleracea var. oleracea] >XP_013735010.1 PREDICTED: protein BUD31 homolog 1 [Brassica napus] >XP_013735019.1 PREDICTED: protein BUD31 homolog 1 [Brassica napus] >XP_013671124.1 PREDICTED: protein BUD31 homolog 1 [Brassica napus]

MPKIKTNRVKYPEGWELIEPTLRELDAKMREAEMDEHDGKRKCEALWPIFKLSHQRSRYVYDLYYRRKEISKELYEFCLD

QGYADRNLIAKWKKSGYERLCCLRCIQPRDHNYGTTCVCRVPKHLREEKAIECVHCGCQGCASGD

>XP_009108510.1 PREDICTED: protein BUD31 homolog 1-like [Brassica rapa] >XP_009108511.1 PREDICTED: protein BUD31 homolog 1-like [Brassica rapa] >XP_013656148.1 PREDICTED: protein BUD31 homolog 1-like [Brassica napus] >XP_013656149.1 PREDICTED: protein BUD31 homolog 1-like [Brassica napus]

MPKIKTNRVKYPEGWELIEPTLRELDAKMREAEMDEHDGKRKCEALWPIFKLSHQRSRYVYDLYYRREEISKELYEFCLD

QGYADRSLIAKWKKSGYERLCCLRCIQPRDHNYGTTCVCRVPKHLREEKVVECVHCGCQGCASGD

>XP_011044716.1 PREDICTED: protein BUD31 homolog 2 isoform X3 [Populus euphratica]

MPKVRTNRVKYPEGWELIEPTLRELDGKMREAELDPHDGKRKCEALWPIFKITHQKSRYVYDLYYRRSEISKELYEFCLD

QGYGDRNLIAKWKKPGYERLCCLRCIQPRDHNFGTTCVCRVPKHLREERFVECVHCGCGGCASGD

>OTG09278.1 putative G10 protein [Helianthus annuus]

MFSKVKKIKNSRNRLQLDAPYKDLLTQRSPVTRSSSGQSTRRNHHNRTFGNTFSVGLMPKVKTNRVKYPEGWELVEPTLN

ELQAKMREAENDPHDGKRKCEALWPIFKIAHQKSRYIFDLYHRRKEISAELYEFCLDQGYADRNIIAKWKKPGYERLCCL

RCIQPRDHNFNTTCVCRVPKHLREEKVIECVHCGCKGCASGD

>XP_007202730.1 protein BUD31 homolog 1 [Prunus persica] >XP_008241275.1 PREDICTED: protein BUD31 homolog 1 [Prunus mume] >XP_021810171.1 protein BUD31 homolog 1 [Prunus avium] >ONH96234.1 hypothetical protein PRUPE_7G115100 [Prunus persica]

MPKVKTNRVKYPEGWELIEPTIRELQAKMREAENDTHDGKRKCETLWPIFKIAHQKSRYIFDLYHRRNEISKELYEFCLD

QGYADRNLIAKWKKPGYERLCCLRCMQPRDHNFATTCVCRVPKHLREEKVIECVHCGCKGCASGD

>XP_006432787.1 hypothetical protein CICLE_v10003137mg [Citrus clementina] >ESR46027.1 hypothetical protein CICLE_v10003137mg [Citrus clementina]

MAIFLFAVSNVSMCTRLHLNNIREYSTYNFLKGAPQSRLIVLVRHRYDTISFPHPKVLIIASTSAPSSLAVSDNRETQGR

KMPKVKTNRVKIPEGWELIEPTLRELQAKMREAENDPHDGKRKCETLWPIFKIAHQKSRYIFDLYYRRKEISKELYEFCL

DQGYADCNLIAKWKKPGYERLCCLRCMQPRDHNFQTTCVCRVPKNLREEKVIECVHCGCRGCASGD

>NP_193843.1 G10 family protein [Arabidopsis thaliana] >NP_001329615.1 G10 family protein [Arabidopsis thaliana] >XP_002869907.1 protein BUD31 homolog 1 [Arabidopsis lyrata subsp. lyrata] >AAK95313.1 AT4g21110/F7J7_50 [Arabidopsis thaliana] >CAA17530.1 G10-like protein [Arabidopsis thaliana] >CAB79111.1 G10-like protein [Arabidopsis thaliana] >AAN18110.1 At4g21110/F7J7_50 [Arabidopsis thaliana] >EFH46166.1 G10 family protein [Arabidopsis lyrata subsp. lyrata] >AEE84404.1 G10 family protein [Arabidopsis thaliana] >OAP00345.1 hypothetical protein AXX17_AT4G24440 [Arabidopsis thaliana] >ANM67813.1 G10 family protein [Arabidopsis thaliana]

MPKVKTNRVKYPEGWELIEPTLRELDAKMREAETDSHDGKRKCETLWPIFKVSHQRSRYVYDLYYRREEISKELYEFCLD

QGYADRSLIAKWKKSGYERLCCLRCIQPRDHNYGTTCVCRVPKHLREEKVVECVHCGCQGCASGD

>XP_019441699.1 PREDICTED: protein BUD31 homolog 2 [Lupinus angustifolius] >XP_019441700.1 PREDICTED: protein BUD31 homolog 2 [Lupinus angustifolius] >OIW12750.1 hypothetical protein TanjilG_24683 [Lupinus angustifolius]

MPKVKTNRVKYPEGWELIEPTIRELQAKMREAENDPHDGKRKCETLWPIFKIAHQKSRYIFDLYHRRKEISKELYEFCLD

QGYADRNLIAKWKKPGYERLCCLRCMQPRDHNFATTCVCRVPKQLREEKVIECVHCGCKGCASGD

>XP_013685695.1 PREDICTED: protein BUD31 homolog 1-like [Brassica napus] >XP_013685696.1 PREDICTED: protein BUD31 homolog 1-like [Brassica napus] >XP_013685697.1 PREDICTED: protein BUD31 homolog 1-like [Brassica napus]

MPKIKTNRVKYPEGWELIEPTLRELDAKMRQAEMDEHDGKRKCEALWPIFKLSHQRSRYVYDLYYRRDEISKELYEFCLD

QGYADRSLIAKWKKSGYERLCCLRCIQPRDHNYGTTCVCRVPKHLREEKVVECVHCGCQGCASGD

>XP_002273849.1 PREDICTED: protein BUD31 homolog 2 [Vitis vinifera]

MPKVKTNRVKYPEGWELIEPTLRELQGKMREAENDPHDGKRKCEALWPIFKIAHQKSRYIFDLYHRRKEISKELYEFCMD

QGYADRNLIAKWKKPGYERLCCLRCMQPRDHNFATTCVCRVPKHLREEKVIECVHCGCRGCASGD

>XP_012836106.1 PREDICTED: protein BUD31 homolog 2 [Erythranthe guttata] >EYU38622.1 hypothetical protein MIMGU_mgv1a015799mg [Erythranthe guttata]

MPKVKTNRVKYPEGWELIEPTLRELQAKMREAENDPHDGQRKCEALWPIFKIAHQQSRYVFDLYHRRHEISRELYEFCLD

QGYADRNLIAKWKKPGYERLCCLRCIQPRDHNFQTTCVCRVPKHLREEKVIECVHCGCGGCASGD

>XP_009590958.1 PREDICTED: protein BUD31 homolog 2 [Nicotiana tomentosiformis] >XP_009590960.1 PREDICTED: protein BUD31 homolog 2 [Nicotiana tomentosiformis] >XP_009590961.1 PREDICTED: protein BUD31 homolog 2 [Nicotiana tomentosiformis] >XP_009590962.1 PREDICTED: protein BUD31 homolog 2 [Nicotiana tomentosiformis] >XP_009590963.1 PREDICTED: protein BUD31 homolog 2 [Nicotiana tomentosiformis] >XP_009590964.1 PREDICTED: protein BUD31 homolog 2 [Nicotiana tomentosiformis] >XP_009791756.1 PREDICTED: protein BUD31 homolog 2 [Nicotiana sylvestris] >XP_009791757.1 PREDICTED: protein BUD31 homolog 2 [Nicotiana sylvestris] >XP_016487187.1 PREDICTED: protein BUD31 homolog 2 [Nicotiana tabacum] >XP_019228210.1 PREDICTED: protein BUD31 homolog 2 [Nicotiana attenuata] >OIT30896.1 protein bud31 -like 2 [Nicotiana attenuata]

MPKVKTNRVKYPEGWELIEPTLSELQAKMREAENDPHDGKRKCEALWPIFKIAHQKSRYIFDLYHRRKEISKELYEFCLD

QGYADKNLIAKWKKPGYERLCCLRCMQPRDHNFQTTCVCRVPQHLREEKVIECVHCGCKGCASGD

>XP_013737538.1 PREDICTED: protein BUD31 homolog 1-like [Brassica napus]

MPKVKTNRVKYPEGWELIEPTLRDLDAKMREAEMDEHDGKRKCEALWPIFKLSHQRSRYVYDLYYRREEISKELYEFCMDQGYADRN LIAKWKKSGYERLCCLRCIQPRDHNYGTTCVCRVPKHLREEKVVECVHCGCQGCASGD

>XP_006384643.1 hypothetical protein POPTR_0004s19770g [Populus trichocarpa] >XP_006384644.1 hypothetical protein POPTR_0004s19770g [Populus trichocarpa] >XP_006384645.1 G10 family protein [Populus trichocarpa] >XP_011005365.1 PREDICTED: protein BUD31 homolog 2-like [Populus euphratica] >XP_011005370.1 PREDICTED: protein BUD31 homolog 2-like [Populus euphratica] >XP_011005379.1 PREDICTED: protein BUD31 homolog 2-like [Populus euphratica] >ERP62440.1 hypothetical protein POPTR_0004s19770g [Populus trichocarpa] >ERP62441.1 hypothetical protein POPTR_0004s19770g [Populus trichocarpa] >ERP62442.1 G10 family protein [Populus trichocarpa]

MPKVRRSRIKYPEGWELIEPTLRELDGKMREAELDPHDGKRKCEALWPIFKITHQKSRYIYDLYYRRNEISKELYEFCLDQGYGDRN LIAKWKKPGYERLCCLRCIQPRDHNFGTTCVCRVPKHLREEKVVECVHCGCGGCASGD

Pezizomycotina

>OJJ89505.1 hypothetical protein ASPGLDRAFT_116072 [Aspergillus glaucus CBS 516.65]

MPPVRTSRNRKPPPDGFDEIEDTLLEFSNKMKDAENASHDGKKKHEMLWPIFQISHQRSRYIYDLYYEKQAISKQLYEWLLKNNYAD ANLIAKWKKQGYEKLCCLRCIQTKETNFNATCICRVPKAQLKEDQMIQCVSCGCRGCASSD

>EYE99524.1 G10 protein [Aspergillus ruber CBS 135680]

MPPVRTSRNRKPPPDGFDEIEDTLLEFSNKMKDAENASHDGKKKHEMLWPIFQISHQRSRYIYDLYYEKKAISKQLYEWLLKNNYAD ANLIAKWKKQGYEKLCCLRCIQTKETNFNATCICRVPKAQLKEDQMIQCVSCGCRGCASSD

>XP_001242472.1 cell cycle control protein cwf14 [Coccidioides immitis RS] >XP_003069683.1 Cell cycle control protein cwf14, putative [Coccidioides posadasii C735 delta SOWgp] >EER27538.1 Cell cycle control protein cwf14, putative [Coccidioides posadasii C735 delta SOWgp] >EFW22808.1 cell cycle control protein cwf14 [Coccidioides posadasii str. Silveira] >EAS30889.3 cell cycle control protein cwf14 [Coccidioides immitis RS] >KMM67394.1 cell cycle control protein cwf14 [Coccidioides posadasii RMSCC 3488] >KMP03476.1 cell cycle control protein cwf14 [Coccidioides immitis RMSCC 2394] >KMU73065.1 cell cycle control protein cwf14 [Coccidioides immitis RMSCC 3703] >KMU82968.1 cell cycle control protein cwf14 [Coccidioides immitis H538.4]

MPPIRTSRNRKPPPDGFDDIEDTLLEFSNKMKDAENSSHEGKKRHEVLWPIFQISHQRSRYIYDLYYEKEAISKQLYDWLLKNNYAD ANLIAKWKKQGYEKLCCLRCIQTKETNFNSTCICRVPKAQLKENQNIQCVSCGCRGCASSD

>XP_001218090.1 cell cycle control protein cwf14 [Aspergillus terreus NIH2624] >EAU30605.1 cell cycle control protein cwf14 [Aspergillus terreus NIH2624]

MPPVRTSRTRKPPPAGFDDIEDTLLEFSNKMKDAENASHDGKKKHEMVWPIFQISHQRSRYIYDLYYEKEAISKQLYDWLLKNNYAD
ANLIAKWKKQGYEKLCCLRCIQTKETNFNATCICRVPKAQLKEDQMIQCVSCGCRGCASSD

>XP_020053051.1 hypothetical protein ASPACDRAFT_54117 [Aspergillus aculeatus ATCC 16872] >OJJ96711.1 hypothetical protein ASPACDRAFT_54117 [Aspergillus aculeatus ATCC 16872]

MPPIRTSRTRKPPPAGFDDIEDTLLEFSNKMKDAENASHDGKKKHEMLWPIFQISHQRSRYIYDLYYEKEAISKQLYEWLLKNNYAD
SNLIAKWKKQGYEKLCCLRCIQTKETNFNATCICRVPRAQLKEDQTIQCVSCGCRGCASSD

>OOF92268.1 hypothetical protein ASPCADRAFT_56015 [Aspergillus carbonarius ITEM 5010]

MPPVRTSRNRKPPPAGFDDIEDTLLEFSNKMKDAENASHDGKKKHEMLWPIFQISHQRSRYIYDLYYEKEAISKQLYEWLLKNNYAD
ANLIAKWKKQGYEKLCCLRCIQTKETNFNATCICRVPKAQLKEDQTIQCVSCGCRGCASSD

>XP_754070.1 cell cycle control protein Cwf14/Bud31 [Aspergillus fumigatus Af293] >EAL92032.1 cell cycle control protein Cwf14/Bud31, putative [Aspergillus fumigatus Af293]

MPPLRTSRNRKPPPAGFDDIEDTLLEFSNKMKDAENAPHDGKKKHEMLWPIFQISHQRSRYIYDLYYEKEAISRQLYEWLLKNNYAD
ANLIAKWKKQGYEKLCCLRCIQTKETNFNATCICRVPKAQLKEDQMIQCVSCGCRGCASSD

>XP_001398454.1 cell cycle control protein cwf14 [Aspergillus niger CBS 513.88] >CAL00568.1 unnamed protein product [Aspergillus niger] >EHA22467.1 hypothetical protein ASPNIDRAFT_51095 [Aspergillus niger ATCC 1015] >OJI84316.1 hypothetical protein ASPTUDRAFT_43336 [Aspergillus tubingensis CBS 134.48] >OJJ68781.1 hypothetical protein ASPBRDRAFT_58110 [Aspergillus brasiliensis CBS 101740] >OJZ81745.1 hypothetical protein ASPFODRAFT_197572 [Aspergillus luchuensis CBS 106.47]

MPPVRTSRNRKPPPAGFDDIEDTLLEFSNKMKDAENASHDGKKKHEMLWPIFQISHQRSRYVYDLYYEKEAISKQLYEWLLKNNYAD
ANLIAKWKKQGYEKLCCLRCIQTKETNFNATCICRVPKAQLKEDQTIQCVSCGCRGCASSD

>XP_002382939.1 cell cycle control protein Cwf14/Bud31, putative [Aspergillus flavus NRRL3357] >EED46759.1 cell cycle control protein Cwf14/Bud31, putative [Aspergillus flavus NRRL3357] >KDE76232.1 G10 protein/predicted nuclear transcription regulator [Aspergillus oryzae 100-8] >KOC12770.1 cell cycle control protein [Aspergillus flavus AF70]

MPPIRTSRNRKPPPAGFDDIEDTLLEFSNKMKDAENAPHDGKKKHEMLWPIFQITHQRSRYIYDLYYEKEAISKQLYDWLLKNNYAD
ANLIAKWKKQGYEKLCCLRCIQTKETNFNATCICRVPKAQLKEDQMIQCVSCGCRGCASSD

>KJX99478.1 cell cycle control protein cwf14 [Zymoseptoria brevis]

MPAIRTAKNRKPPPEGFEDIEDTLLEFANKMKDAENASHEGKKKHEMLWPVFQITHQRSRYIYDLYYEKEAISRQLYDWLLKNNYAD
ANLIAKWKKQGYEKLCCLRCIQTKETNFNSTCICRVPKEKLSEDQEIQCVSCGCRGCSSSD

>EDP51312.1 cell cycle control protein Cwf14, putative [Aspergillus fumigatus A1163]

MPPIRTSRNRKPPPAGFDDIEDTLLEFSNKMKDAENAPHDGKKKHEMLWPIFQISHQRSRYIYDLYYEKEAISRQLYEWLLKNNYAD
ANLIAKWKKQGYEKLCCLRCIQTKETNFNATCICRVPKAQLKEDQMIQCVSCGCRGCASSD

>KKK19375.1 cell cycle control protein [Aspergillus ochraceoroseus] >CEL04329.1 Putative Cell cycle control protein cwf14 [Aspergillus calidoustus]

MPPVRTSRNRKPPPAGFDDIEDTLLEFSNKMKDAENAPHEGKKKHEVLWPIFQISHQRSRYIYDLYYEKEAISKQLYDWLLKNNYAD
ANLIAKWKKQGYEKLCCLRCIQTKETNFNATCICRVPKAQLKEDQMIQCVSCGCRGCASSD

>OJJ48776.1 hypothetical protein ASPZODRAFT_149776 [Penicilliopsis zonata CBS 506.65]

MPPIRTSRSRKTPPAGFEDIEDTLLEFSNKMKDAENASHEGKKKHEVLWPIFQISHQRSRYIYELYYEKEAISKQLYDWLLKNNYAD
ANLIAKWKKQGYEKLCCLRCIQTKETNFNSTCICRVPKAQLKEDQVIQCVSCGCRGCASSD

>XP_015405996.1 putative cell cycle control protein Cwf14/Bud31 [Aspergillus nomius NRRL 13137] >KNG85073.1 putative cell cycle control protein Cwf14/Bud31 [Aspergillus nomius NRRL 13137]

MPPIRTSRNRKPPPAGFDDIEDTLLEFSNKMKDAENAPHDGKKRHEMLWPIFQITHQRSRYIYDLYYEKEAISKQLYDWLLKNNYAD ANLIAKWKKQGYEKLCCLRCIQTKETNFNATCICRVPKAQLKEDQMMQCVSCGCRGCASSD

>XP_007927734.1 hypothetical protein MYCFIDRAFT_82344 [Pseudocercospora fijiensis CIRAD86] >EME82427.1 hypothetical protein MYCFIDRAFT_82344 [Pseudocercospora fijiensis CIRAD86]

MPAIRTAKNRKPPPDGFEDIEDTLLEFQNKMKDAENASHEGKKKHEMQWPIFQITHQRSRYIYDLYYEKEAISKKLYEWLLKNNYAD ANLIAKWKKQGYEKLCCLRCIQTKETNFNSTCICRVPREQLKEDQEIQCVGCGCRGCASSD

>XP_016760256.1 cell cycle control protein cwf14 [Sphaerulina musiva SO2202] >EMF12135.1 cell cycle control protein cwf14 [Sphaerulina musiva SO2202]

MPAIRTAKNRKPPPDGFEDIEDTLLEFQNKMKDAENASHEGKKKHEMLWPVFQITHQRSRYIYDLYYEKEAISKQLYDWLLKNGYAD ANLIAKWKKQGYEKLCCLRCIQTKETNFNSTCICRVPRAQLKEDQEIECVSCGCRGCSSND

>OJJ41302.1 hypothetical protein ASPWEDRAFT_102459 [Aspergillus wentii DTO 134E9]

MPPIRTSKNRKPPPAGFDDIEDTLLEFNNKMKDAENASHDGKKKHEMLWPIFQISHQRSKYIYDLYYEKEAISKQLYDWLLKNNYAD ANLIAKWKKQGYEKLCCLRCMQTKETNFNATCICRVPKAQLKEDQMIQCVSCGCRGCASSD

>XP_002849011.1 cell cycle control protein cwf14 [Arthroderma otae CBS 113480] >EEQ29126.1 cell cycle control protein cwf14 [Arthroderma otae CBS 113480] >EGD92893.1 cell cycle control protein [Trichophyton tonsurans CBS 112818] >EZF22487.1 hypothetical protein H100_04695 [Trichophyton rubrum MR850] >EZF35572.1 hypothetical protein H101_00912 [Trichophyton interdigitale H6] >EZF41362.1 hypothetical protein H102_04683 [Trichophyton rubrum CBS 100081] >EZF52283.1 hypothetical protein H103_04688 [Trichophyton rubrum CBS 288.86] >EZF62775.1 hypothetical protein H104_04674 [Trichophyton rubrum CBS 289.86] >EZF73404.1 hypothetical protein H105_04704 [Trichophyton soudanense CBS 452.61] >EZF84089.1 hypothetical protein H110_04684 [Trichophyton rubrum MR1448] >EZF94735.1 hypothetical protein H113_04722 [Trichophyton rubrum MR1459] >EZG06038.1 hypothetical protein H106_04508 [Trichophyton rubrum CBS 735.88] >EZG16331.1 hypothetical protein H107_04814 [Trichophyton rubrum CBS 202.88] >KDB33293.1 hypothetical protein H112_04687 [Trichophyton rubrum D6] >EGD88202.2 hypothetical protein TERG_04452 [Trichophyton rubrum CBS 118892]

MPPIRSARTRKAPPDGFDDIEDTLLEFSNKMKDAENASHEGKKRHEVLWPIFQISHQRSRYIYDLYYEKEAISKKLYDWLLKNGYAD ANLIAKWKKQGYEKLCCLRCIQTKETNFNATCICRVPKAQLKEDQGIQCVSCGCRGCSSSD

>OTA34593.1 Pre-mRNA-splicing factor cwf14 [Hortaea werneckii EXF-2000]

MPAIRTAKNRKPPPDGFDDIEDTLLEFSNKMKDAENASHEGKKKHEMQWPIFQITHQRSRYIYDLYYEKEAISKQLYDWLLKNGYAD ANLIAKWKKQGYEKLCCLRCIQTKETNFNSTCICRVPREQLKEDQEIQCVSCGCRGCASSD

>XP_003849915.1 hypothetical protein MYCGRDRAFT_46635 [Zymoseptoria tritici IPO323] >EGP84891.1 hypothetical protein MYCGRDRAFT_46635 [Zymoseptoria tritici IPO323] >SMQ53103.1 unnamed protein product [Zymoseptoria tritici ST99CH_3D7] >SMY26733.1 unnamed protein product [Zymoseptoria tritici ST99CH_1A5]

MPAIRTAKNRKPPPEGFEDIEDTLLEFANKMKDAENASHEGKKKHEMLWPVFQITHQRSKYIYDLYYEKEAISRPLYDWLLKNNYAD ANLIAKWKKQGYEKLCCLRCIQTKETNFNSTCICRVPKEKLSEDQEIQCVSCGCRGCSSSD

>OJD10705.1 hypothetical protein AJ78_08365 [Emergomyces pasteuriana Ep9510]

MPPIRTSRNRKPPPDGFDDIEDTLLEFSNKMKDAENASHEGKKKHEVLWPIFQISHARSRYIYDLYYEKEAISKQLYDWLLKNNYAD ALLIAKWKKQGYEKLCCLRCIQTKETNFNATCICRVPKAQLNEDQSIQCVSCGCRGCSSSD

>XP_002562628.1 Pc20g00660 [Penicillium rubens Wisconsin 54-1255] >CAP85395.1 Pc20g00660 [Penicillium rubens Wisconsin 54-1255] >KZN87519.1 Pre-mRNA-splicing factor [Penicillium chrysogenum] >OQE18440.1 hypothetical protein PENFLA_c021G05437 [Penicillium flavigenum] >OQE88892.1 hypothetical protein PENNAL_c0015G10788 [Penicillium nalgiovense]

MPPIRTSRNRKPPPAGFDDIEDTLLEFSNKMKDAENASHEGKKKYEVLWPIFQISHQRSRYIYDLYYEKEAISKELYDFLLKNKYAD
ANLIAKWKKQGYEKLCCLRCVQTKETNFNSTCICRVPKAQLKEDQTIQCVSCGCNGCGSSD

>XP_007781260.1 cell cycle control protein cwf14 [Coniosporium apollinis CBS 100218] >EON65943.1 cell cycle control protein cwf14 [Coniosporium apollinis CBS 100218]

MPPIRSARTRKPPPDGFDDIEDTLLEFANAMKDAENASHEGKRRAEVLWPIFQISHQRSRYIYDLYYEKEAISKQLYDWLLKNGYAD
ANLIAKWKKQGYEKLCCLRCIQTKETNFNSTCICRVPRDKLAENQTIECVSCGCHGCASSD

>XP_001817116.1 cell cycle control protein cwf14 [Aspergillus oryzae RIB40] >BAE55114.1 unnamed protein product [Aspergillus oryzae RIB40]

MPPIRTSRNRKPPPAGFDDIEDTLLEFSNKMKDAENAPHDGKKKHEMLWSIFQITHQRSRYIYDLYYEKEAISKQLYDWLLKNNYAD
ANLIAKWKKQGYEKLCCLRCIQTKETNFNATCICRVPKAQLKEDQMIQCVSCGCRGCASSD

>OJJ06907.1 hypothetical protein ASPVEDRAFT_141222 [Aspergillus versicolor CBS 583.65]

MPPVRTSRNRKPPPAGFDDIEDTLLEFSNKMKDAENAPHEGKKKHEMLWPIFQISHQRSRYIYDLYYEKEAISKQLYDWLLKNGYAD
PNLIAKWKKQGYEKLCCLRCIQTKETNFNATCICRVPKAQLKEDQIIQCVSCGCRGCASSD

>OQD80754.1 hypothetical protein PENANT_c033G01979 [Penicillium antarcticum]

MPPIRTSRNRKPPPAGFDDIEDTLLEFSNKMKDAENASHEGKKKYEVLWPIFQISHQRSRYIYDLYYEKEAISKQLYDWLLKNGYAD
ANLIAKWKKQGYEKLCCLRCVQTKETNFNSTCICRVPKAQLKEDQTIQCVSCGCRGCGSSD

>XP_002621828.1 cell cycle control protein cwf14 [Blastomyces gilchristii SLH14081] >EGE86358.1 cell cycle control protein cwf14 [Blastomyces dermatitidis ATCC 18188] >EQL29098.1 hypothetical protein BDFG_08224 [Blastomyces dermatitidis ATCC 26199] >OAT12308.1 cell cycle control protein cwf14 [Blastomyces gilchristii SLH14081]

MPPIRSSRTRKPPPDGFDDIEDTLLEFSNKMKDAENASHEGKKRHEVLWPIFQISHARSRYIYDLYYEKEAISKQLYDWLLKNNYAD
ALLIAKWKKQGYEKLCCLRCIQTKETNFNATCICRVPKAQLKEDQSIQCVSCGCRGCSSSD

>OJD23380.1 hypothetical protein ACJ73_05263 [Blastomyces percursus]

MPPIRSSRTRKPPPDGFDDIENTLLEFSNKMKDAENASHEGKKRHEVLWPIFQISHARSRYIYDLYYEKEAISKQLYDWLLKNNYAD
ALLIAKWKKQGYEKLCCLRCIQTKETNFNATCICRVPKAQLKEDQSIQCVSCGCRGCSSSD

>CEJ54753.1 Putative Cell cycle control protein cwf14 [Penicillium brasilianum]

MPPIRTARNRKPPPAGFDDLEDTLLEFSNKMKDAENAPHEGKKKHEVLWPIFQISHQRSRYIYDLYYEKEAISKQLYDWLLKNNYAD
ANLIAKWKKQGYEKLCCLRCVQTKETNFNSTCICRVPKAQLKEEQTIQCVSCGCRGCASSD

>OAX78632.1 hypothetical protein ACJ72_07061 [Emmonsia sp. CAC-2015a]

MPPIRTSRNRKPPPDGFDDIEDTLLEFSNKMKDAENASHEGKKKHEVLWPIFQISHARSRYIYDLYYEKEAISKQLYDWLLKNNYAD
ALLIAKWKKQGYEKLCCLRCIQTKETNFNATCICRVPKAQLKEDQSIQCVSCGCRGCSSSD

>EEH10525.1 cell cycle control protein [Histoplasma capsulatum G186AR] >EER40837.1 cell cycle control protein cwf14 [Histoplasma capsulatum H143] >EGC45064.1 cell cycle control protein [Histoplasma capsulatum H88]

MPPIRTSRTRKPPPDGFDDIEDTLLEFSNKMKDAENASHEGKKRHEVLWPIFQISHARSRYIYDLYYEKEAISKQLYDWLLKNNYGD
ALLIAKWKKQGYEKLCCLRCIQTKETNFNATCICRVPKAQLKEDQSIQCVSCGCRGCSSSD

>OJJ55321.1 hypothetical protein ASPSYDRAFT_158987 [Aspergillus sydowii CBS 593.65]

MPPVRTSRNRKPPPAGFDDIEDTLLEFSNKMKDAENAPHEGKKKHEVLWPIFQISHQRSRYIYDLYYEKEAISKQLYDWLLKNGYAD
SNLIAKWKKQGYEKLCCLRCIQTKETNFNATCICRVPKAQLKEDQIIQCVSCGCRGCASSD

>KKZ60649.1 hypothetical protein EMCG_04671 [Emmonsia crescens UAMH 3008]

MPPIRTSRNRKPPPDGFDDIEDTLLEFSNKMKDAENASHDGKKRHEVLWPIFQISHARSRYIYDLYYEKEAISKQLYDWLLKNNYAD
ALLIAKWKKQGYEKLCCLRCIQTKETNFNATCICRVPKAQLKEDQSIQCVSCGCRGCSSSD

>CBF77865.1 TPA: cell cycle control protein Cwf14/Bud31, putative (AFU_orthologue; AFUA_5G05610) [Aspergillus nidulans FGSC A4]

MPPIRTSRNRKPPPAGFDDIEDTLLEFSNKMKDAENAPHEGKKKHEVLWPIFQITHQRSRYIYDLYYQKEAISKQLYEWLLKNGYAD ANLIAKWKKQGYEKLCCLRCIQTKETNFNATCICRVPKAQLKEDQIIQCVSCGCRGCASSD

>OKO89707.1 Pre-mRNA-splicing factor cwf14 [Penicillium subrubescens]

MPPVRTAARNRKPPPAGFDDIEDTLLEFSNKMKDAENESHEGKKKHEVLWPIFQISHQRSRYIYDLYYEKEAISKQLYDWLLKNNYA DANLIAKWKKQGYEKLCCLRCVQTKETNFNSTCICRVPKAQLKEEQTIQCVSCGCRGCASSD

>OQE36137.1 hypothetical protein PENCOP_c012G06937 [Penicillium coprophilum]

MPPIRTSRNRKPPPAGFDDLEDTLLEFSNKMKDAENASHEGKKKYEVLWPIFQISHQRSRYIYELYYEKEAISKELYDYLLKNKYAD ANLIAKWKKQGYEKLCCLRCVQTKETNFNSTCICRVPKAQLKEDQTIQCVSCGCNGCGSSD

>EME43506.1 hypothetical protein DOTSEDRAFT_72770 [Dothistroma septosporum NZE10]

MPAIRTSKNRKPPPDGFDDLEDTLLEFSNKMKDAENASHEGKKKHEMQWPIFQITHQRSRYIYDLYYEKEAISKKLYDWLLKNGYAD ANLIAKWKKQGYEKLCCLRCIQTKETNFNSTCICRVPREQLKEDQEIQCVSCGCRGCSSGD

>OCK83989.1 cell cycle control protein cwf14 [Lepidopterella palustris CBS 459.81]

MPVYRTGARNKKPPPDGFEDIEDTLLEFANKMKDAENASHEGKKKYEMLWPIFQITHQRSRYIYDLYYEKEAISKTLYDWLLKNNYA DANLIAKWKKQGYEKLCCLRCIQTKETNFSSTCICRVPKAQLKDDQTVQCVSCGCRGCSSGD

>KXG52367.1 G10 protein [Penicillium griseofulvum]

MPPIRTSRNRKPPPAGFDDLEDTLLEFSNKMKDAENASHEGKKKHEVLWPIFQISHQRSRYIYELYYEKEAISKELYDFLLKNKYAD ANLIAKWKKQGYEKLCCLRCVQTKETNFNSTCICRVPKAQLKEDQTIQCVSCGCNGCGSSD

>XP_016597290.1 G10 protein [Penicillium expansum] >KGO39897.1 G10 protein [Penicillium expansum] >KGO55060.1 G10 protein [Penicillium expansum] >KGO71382.1 G10 protein [Penicillium expansum] >KOS39793.1 hypothetical protein ACN38_g9366 [Penicillium nordicum] >OQD95711.1 hypothetical protein PENSOL_c019G08399 [Penicillium solitum]

MPPIRTSRNRKPPPAGFDDLEDTLLEFSNKMKDAENASHEGKKKYEVLWPIFQISHQRSRYIYELYYEKEAISKELYDFLLKNKYAD ANLIAKWKKQGYEKLCCLRCVQTKETNFNSTCICRVPKAQLKEDQTIQCVSCGCNGCGSSD

>OQO03671.1 Pre-mRNA-splicing factor cwf14 [Rachicladosporium antarcticum] >OQO04593.1 Pre-mRNA-splicing factor cwf14 [Rachicladosporium antarcticum] >OQO24380.1 Pre-mRNA-splicing factor cwf14 [Rachicladosporium sp. CCFEE 5018] >OQO24721.1 Pre-mRNA-splicing factor cwf14 [Rachicladosporium sp. CCFEE 5018]

MPAIRTAKARKAPPDGFEDLEDTLLEFQNKMKDAENASHEGKKKHEMIWPVFQITHQRSRYIYDLYYTKEAISKPLYDWLLKNQYAD ANLIAKWKKNGYEKLCCLRCIQTKETNFNSTCICRVPREQLKEDQEIQCVSCGCRGCSSGD

>KUM62804.1 hypothetical protein ACN42_g4292 [Penicillium freii]

MPPIRTSRNRKPPPAGFDDLEDTLLEFSNKMKDAENASHEGKKKYEVLWPIFQISHQRSRYVYELYYEKEAISKELYDFLLKNKYAD ANLIAKWKKQGYEKLCCLRCVQTKETNFNSTCICRVPKAQLKEDQTIQCVSCGCNGCGSSD

>EPS27364.1 hypothetical protein PDE_02307 [Penicillium oxalicum 114-2]

MPPVRTAARNRKPPPAGFDDIEDTLLEFGNKMKDAENASHEGKKKHEVLWPIFQISHQRSRYIYDLYYEKEAISKQLYDWLLKNNYA DANLIAKWKKQGYEKLCCLRCVQTKETNFNSTCICRVPKAQLKDEQSIQCVSCGCRGCASSD

>XP_014537001.1 Cell cycle control protein Cwf14/Bud31, putative [Penicillium digitatum Pd1] >EKV05196.1 Cell cycle control protein Cwf14/Bud31, putative [Penicillium digitatum PHI26] >EKV19742.1 Cell cycle control protein Cwf14/Bud31, putative [Penicillium digitatum Pd1]

MPPIRTSRNRKPPPAGFDDIEDTLLEFSNKMKDAENASHEGKKKYEVLWPIFQISHQRSRYIYELYYEKEAISKELYDFLLKNKYAD ANLIAKWKKQGYEKLCCLRCVQTKETNFNSTCICRVPKAQLKENQTIQCVSCGCNGCGSSD

>KEQ89551.1 G10 protein [Aureobasidium pullulans EXF-150]

MPPIRSSRNRKPPPDGFEDIEDTLLEFSNKLKDAENASHEGKKKHEMVWPVFQITHQRSKYIYDLYYEKEAISKQLYEWLLKNNYAD KNLIAKWKKQGYEKLCCLRCIQTKETNFNSTCICRVPRKQLKEDQVIQCVNCGCRGCGSSD

>CDM28100.1 Cell cycle control protein cwf14 [Penicillium roqueforti FM164] >CRL19059.1 G10 protein [Penicillium camemberti] >OQE08527.1 hypothetical protein PENVUL_c009G04264 [Penicillium vulpinum]

MPPIRTARNRKPPPAGFDDLEDTLLEFSNKMKDAENASHEGKKKYEVLWPIFQISHQRSRYIYELYYEKEAISKELYDFLLKNKYAD
ANLIAKWKKQGYEKLCCLRCVQTKETNFNSTCICRVPKAQLKEDQTIQCVSCGCNGCGSSD

>OQD67646.1 hypothetical protein PENPOL_c003G04295 [Penicillium polonicum]

MPPIRTSRNRKPPPAGFDDLEDTLLEFSNKMKDAENASHEGKKKYEVLWPIFQISHQRSRYIYELYYEKEAISKELYDFLLKNKYAD
ANLIAKWKKQGYEKLCCLRCVQTKETNFNGTCICRVPKAQLKEDQTIQCVSCGCNGCGSSD

>KEQ66869.1 G10 protein [Aureobasidium melanogenum CBS 110374]

MPPIRSSKNRKPPPDGFEDIEDTLLEFSNKLKDAENASHEGKKKHEMVWPVFQITHQRSKYIYDLYYEKEAISKQLYEWLLKNNYAD
KNLIAKWKKQGYEKLCCLRCIQTKETNFNSTCICRVPRKQLKEDQVIQCVNCGCRGCGSSD

>XP_013425087.1 putative cell cycle control protein Cwf14 [Aureobasidium namibiae CBS 147.97] >KEQ71056.1 putative cell cycle control protein Cwf14 [Aureobasidium namibiae CBS 147.97]

MPPIRSSRNRKPPPDGFEDIEDTLLEFSNKLKDAENASHEGKKKQEMVWPVFQITHQRSKYIYDLYYEKEAISRQLYEWLLKNNYAD
KNLIAKWKKQGYEKLCCLRCIQTKETNFNSTCICRVPRKNLKEDQVIECVNCGCRGCCSAD

>OCK98055.1 cell cycle control protein cwf14 [Cenococcum geophilum 1.58] >OCL06497.1 cell cycle control protein cwf14 [Glonium stellatum]

MPVYRTGARNKKPPPDGFEDIEDTLLEFSNKMKDAENASHEGKKKYEMLWPIFQITHQRSRYIYDLYYEKEAISKTLYDWLLKNGYA
DANLIAKWKKQGYEKLCCLRCIQTKETNFNSTCICRVPKDKLQENQTIECVSCGCRGCSSSD

>XP_007675249.1 hypothetical protein BAUCODRAFT_33107 [Baudoinia panamericana UAMH 10762] >EMC97392.1 hypothetical protein BAUCODRAFT_33107 [Baudoinia panamericana UAMH 10762]

MPAIRGTGKNRKPPPDGFDDIEDTLLEFSNKMKDAENASHEGKKKHEMQWPIFQITHQRSRYIYDLYYEKEAISKQLYDWLLKNGYA
DANLIAKWKKQGYEKLCCLRCIQTKETNFNSTCICRVPRAQLKEDQEIQCVSCGCRGCSSGD

>KLJ10438.1 hypothetical protein EMPG_14175 [Emmonsia parva UAMH 139]

MPPIRSSRTRKPPPDGFDDIEDTLLEFSNKMKDAENASHEGKRRHEVLWPIFQISHARSRYIYDLYYEKEAISKQLYDWLLKNNYAD
AMLIAKWKKQGYEKLCCLRCIQTKETNFNATCICRVPKAQLKEDQIQCVSCGCRGCSSSD

>XP_013345464.1 hypothetical protein AUEXF2481DRAFT_57755, partial [Aureobasidium subglaciale EXF-2481] >KEQ96983.1 hypothetical protein AUEXF2481DRAFT_57755, partial [Aureobasidium subglaciale EXF-2481]

MPPIRSSRNRKPPPDGFEDIEDTLLEFSNKLKDAENASHEGKKKHEMVWPVFQITHQRSKYIYDLYYEKEAISKQLYEWLLKNNYAD
KNLIAKWKKQGYEKLCCLRCIQTKETNFNSTCICRVPRKQLKEDQTIQCVSCGCRGCGS

>KXL43677.1 hypothetical protein FE78DRAFT_189516 [Acidomyces richmondensis] >KYG44469.1 hypothetical protein M433DRAFT_91412 [Acidomyces richmondensis BFW]

MPAIRGTGRNRKPPPDGFEDIEDTILEFSNKMKDAENASHEGKKKHEMQWPIFQITHQRSRYIYDLYYEKEAISKELYDWLLKNKYA
DANLIAKWKKQGYEKLCCLRCIQTKETNFNSTCICRVPRAQLKEDQEIQCVNCGCRGCSSND

>OTA34469.1 Pre-mRNA-splicing factor cwf14 [Hortaea werneckii EXF-2000]

MPAIRTAKNRKPPPDGFDDIEDTLLEFSNKMKDAENASHEGKKKHEMQWPIFQITHQREGREGSRYIYDLYYEKEAISKQLYDWLLK
NGYADANLIAKWKKQGYEKLCCLRCIQTKETNFNSTCICRVPREQLKEDQEIQCVSCGCRGCASSD

>XP_002148172.1 cell cycle control protein Cwf14/Bud31, putative [Talaromyces marneffei ATCC 18224] >EEA24661.1 cell cycle control protein Cwf14/Bud31, putative [Talaromyces marneffei ATCC 18224]

MPPIRTTRNAKPPPAGFDDIEDTLLEFSNRMKDAENAPHEGKKRHEVLWPVFQISHQRSRYIYELYYEKEAISKQLYDWLLKNKYAD
AMLIAKWKKQGYEKLCCLRCIQTKETNFNSTCICRVPKAQLKEEQAIQCVNCGCRGCSSSD

>XP_002482428.1 cell cycle control protein Cwf14/Bud31, putative [Talaromyces stipitatus ATCC 10500] >EED18436.1 cell cycle control protein Cwf14/Bud31, putative [Talaromyces stipitatus ATCC 10500]

MPPIRTTRNAKPPPAGFDDIEDTLLEFSNKMKDAENVSHEGKKRHEVLWPIFQISHQRSRYIYELYYEKEAISKQLYDWLLKNKYAD AMLIAKWKKQGYEKLCCLRCIQTKETNFNSTCICRVPKAQLKEEQAIQCVSCGCRGCSSSD

>XP_001931505.1 cell cycle control protein cwf14 [Pyrenophora tritici-repentis Pt-1C-BFP] >XP_003303868.1 hypothetical protein PTT_16252 [Pyrenophora teres f. teres 0-1] >EDU40610.1 cell cycle control protein cwf14 [Pyrenophora tritici-repentis Pt-1C-BFP] >EFQ88040.1 hypothetical protein PTT_16252 [Pyrenophora teres f. teres 0-1]

MPPVRTARASRKAPPEGFDDIEDTLLEFQNKMKDAENASHEGKKKYEMTWPIFQIVHQRSRYIYDLYYEKEAISKQLYDYLLKNGYA DAMLIAKWKKQGYEKLCCVRCIQTKETNFRSTCICRVPKDALKENQDIQCVNCGCRGCASSD

>XP_002790850.1 cell cycle control protein cwf14 [Paracoccidioides lutzii Pb01] >EEH36969.1 cell cycle control protein cwf14 [Paracoccidioides lutzii Pb01]

MPPIRASRNRKPPPDGFADIEDTLLEFSNKMKDAENASHEGKKRHEVHWPIFQISHARSRYIYDLYYEKEAISKQLYDWLLKNNYAD ALLIAKWKKQGYEKLCCLRCIQTKETNFNATCICRVPKAQLKEDQSIQCVSCGCRGCSSSD

>KGO76592.1 G10 protein [Penicillium italicum]

MPPIRTARNRKPPPAGFDDLEDTLLEFSNKMKDAENASHEGKKKYEVLWPIFQITHQRSRYIYELYYEKEAISKELYDFLLKNKYAD ANLIAKWKKQGYEKLCCLRCVQTKETNFNSTCICRVPKAQLKEGHTIQCVSCGCNGCGSSD

>ODH51724.1 hypothetical protein GX48_02191 [Paracoccidioides brasiliensis]

MPPIRASRNRKPPPDGFADIEDTLLEFSNKMKDAENASHEGKKRHEVHWPIFQISHARSRYIYDLYYEKEAISKQLYDWLLKNNYAD ALLIAKWKKQGYEKLCCLRCIQTKETNFNATCICRVPKAQLKEDQIIQCVSCGCRGCSSSD

>XP_002838335.1 hypothetical protein [Tuber melanosporum Mel28] >CAZ82526.1 unnamed protein product [Tuber melanosporum]

MPPIRTSRNRKPPPEGFDDIEDTLLEFANKMKDAENAPHEGKRKNESTWAIFQISHQRSRYIYDLYYDKEAINKDLYNWLLKNGYGD ANLIAKWKKQGYEKLCCLRCIQAKENNFNATCICRVPKARLGKEQVVECVSCGCRGCASGD

>XP_008023042.1 hypothetical protein SETTUDRAFT_167748 [Setosphaeria turcica Et28A] >EOA89202.1 hypothetical protein SETTUDRAFT_167748 [Setosphaeria turcica Et28A]

MPPVRTARASRKAPPEGFDDIEDTLLEFQTKMRDAENASHEGKKKYEMTWPIFQISHQRSRYIYDLYYNKEAISRQLYDYLLKNGYA DPMLIAKWKKQGYEKLCCTRCIQTKETNFRSTCICRVPKDQLNESQEIQCVNCGCRGCSSSD

>XP_018386630.1 cell cycle control protein cwf14 [Alternaria alternata] >OAG21209.1 cell cycle control protein cwf14 [Alternaria alternata] >OWY48103.1 cell cycle control protein cwf14 [Alternaria alternata]

MPPVRTARASRKAPPEGFDDIEDTLLEFQNKMKDAENASHEGKKKYEMTWPIFQITHQRSRYIYDLYYEKEAITKQLYDYLLKNGYA DAMLIAKWKKQGYEKLCCTRCIQTKETNFRSTCICRVPRDQLKENQEIQCVNCGCRGCASSD

>XP_018035848.1 cell cycle control protein cwf14 [Paraphaeosphaeria sporulosa] >OAG05483.1 cell cycle control protein cwf14 [Paraphaeosphaeria sporulosa]

MPPVRTARASKKAPPEGFDDIEDTLLEFAAAMKDAENASHDGKKKYEMTWPIFQITHQRSRYIYDMYYEKEAISKQLYDWLLKNGYA DAMLIAKWKKQGYEKLCCTRCIQTKETNFRSTCICRVPKETLKEGQEVQCVNCGCRGCASSD

>XP_016218938.1 hypothetical protein PV09_00018 [Verruconis gallopava] >XP_016218939.1 hypothetical protein, variant [Verruconis gallopava] >KIW09069.1 hypothetical protein PV09_00018 [Verruconis gallopava] >KIW09070.1 hypothetical protein, variant [Verruconis gallopava]

MPPIRSSRNRKPPPAGFEDIEDTLLEFDTKLKDATNASSEGKKRYESTWPIFQITHQRSRYIYDLYYEKEAISKQLYDWLLKNGYAD GNLIAKWKKQGYEKLCCLRCIQTNETNFKSTCICRVPRDQLKEDHTIECVNCGCRGCASSD

>XP_007697174.1 hypothetical protein COCSADRAFT_83648 [Bipolaris sorokiniana ND90Pr] >XP_007715738.1 hypothetical protein COCCADRAFT_105139 [Bipolaris zeicola 26-R-13]

>XP_014082233.1 hypothetical protein COCC4DRAFT_130572 [Bipolaris maydis ATCC 48331] >XP_014553007.1 hypothetical protein COCVIDRAFT_108831 [Bipolaris victoriae FI3] >EMD67544.1 hypothetical protein COCSADRAFT_83648 [Bipolaris sorokiniana ND90Pr] >EMD92628.1 hypothetical protein COCHEDRAFT_1135412 [Bipolaris maydis C5] >ENI08324.1 hypothetical protein COCC4DRAFT_130572 [Bipolaris maydis ATCC 48331] >EUC29950.1 hypothetical protein COCCADRAFT_105139 [Bipolaris zeicola 26-R-13] >EUN23431.1 hypothetical protein COCVIDRAFT_108831 [Bipolaris victoriae FI3]

MPPVRTARASRKAPPEGFDDIEDTLLEFQTKMKDAENASHEGKKKYEMTWPIFQITHQRSRYIYDLYYTKEAISKQLYDYLLKNGYA
DPMLIAKWKKQGYEKLCCTRCIQTKETNFRSTCICRVPRDQLKENQEIECVNCGCRGCASSD

>XP_011111300.1 hypothetical protein H072_5420 [Dactylellina haptotyla CBS 200.50] >EPS40696.1 hypothetical protein H072_5420 [Dactylellina haptotyla CBS 200.50]

MPPIRTRNKRKPPPDGFDDIEDTLLEFENKMKDAENAPHEGKRKNEMLWPIFQISHQRSRYIYDMYLKEGITRELYDWLLKNKYADA
LLIAKWKKQGYEKLCCLKCIQTKETNFGSTCICRVPKARMSKETVVECVSCGCHGCASSD

>OSS45252.1 hypothetical protein B5807_10435 [Epicoccum nigrum]

MPPVRTARASKKAPPDGFEDLEDTLLEFQNKMKDAENASHEGKKKYEMTWPIFQITHQRSRYIYDMYYEKEAISKQLYDYLLKNGYA
DPMLIAKWKKQGYEKLCCTRCIQTKETNFRSTCICRVPKEQMKENQDIQCVNCGCRGCASSD

>OAL02679.1 cell cycle control protein cwf14 [Stagonospora sp. SRC1lsM3a]

MPPVRTARASKKAPPEGFEDIEDTLLEFQNKMKDAENASHDGKKKYEMTWPIFQITHQRSRYIYDLYYEKEAISKQLYDYLLKNGYA
DAMLIAKWKKQGYEKLCCTRCIQTKETNFRSTCICRVPRETLKENQDIQCVNCGCRGCASSD

>XP_007684450.1 hypothetical protein COCMIDRAFT_85638 [Bipolaris oryzae ATCC 44560] >EUC49063.1 hypothetical protein COCMIDRAFT_85638 [Bipolaris oryzae ATCC 44560]

MPPVRTARASRKAPPEGFDDIEDTLLEFQTKMKDAENASHEGKKKYEMTWPIFQITHQRSRYIYDLYYTKEAISKQLYDYLLKNGYA
DPMLIAKWKKQGYEKLCCTRCIQTKETNFRSTCICRVPGDQLKENQEIECVNCGCRGCASSD

>ORX96286.1 cell cycle control protein cwf14 [Clohesyomyces aquaticus]

MPPIRTSTRTRKPPPEGFDEIEDTLLEFQNSMKDAENASHEGKKRYETTWPIFQIVHQRSRYIYDLYYEKEAISKTLYDWLLKNGYA
DAMLIAKWKKQGYEKLCCLRCIQTKETNFKSTCICRVPKDTLKEGKDIQCVSCGCRGCASGD

>KZM19376.1 nucleus [Ascochyta rabiei]

MPPIRTARASKKAPPDGFEDLEDTLLEFQNKMKDAENASHEGRKKYEMTWPIFQITHQRSRYIYDMYYEKEAISKQLYDYLLKNGYA
DAMLIAKWKKQGYEKLCCTRCIQTKETNFRSTCICRVPKGQMKEGQDIQCVNCGCRGCGSSD

>KXT03497.1 hypothetical protein AC578_1582 [Mycosphaerella eumusae]

MPAIRTAKNRKAPPGKKSPKFAVFQNKMKDAENASHEGKKKHEMQWPIFQITHQRSRYIYDLYYEKEAISKKLYEWLLKNNYADANL
IAKWKKQGYEKLCCLRCIQTKETNFNSTCICRVPREQLKEDQEIQCVSCGCRGCASSD

>OAL51363.1 cell cycle control protein cwf14 [Pyrenochaeta sp. DS3sAY3a]

MPPVRTARGSKKGPPEGFEDIEDALLEFQNKMKDAENASHEGKKKYEMTWPIFQITHQRSKYIYDLYYEKEAISKQLYDYLLKNGYA
DPMLIAKWKKQGYEKLCCTRCIQTKETNFRSTCICRVPKEQLKENQDIQCVNCGCRGCASSD

>KIM94954.1 hypothetical protein OIDMADRAFT_134985 [Oidiodendron maius Zn]

MPAIRHASKRKAPPAGFSDIEDDLLVFSNKMKDAENASSDNVPRHQVLWPIFQISHQRSRYVYDLYYEKEAISKELYDWLLKNGYAD
AMLIAKWKKTGYEKLCCLKCVQTKETNFNSTCICRVPKAQMKEDQVVECVSCGCRGCASSD

>XP_008088983.1 hypothetical protein GLRG_00107 [Colletotrichum graminicola M1.001] >EFQ24963.1 hypothetical protein GLRG_00107 [Colletotrichum graminicola M1.001] >KZL66107.1 cell cycle control protein cwf14 [Colletotrichum tofieldiae] >OHW93563.1 cell cycle control protein cwf14 [Colletotrichum incanum]

MPPMRSSKRKPPPEGFSDIEDQLLIFQNKMKDAQNKPPPTGPKHQAQWEIFQIAHTRSRYIYDLYYEKEAISKQLYEWLLKNGYADA
MLIAKWKKQGYEKLCCLRCVQTKETNFNSTCICRVPKAQLKEDQEVQCVNCGCHGCASSD

>KZL68406.1 cell cycle control protein cwf14, partial [Colletotrichum incanum]

PNINSFVIQHTTATRPTFEKTFVYAKPLHEELQPRFYDSPTPKMPPMRSSKRKPPPEGFSDIEDQLLIFQNKMKDAQNKPPPTGPKH QAQWEIFQIAHTRSRYIYDLYYEKEAISKQLYEWLLKNGYADAMLIAKWKKQGYEKLCCLRCVQTKETNFNSTCICRVPKAQLKEDQ EVQCVNCGCHGCASSD

>XP_007603326.1 hypothetical protein CFIO01_11816 [Colletotrichum fioriniae PJ7] >EXF73044.1 hypothetical protein CFIO01_11816 [Colletotrichum fioriniae PJ7] >KXH50976.1 hypothetical protein CSIM01_11656 [Colletotrichum simmondsii] >KXH51668.1 hypothetical protein CNYM01_06791 [Colletotrichum nymphaeae SA-01] >OHE96093.1 hypothetical protein CORC01_08630 [Colletotrichum orchidophilum]

MPPMRSSKRKPPPEGFNDIEDQLLIFQNKMKDAQNKPPPTGPKHQAQWEIFQIAHARSRYIYDLYYEKEAISKQLYEWLLKNGYADA MLIAKWKKQGYEKLCCLRCVQTKETNFNSTCICRVPKAQLKGDQEVQCVACGCHGCASSD

>XP_003018086.1 hypothetical protein TRV_07922 [Trichophyton verrucosum HKI 0517] >EFE37441.1 hypothetical protein TRV_07922 [Trichophyton verrucosum HKI 0517]

MPPIRSARTRKAPPDGFDDIEDTLLEFSNKMKDAENASHEGKKRHEVLWPIFQISHQRSRYIYDLYYEKEAISKKLYDWLLKNGYAD ANLIAKWKKQGYEKLCCLRCIQTKETNFNATCICRVPKAQLKEDQGIQCMQGIACVTALFIFLFFRWSGSGVPLFGFQAMMMEFFYC MCVSAWALYCWFEFVSLAIGFSIFVFLRVAALGDEYCIDTHQIQNMLYMNITLIRLSTIKE

>KDN66531.1 hypothetical protein CSUB01_00976 [Colletotrichum sublineola]

MPPMRSSKRKPPPEGFSDIEDQLLIFQNKMKDAQNKPPPTGPKHQAQWEIFQIAHTRSRYIYDLYYEKEAISKQLYEWLLKNGYADA MLIAKWKKQGYEKLCCLRCVQTKETNFNSTCVCRVPKAQLKEDQDVQCVNCGCHGCASSD

>OQD70516.1 hypothetical protein PENDEC_c023G03315 [Penicillium decumbens]

MPPIRTSRNRKPPPAGFDDIEDTLLEFSNKMKDAENASHEGKKKYEVLWPIFQISHQRSRYIYDLYYEKEAISKQLYDWLLKNNYAD ANLIAKWKKQGYEKLCCLRCVQTKETNFNATCICRVPRAQLKEDQTIQI

>EWC48259.1 BUD31-like protein [Drechslerella stenobrocha 248]

MPPIRTRNKRKPPPDGFEDIEDTLLEFENKMKDAENAPHEGKRRNEMLWPIFHISHQRSRYIYDMYLKEGITRELYDWLLKNKYADP LLIAKWKKQGYEKLCCLKCIQTKETNFGSTCICRVPKARMSKETVVECVSCGCRGCASSD

>KXT09475.1 hypothetical protein AC579_4436 [Pseudocercospora musae]

MPAIRTAKNRKPPPGKKSPYGFDDIEDTLLEFQNKMKDAENASHEGKKKHEMQWPIFQITHQRSRYIYDLYYEKEAISKKLYEWLLK NNYADANLIAKWKKQGYEKLCCLRCIQTKETNFNSTCICRVPREQLKEDQEIQCETSMKGVWAFFFLSKNL

>ERS97760.1 hypothetical protein HMPREF1624_05931 [Sporothrix schenckii ATCC 58251]

MPAIRHASKRKPPPDGFDDIEDDLLIFANKMKDAQSTPTDGLPKHQAQWPIFQISHQRSRYVYELYYEKEAISKQLYDWLLKNGYAD AMLIAKWKKQGYEKLCCLRCVQTKETNFNSTCICRVPKAQLKEDQDIQCVSCGCRGCSSSD

>XP_018162143.1 Cell cycle control protein cwf14 [Colletotrichum higginsianum IMI 349063] >CCF37759.1 hypothetical protein CH063_09016 [Colletotrichum higginsianum] >OBR13626.1 Cell cycle control protein cwf14 [Colletotrichum higginsianum IMI 349063]

MPPVRSSKRKPPPEGFGDIEDQLLIFQNKMKDAQNKPPPTGPKHQAQWEIFQIAHTRSRYIYDLYYEKEAISKQLYEWLLKNGYADA MLIAKWKKQGYEKLCCLRCVQTKETNFNSTCICRVPKAQLKEDQDVQCVSCGCHGCASSD

>OGE49210.1 hypothetical protein PENARI_c023G00850 [Penicillium arizonense]

MPPIRTSRNRKPPPAGFDDIEDTLLEFSNKMKDAENASHEGKKKYEVLWPIFQISHQRSRYIYDLYYEKEAISKQLYDWLLKNGYAD ANLIAKWKKQGYEKLCCLRCVQTKETNFNSTCICRVPKAQLKEDQTIHD

>XP_011128027.1 hypothetical protein AOL_s00215g523 [Arthrobotrys oligospora ATCC 24927] >EGX43787.1 hypothetical protein AOL_s00215g523 [Arthrobotrys oligospora ATCC 24927]

MPPIRTRNKRKPPPDGFDDIEDTLLEFENKMKDAENAPHDGKRRNEVLWPIFQISHQRSRYIYDMYLKEGISRDLYDWLLKNKYADP LLIAKWKKQGYEKLCCLKCIQTKETNFGSTCICRVPKARMTGGNAVECVNCGCHGCASSD

>CCX12087.1 Similar to Cell cycle control protein cwf14; acc. no. O74772 [Pyronema omphalodes CBS 100304]

MPPERHARGYSRKAPPDGFDDIEKTLLEFANRMKDAETAPHEGKKKNEATWDIFRISHQRSKYIYDLYYETNAISKQLYDWLLKNKY
ADANLIAKWKKQGYEKLCCMRCIQAKEHNFNTTCICRVPRARLNDESSGIQCTHCGCSGCASCD

>XP_007743593.1 hypothetical protein A1O5_04799 [Cladophialophora psammophila CBS 110553] >XP_016623904.1 hypothetical protein Z519_02627 [Cladophialophora bantiana CBS 173.52] >EXJ72295.1 hypothetical protein A1O5_04799 [Cladophialophora psammophila CBS 110553] >KIW97235.1 hypothetical protein Z519_02627 [Cladophialophora bantiana CBS 173.52]

MPPVRSRAHKKPPPAGFEDIEDTLLEYANKMRDAQNASHEGKKKHEALWPIFQISHARSRYVYDLYYKREAISKQLYEWLIKNGYAD
GALIAKWKKQGYEKLCCLRCVQTKETNFQSTCICRVPKATLKKDGEAGEGGGGIQCVSCGCRGCASSD

>XP_007726517.1 hypothetical protein A1O1_07458 [Capronia coronata CBS 617.96] >EXJ83831.1 hypothetical protein A1O1_07458 [Capronia coronata CBS 617.96]

MPPIRSRGSRKPPPAGFEDIEDTLLEYSNKMRDAQNASHEGKKKHEAVWPVFQISHARSRYIYDLYYTREAISKELYDWLLKNGYAD
ANLIAKWKKQGYEKLCCLRCVQTKETNFQSTCICRVPKATLKRDDGSGEGAAGIQCVNCGCRGCASSD

>XP_003663306.1 hypothetical protein MYCTH_2305078 [Thermothelomyces thermophila ATCC 42464] >AEO58061.1 hypothetical protein MYCTH_2305078 [Thermothelomyces thermophila ATCC 42464]

MPAIRPASKRKPPPDGFEDIEEDLLIFSNKMKDAQNTPTDNIPKHQAQWPIFQIAHQRSRYVYELYYEKEAISKQLYDWLLKNGYAD
PMLIAKWKKQGYEKLCCLRCIQTKETNFNSTCICRVPRAQLKEDQDVQCVNCGCRGCASTD

>XP_001229811.1 conserved hypothetical protein [Chaetomium globosum CBS 148.51] >EAQ91360.1 conserved hypothetical protein [Chaetomium globosum CBS 148.51]

MPAIRPSSKRKPPPDGFDDIEEDLLIFGNKMKDAQNTPTDNIPKHQAQWPIFQISHQRSRYVYELYYEKEAISRALYDWLLKNGYAD
AMLIAKWKKQGYEKLCCLRCVQTKETNFNSTCVCRVPRAQLKEDQDVQCVSCGCRGCASTD

>XP_016222797.1 hypothetical protein PV10_05785 [Exophiala mesophila] >KIV91223.1 hypothetical protein PV10_05785 [Exophiala mesophila]

MPPVRSRNNRKPPPAGFADIEDTLLEYGNKMRDAENASHDGKKKHEVLWPIFQISHARSRYVYDLYYEREAISKTLYDWLLKNGYAD
ANLIAKWKKQGYEKLCCLRCVQTKETNFAGTCICRVPKASLKRDEGGDQGGGGIQCVNCGCRGCASSD

>XP_014172098.1 cell cycle control protein cwf14 [Grosmannia clavigera kw1407] >EFX02616.1 cell cycle control protein cwf14 [Grosmannia clavigera kw1407]

MPAIRHASKRKPPPDGFTDIEDDLLVFANKMKDAQNAPSDNMPKHQAQWPIFQISHQRSRYIYELYYDKEAISKQLYDWLLKNGYAD
AMLIAKWKKQGYEKLCCLRCVQTKETNFNSTCICRVPKAQLKEDQEIQCVSCGCRGCASSD

>XP_013263230.1 hypothetical protein A1O9_02201 [Exophiala aquamarina CBS 119918] >KEF60640.1 hypothetical protein A1O9_02201 [Exophiala aquamarina CBS 119918]

MPPIRSRNSRKPPPAGFDDIEDTLLEYSNKMRDAQNASHEGKKKHETLWPIFQISHARSRYIYDLYYEREAISKQLYDWLLKNNYAD
MNLIAKWKKQGYEKLCCLRCVQTKETNFAGTCICRVPKAGLKREDGGGDAGGGGGGAGIQCVNCGCRGCASSD

>CZR60046.1 probable G10 protein [Phialocephala subalpina]

MPAIRHASKRKAPPAGFSDIEDDLLIFSNKMKDAENAPTTNVPRHQVHWPIFQISHQRSRYVYELYYEKEAISKELYEWLLKNNYAD
KMLIAKWKKTGYEKLCCLRCVQTKETNFNSTCICRVPKAQMKEDQAIECVSCGCRGCASSD

>XP_009653637.1 cell cycle control protein cwf14 [Verticillium dahliae VdLs.17] >EGY14781.1 cell cycle control protein cwf14 [Verticillium dahliae VdLs.17]

MPPIRHSSKRKPPPDGFSDLEDQLLIFQNKMKDAQNKPPPTGPKHQAQWEIFQISHQRSRYIYDLYYEKEAISKQLYDWLLKNGYAD
AMLIAKWKKQGYEKLCCLRCVQTKETNFNSTCVCRVPKATLKDDQEVQCVSCGCRGCASSD

>XP_008727920.1 hypothetical protein G647_05367 [Cladophialophora carrionii CBS 160.54] >ETI23565.1 hypothetical protein G647_05367 [Cladophialophora carrionii CBS 160.54]

MPPVRARSTKKPPPAGFDDIEDTLLEYSNKMRDAQNASHEGKKKHEVLWPIFQISHARSRYVYDLYYTREAISKQLYEWLIKNGYAD
AALIAKWKKQGYEKLCCLRCVQTKETNFAGTCICRVPKATLKRDGEEGGGGGGGGIQCVNCGCRGCASSD

>CRK30293.1 hypothetical protein BN1708_000828 [Verticillium longisporum]

MPPIRHSSKRKLPPDGFSDLEDQLLIFQNKMKDAQNKPPPTGPKHQAQWEIFQISHQRSRYIYDLYYEKEAISKQLYDWLLKNGYAD AMLIAKWKKQGYEKLCCLRCVQTKETNFNSTCVCRVPKATLKDDQEVQCVSCGCRGCASSD

Saccharomycotina

>ODQ69842.1 hypothetical protein LIPSTDRAFT_183953 [Lipomyces starkeyi NRRL Y-11557]

MPRIKTLRTKKPPEGFAEIEDTLLDFANRLRDIESTSHEGKRKNESLWPVFQITHQRSRYIYDLFYTREAISSELYQWLL

KQGYADGNLIAKWKKQGYEKLCCLRCIQAKENTFNATCICRVPKSKLDKDKVVECVTCGCRGCASSD

>ODV96659.1 hypothetical protein PACTADRAFT_39176 [Pachysolen tannophilus NRRL Y-2460]

MPKLRTSRSKKPPKGFSEIEPTLKEFADKLRDLQTSSGNSKKSLQTKKNESLWPIYQLHHQRSRYIYDLYYEKKAISDEL

YQYLLKNKYADGQLIAKWKKQGYENLCCLGCIATNEKNHGSTCICRVPKAKLKLKSIQCVTCGCRGCASTD

>CDO54379.1 similar to Saccharomyces cerevisiae YCR063W BUD31 Component of the SF3b subcomplex of the U2 snRNP [Geotrichum candidum]

MPAVRTSRSKKAPEGFSEIEDTLLEFENRLKDAEAESSDGKTKNESLWPIYQIHHQRSRYVYDLYYTREKAISTKLYQWL

LKNRYADAKLIAKWRKQGYEQLCCLRCIQTKDNIHGGTCVCRVPHKQLKSEKPVQCVTCGCRGCASTD

>ANZ75757.1 BA75_02019T0 [Komagataella pastoris]

MARPKRPPPEGFQNIEPTLLQFSEKLKEIENTKSKTISKKEALWPIYQVHHQRSRYIYELYYKRKMISKELLTWLLKNKY

ADQNLIAKWRKKGYEKLCCLRCIQSDENNQKNTCICRVPKEQLDKELRCVTCGCKGCASGD

>XP_002492061.1 Protein involved in bud-site selection [Komagataella phaffii GS115] >CAY69781.1 Protein involved in bud-site selection [Komagataella phaffii GS115] >AOA62425.1 GQ67_01201T0 [Komagataella phaffii] >AOA68026.1 GQ68_00188T0 [Komagataella phaffii GS115] >SCV11974.1 U2 snRNP SF3b subcomplex subunit [Komagataella phaffii CBS 7435]

MARPKRPPPEGFQNIEPTLLQFSEKLKEIENTKSKKISKKEALWPIYQVHHQRSRYIYELYYKRKMISKELLTWLLKNKY

ADQNLIAKWRKKGYEKLCCLRCIQSDENNQKNTCICRVPKEQLEKELRCVTCGCRGCASGD

>SCU77973.1 LANO_0A01750g1_1 [Lachancea nothofagi CBS 11611]

MPRIKTKRTKTAPDGFEKIKPTLVDFELRLKEVQDDRASKIGAKSQQGSWDVFQISHERSRYVYDLFYKRKAISKELYDW

LLREKYADKMLIAKWKKRGYEKLCCLKCIQSNESTAQGNTCICRVPRSTLEKNSKDGVVTFTRCVHCGCSGCASSD

>CDR45091.1 CYFA0S16e02058g1_1 [Cyberlindnera fabianii]

MAKIRTSRTKKAPEGFDDIKPTLESFEERLKDAQSVALHKTGRKSESLWEIFRITHERSRYVYDLHYKKKAISKDLYEWL

LKQRYADANLIAKWKKQGYENLCCIKCIQGTENNNGGTCICRVPKATLEKNEKFTFKECINCGCRGCASSD

>CDO93117.1 unnamed protein product [Kluyveromyces dobzhanskii CBS 2104]

MPRIRTNRTKKAPDGFDKIQPTLNELAVQLKEAESEKGSKVSSKNTESTWRVFQLHHERTRYVYSLFYKRKAISKELYDW

LLHEKYADKYLIAKWKKNGYEKLCCLRCIQSEETTNGKTCICRVPRATLEASAAKKEEPVTFQQCVHCGCSGCASTD

>SCV04015.1 LAME_0H15060g1_1 [Lachancea meyersii CBS 8951]

MPRIKTKRTKAAPEGFDKIQPTLADFDLRLKEVHSEQGSKLAAKANEGTWKILQLTHERSRYIFDLFYKRKAISKELYEW

LLREKYADKMLIAKWKKRGYEKLCCLRCIQSSESSSEGHTCICRVPRSVLEKNSKDGVVTFTRCTHCGCSGCASSD

>XP_018223430.1 BUD31-like protein [Saccharomyces eubayanus] >KOH00714.1 BUD31-like protein [Saccharomyces eubayanus]

MPRVKTRRTKPAPDGFDKIKPTLTDFEVQLRDAQRDNTSKLAAKSSEQLWEILQIHHQRSRYIYTLYYKRKAISKDLYDW

LLREKYADKLLIAKWRKTGYEKLCCLRCIQKNETNNGSTCICRVPRAQLEQEAQKKATQVSFHECVHCGCRGCASTD

>CUS23366.1 LAQU0S09e03158g1_1 [Lachancea quebecensis]

MPRIKTKKTKVAPAGFDKIKPTLIDFDIQLKELQDDKASRLAANADQGAWKVFQLSHERSRYVYDLYYKRKAISKDLYEW

LLREKYADKMLIAKWKKKGYEKLCCLRCIQTSETAQGNTCICRVPRATLEKNSKDGIVTFTRCVHCGCSGCASTD

>SCV01493.1 LAMI_0G11848g1_1 [Lachancea mirantina]

MPRIKTKRTKPAPEGFEKVKPTLVDFELQLKEVQSDKASRLSAKAEEGVWKVFQISHERSRYVYNLYYKRHAISRELYEW

LLREKYADKTLIAKWKKKGYEKLCCLRCIQSTETAQGTTCICRVPRSTLEENSKDGKVTFQRCVHCGCSGCASTD

>XP_002551740.1 KLTH0A06512p [Lachancea thermotolerans CBS 6340] >CAR21298.1 KLTH0A06512p [Lachancea thermotolerans CBS 6340]

MPRIKTKNTKATPAGFDKIKPTLIDFDIQLKELQDDKASRLAANADQGAWKVFQLSHERTRYVYDLYYKRKAISKELYEW

LLREKYADKMLIAKWKKKGYEKLCCLRCIQTSETAQGNTCICRVPRATLEKNSKDGVVTFTRCVHCGCSGCASTD

>XP_018987541.1 hypothetical protein BABINDRAFT_10685 [Babjeviella inositovora NRRL Y-12698] >ODQ82213.1 hypothetical protein BABINDRAFT_10685 [Babjeviella inositovora NRRL Y-12698]

MPKLKTSRTKKAPEGFESIQSLLEKFNNKLKAAETKSTASSSVKKNEILWEIYQIHHQRSRYIYDLYYKKEIILKPLYDW

LLKNKYGDINLIAKWRKQGYENLCCLRCIQTKENNHGSTCICRVPKNDLLEEKKIQCITCGCRGCSSTD

>ODQ67486.1 Bud site selection protein 31, partial [Nadsonia fulvescens var. elongata DSM 6958]

MPRIKTNRTKTPPSDFKLVSPTLDEFALRLKEATLSSKALPSSSKNASLWEIHQIYHQRSRYIYDMYYSKEAISSELYQW

ILKNNYADANLIAKWKKQGYEKLCCLRCIQTGDNIHGGTCICRVPRSELKEGHKVSGCVTCGCRGCASTD

>XP_020069215.1 G10 protein [Cyberlindnera jadinii NRRL Y-1542] >CEP21775.1 unnamed protein product [Cyberlindnera jadinii] >ODV72176.1 G10 protein [Cyberlindnera jadinii NRRL Y-1542]

MVRPRTSRAKKAPDGFHNIEPTLNSFEDKLKDAQATALSKTGRKSESLWEIFRITHQKSRYVYELYYKKKAISKELYDWL

LKHRYCDANLIAKWKKQGYENLCCIKCIQGSENNNGGTCICRVPKATLEKNENIKFKECVNCGCRGCASSD

>SCW00322.1 LAFE_0C01596g1_1 [Lachancea fermentati]

MPRIKTSRTKAAPEGFEKIKPTLVDFELQLKEAQGDKASKLAAKADEGAWKVFQICHERSRYVYELFYKRKAISKELYAW

LLREKYADKMLIAKWKKKGYEKLCCLRCIQSTETAHGNTCICRVPRSTLEKNSADGVVAFKRCVHCGCSGCASTD

>XP_003958017.1 hypothetical protein KAFR_0F02860 [Kazachstania africana CBS 2517] >CCF58882.1 hypothetical protein KAFR_0F02860 [Kazachstania africana CBS 2517]

MPRLLTKRTKAAPEGFEKIKPTLTEFELKLKEVGTEKDSKLSSKANENLWKIMQIHHERSRYVYKLYYKRKLISRELYEW

LLKEKYADKHLIAKWRKKGYEKLCCLRCIQAGETNYGNTCICRVPRMQLEADAEKKGLDFTFKQCVHCGCHGCASTD

>XP_020047582.1 G10 protein [Ascoidea rubescens DSM 1968] >ODV61275.1 G10 protein [Ascoidea rubescens DSM 1968]

MPKIRTSRTRKPPSGFEDLEPQLKEFEDELKEIQLTKLKKSVNKNEPLWEIFKIYHKRSRYIYDLYYNRQIISRELYLWL

LKEKFADLHLIAKWKKKGYENLCCLRCIMSNDNNNGFTCICRVPKANLTDDKNSTKCVNCGCRGCASSD

>XP_452593.1 hypothetical protein [Kluyveromyces lactis NRRL Y-1140] >CAH01444.1 KLLA0C08800p [Kluyveromyces lactis]

MPRIRTHATKKAPEGFDKISPTLNEFAIQLKEAESEKGSKLSTKNTESTWQVFQIHHERSRYVYNLFYKRKAISRELYEW

LLREKYADKQLIAKWKKKGYEKLCCLQCIQSNETTNGKTCICRVPRATLEANAAKKKEPVTFKQCIHCGCSGCASSD

>SCU92932.1 LAFA_0F13762g1_1 [Lachancea sp. CBS 6924]

MPRIKTKRTKAAPDGFDKIQPTLIDFDLQLKEVQDQQASKLKAKANEGTWKVLRITHERSRYIYSLYYKRKAISKELYEW

LLREKYADKMLIAKWKKRGYEKLCCLRCIQSTESSSEGNTCICRVPRSVLEKNSEDGVVTFTRCVHCGCAGCASSD

>NP_009990.1 U2 snRNP complex subunit BUD31 [Saccharomyces cerevisiae S288C] >XP_015331864.1 PREDICTED: pre-mRNA-splicing factor BUD31 [Marmota marmota marmota] >P25337.1 RecName: Full=Pre-mRNA-splicing factor BUD31; AltName: Full=Bud site selection protein 31; AltName: Full=Complexed with CEF1 protein 14 >pdb|5LJ3|L Chain L, Structure Of The Core Of The Yeast Spliceosome Immediately After Branching >pdb|5GMK|T Chain T, Cryo-em Structure Of The Catalytic Step I Spliceosome (c Complex) At 3.4 Angstrom Resolution >pdb|5LJ5|L Chain L, Overall Structure Of The Yeast Spliceosome Immediately After Branching. >pdb|5LQW|E Chain E, Yeast Activated Spliceosome >pdb|5MPS|L Chain L, Structure Of A Spliceosome Remodeled For Exon Ligation >pdb|5MQ0|L Chain L, Structure Of A Spliceosome Remodeled For Exon Ligation >pdb|5WSG|T Chain T, Cryo-em Structure Of The Catalytic Step Ii Spliceosome (c* Complex) At 4.0 Angstrom Resolution >CAA42278.1 hypothetical protein [Saccharomyces cerevisiae] >EDN62177.1 bud site selection protein [Saccharomyces cerevisiae YJM789] >EDV09753.1 bud site selection protein BUD31 [Saccharomyces cerevisiae RM11-1a] >EEU05395.1 Bud31p [Saccharomyces cerevisiae JAY291] >CAY78268.1 Bud31p [Saccharomyces cerevisiae EC1118] >DAA07536.1 TPA: U2 snRNP complex subunit BUD31 [Saccharomyces cerevisiae S288C] >EGA59577.1 Bud31p [Saccharomyces cerevisiae FostersB] >EGA63202.1 Bud31p [Saccharomyces cerevisiae FostersO] >EGA75794.1 Bud31p [Saccharomyces cerevisiae AWRI796] >EGA79736.1 Bud31p [Saccharomyces cerevisiae Vin13] >GAA21967.1 K7_Bud31p [Saccharomyces cerevisiae Kyokai no. 7] >EHN08227.1 Bud31p [Saccharomyces cerevisiae x Saccharomyces kudriavzevii VIN7] >EIW11796.1 Bud31p [Saccharomyces cerevisiae CEN.PK113-7D] >EWG87459.1 Bud31p [Saccharomyces cerevisiae R008] >EWG92263.1 Bud31p [Saccharomyces cerevisiae P301] >EWG97296.1 Bud31p [Saccharomyces cerevisiae R103] >EWH19302.1 Bud31p [Saccharomyces cerevisiae P283] >AHV79326.1 BUD31 [synthetic construct] >AHY79735.1 Bud31p [Saccharomyces cerevisiae YJM993] >AJP37486.1 Bud31p [Saccharomyces cerevisiae YJM1078] >AJQ31971.1 Bud31p [Saccharomyces cerevisiae YJM1356] >AJQ32117.1 Bud31p [Saccharomyces cerevisiae YJM1381] >AJQ32261.1 Bud31p [Saccharomyces cerevisiae YJM1383] >AJQ32407.1 Bud31p [Saccharomyces cerevisiae YJM1385] >AJQ32555.1 Bud31p [Saccharomyces cerevisiae YJM1386] >AJQ32701.1 Bud31p [Saccharomyces cerevisiae YJM1387] >AJQ32848.1 Bud31p [Saccharomyces cerevisiae YJM1388] >AJQ32991.1 Bud31p [Saccharomyces cerevisiae YJM1389] >AJQ33134.1 Bud31p [Saccharomyces cerevisiae YJM1399] >AJQ33279.1 Bud31p [Saccharomyces cerevisiae YJM1400] >AJQ33425.1 Bud31p [Saccharomyces cerevisiae YJM1401] >AJQ33573.1 Bud31p [Saccharomyces cerevisiae YJM1402] >AJQ33717.1 Bud31p [Saccharomyces cerevisiae YJM1415] >AJQ33856.1 Bud31p [Saccharomyces cerevisiae YJM1417] >AJQ34006.1 Bud31p [Saccharomyces cerevisiae YJM1418] >AJQ34152.1 Bud31p [Saccharomyces cerevisiae YJM1419] >AJQ34299.1 Bud31p [Saccharomyces cerevisiae YJM1433] >AJQ34449.1 Bud31p [Saccharomyces cerevisiae YJM1434] >AJQ34596.1 Bud31p [Saccharomyces cerevisiae YJM1439] >AJQ34892.1 Bud31p [Saccharomyces cerevisiae YJM1444] >AJQ35036.1 Bud31p [Saccharomyces cerevisiae YJM1447] >AJQ35180.1 Bud31p [Saccharomyces cerevisiae YJM1450] >AJQ35329.1 Bud31p [Saccharomyces cerevisiae YJM451] >AJQ35475.1 Bud31p [Saccharomyces cerevisiae YJM453] >AJQ35619.1 Bud31p [Saccharomyces cerevisiae YJM456] >AJQ35764.1 Bud31p [Saccharomyces cerevisiae YJM470] >AJQ35909.1 Bud31p [Saccharomyces cerevisiae YJM541] >AJQ36056.1 Bud31p [Saccharomyces cerevisiae YJM554] >AJQ36200.1 Bud31p [Saccharomyces cerevisiae YJM555] >AJQ36347.1 Bud31p [Saccharomyces cerevisiae YJM627] >AJQ36489.1 Bud31p [Saccharomyces cerevisiae YJM681] >AJQ36636.1 Bud31p [Saccharomyces cerevisiae YJM682] >AJQ36782.1 Bud31p [Saccharomyces cerevisiae YJM683] >AJQ36929.1 Bud31p [Saccharomyces cerevisiae YJM689] >AJQ37076.1 Bud31p [Saccharomyces cerevisiae YJM693] >AJQ37219.1 Bud31p [Saccharomyces cerevisiae YJM969] >AJQ37368.1 Bud31p [Saccharomyces cerevisiae YJM972] >AJQ37517.1 Bud31p [Saccharomyces cerevisiae YJM975] >AJQ37657.1 Bud31p [Saccharomyces cerevisiae YJM978] >AJQ37795.1 Bud31p [Saccharomyces cerevisiae YJM981] >AJQ37944.1 Bud31p [Saccharomyces cerevisiae YJM984] >AJQ38093.1 Bud31p [Saccharomyces cerevisiae YJM987] >AJQ38242.1 Bud31p [Saccharomyces cerevisiae YJM990] >AJQ38391.1 Bud31p [Saccharomyces cerevisiae YJM996] >AJQ38537.1 Bud31p [Saccharomyces cerevisiae YJM1083] >AJQ38682.1 Bud31p [Saccharomyces cerevisiae YJM1129] >AJQ38832.1 Bud31p [Saccharomyces cerevisiae YJM1133] >AJQ38970.1 Bud31p [Saccharomyces cerevisiae YJM1190] >AJQ39113.1 Bud31p [Saccharomyces cerevisiae YJM1199] >AJQ39262.1 Bud31p [Saccharomyces cerevisiae YJM1202]

>AJQ39410.1 Bud31p [Saccharomyces cerevisiae YJM1208] >AJQ39543.1 Bud31p [Saccharomyces cerevisiae YJM1242] >AJQ39691.1 Bud31p [Saccharomyces cerevisiae YJM1244] >AJQ39838.1 Bud31p [Saccharomyces cerevisiae YJM1248] >AJQ39988.1 Bud31p [Saccharomyces cerevisiae YJM1250] >AJQ40136.1 Bud31p [Saccharomyces cerevisiae YJM1252] >AJQ40282.1 Bud31p [Saccharomyces cerevisiae YJM1273] >AJQ40424.1 Bud31p [Saccharomyces cerevisiae YJM1304] >AJQ40568.1 Bud31p [Saccharomyces cerevisiae YJM1307] >AJQ40712.1 Bud31p [Saccharomyces cerevisiae YJM1311] >AJQ40851.1 Bud31p [Saccharomyces cerevisiae YJM1326] >AJQ40999.1 Bud31p [Saccharomyces cerevisiae YJM1332] >AJQ41146.1 Bud31p [Saccharomyces cerevisiae YJM1336] >AJQ41291.1 Bud31p [Saccharomyces cerevisiae YJM1338] >AJQ41434.1 Bud31p [Saccharomyces cerevisiae YJM1341] >AJQ41727.1 Bud31p [Saccharomyces cerevisiae YJM1355] >AJQ41873.1 Bud31p [Saccharomyces cerevisiae YJM1460] >AJQ42017.1 Bud31p [Saccharomyces cerevisiae YJM1463] >AJQ42152.1 Bud31p [Saccharomyces cerevisiae YJM1477] >AJQ42293.1 Bud31p [Saccharomyces cerevisiae YJM1478] >AJQ42437.1 Bud31p [Saccharomyces cerevisiae YJM1479] >AJQ42582.1 Bud31p [Saccharomyces cerevisiae YJM1526] >AJQ42731.1 Bud31p [Saccharomyces cerevisiae YJM1527] >AJQ42871.1 Bud31p [Saccharomyces cerevisiae YJM1549] >AJQ43018.1 Bud31p [Saccharomyces cerevisiae YJM1573] >AJQ43162.1 Bud31p [Saccharomyces cerevisiae YJM1574] >AJQ43308.1 Bud31p [Saccharomyces cerevisiae YJM1592] >AJQ43451.1 Bud31p [Saccharomyces cerevisiae YJM1615] >AJQ43594.1 Bud31p [Saccharomyces cerevisiae YJM189] >AJQ43738.1 Bud31p [Saccharomyces cerevisiae YJM193] >AJQ43882.1 Bud31p [Saccharomyces cerevisiae YJM195] >AJQ44020.1 Bud31p [Saccharomyces cerevisiae YJM244] >AJQ44166.1 Bud31p [Saccharomyces cerevisiae YJM248] >AJQ44309.1 Bud31p [Saccharomyces cerevisiae YJM270] >AJQ44456.1 Bud31p [Saccharomyces cerevisiae YJM271] >AJQ44597.1 Bud31p [Saccharomyces cerevisiae YJM320] >AJQ44744.1 Bud31p [Saccharomyces cerevisiae YJM326] >AJQ44890.1 Bud31p [Saccharomyces cerevisiae YJM428] >AJQ45037.1 Bud31p [Saccharomyces cerevisiae YJM450] >KOH52070.1 BUD31p Component of the SF3b subcomplex of the U2 snRNP [Saccharomyces sp. 'boulardii'] >KQC45222.1 Component of the SF3b subcomplex of the U2 snRNP [Saccharomyces sp. 'boulardii'] >KZV12910.1 BUD31 [Saccharomyces cerevisiae]

MPRIKTRRSKPAPDGFEKIKPTLTDFEIQLRDAQKDKSSKLAAKSNEQLWEIMQLHHQRSRYIYTLYYKRKAISKDLYDW

LIKEKYADKLLIAKWRKTGYEKLCCLRCIQKNETNNGSTCICRVPRAQLEEEARKKGTQVSFHQCVHCGCRGCASTD

>AJQ34746.1 Bud31p [Saccharomyces cerevisiae YJM1443]

MPRIKTRRSKPAPDGFEKIKPTLTDFEIQLRDAQKDKSFKLAAKSNEQLWEIMQLHHQRSRYIYTLYYKRKAISKDLYDW

LIKEKYADKLLIAKWRKTGYEKLCCLRCIQKNETNNGSTCICRVPRAQLEEEARKKGTQVSFHQCVHCGCRGCASTD

>XP_003676394.1 hypothetical protein NCAS_0D04520 [Naumovozyma castellii CBS 4309] >CCC70033.1 hypothetical protein NCAS_0D04520 [Naumovozyma castellii CBS 4309]

MPRIKTNRTNQAPDGFDKIKPTLDDFELRLREIHEKKDSRLSTKANENLWKIIQINHERSHYIYKLFYKRKAISRELYDW

LLKEKYADKFLIAKWKKKGYEKLCCLRCIQSDETNRGKTCICRVPRIQLEEDAESRGTEVTFQQCVHCGCRGCASTD

>EHN03434.1 Bud31p [Saccharomyces cerevisiae x Saccharomyces kudriavzevii VIN7] >EJT44203.1 BUD31-like protein [Saccharomyces kudriavzevii IFO 1802]

MPRVKTRRTKPAPDGFDKIKPTLTDFAIQLRDAQQDKSSKLAAKSTEQLWDIMQIHHQRSRYIYTLYYKRKAISKDLYQW

LVKEKYADKLLIAKWRKTGYEKLCCLRCIQKNETNNGGTCICRVPRAQLEEEAHKKGAQVSFHQCVHCGCRGCASTD

>XP_003671854.1 hypothetical protein NDAI_0I00420 [Naumovozyma dairenensis CBS 421] >CCD26611.1 hypothetical protein NDAI_0I00420 [Naumovozyma dairenensis CBS 421]

MPRIRTKRTRPPPEGFAKIEPTLEDFEERLKEINKQKDSKLQTKSNENLWKIMQINHERSRYIYKLFYSRKAISRELYDW

LLKEKYADKFLIAKWKKKGYEKLCCLKCIQSDETNSNKTCICRVPRAQLEHDAESKNMSVTFQQCVHCGCRGCASTD

>ONH80197.1 Pre-mRNA-splicing factor BUD31 [Saccharomyces cerevisiae]

MPRIKTRRSKPAPDGFEKIKPTLTDFEIQLRDAQKDKSSKLAAKSNEQLWEIMQLYHQRSRYIYTLYYKRKAISKDLYDW

LIKEKYADKLLIAKWRKTGYEKLCCLRCIQKNETNNGSTCICRVPRAQLEEEARKKGTQVSFHQCVHCGCRGCASTD

>pdb|2MY1|A Chain A, Solution Structure Of Bud31p

GGSPRIKTRRSKPAPDGFEKIKPTLTDFEIQLRDAQKDKSSKLAAKSNEQLWEIMQLHHQRSRYIYTLYYKRKAISKDLY

DWLIKEKYADKLLIAKWRKTGYEKLCCLRCIQKNETNNGSTCICRVPRAQLEEEARKKGTQVSFHQCVHCGCRGCASTD

>AJQ41584.1 Bud31p [Saccharomyces cerevisiae YJM1342]

MSRIKTRRSKPAPDGFEKIKPTLTDFEIQLRDAQKDKSSKLAAKSNEQLWEIMQLHHQRSRYIYTLYYKRKAISKDLYDW

LIKEKYADKLLIAKWRKTGYEKLCCLRCIQKNETNNGSTCICRVPRAQLEEETRKKGTQVSFHQCVHCGCRGCASTD

>XP_501048.1 YALI0B18238p [Yarrowia lipolytica CLIB122] >CAG83301.1 YALI0B18238p [Yarrowia lipolytica CLIB122] >AOW01876.1 hypothetical protein YALI1_B23609g [Yarrowia lipolytica] >SEI30697.1 YALIA101S01e05446g1_1 [Yarrowia lipolytica]

MVKIRTSRSKAPPAGFDDISDILQEFGDKLKDAQNAPTEGKKKNQLLWDIYRIHHQRSRYVYELYYKKEAITKELYAYLL

KKGYADQNLIAKWRKQGYENLCCLRCIQGKENIHEGTCICRVPRKDIKDDKPVECVTCGCRGCASSD

>CEP61125.1 LALA0S02e07272g1_1 [Lachancea lanzarotensis]

MPRIKTKRTKAAPDGFDKIQSTLADFDLQLKEVQDQDASKLKAKANEGTWKVLRITHERSRYIYSLYYKRKAISKELYTW

LLREKYADKMLIAKWKKRGYEKLCCLQCIQSAESNSEGNTCICRVPRSVLEKNSKDGVVTFTRCVHCGCTGCASTD

>XP_019040207.1 hypothetical protein WICANDRAFT_83270 [Wickerhamomyces anomalus NRRL Y-366-8] >XP_019040299.1 hypothetical protein WICANDRAFT_77738 [Wickerhamomyces anomalus NRRL Y-366-8] >ODQ61000.1 hypothetical protein WICANDRAFT_83270 [Wickerhamomyces anomalus NRRL Y-366-8] >ODQ61092.1 hypothetical protein WICANDRAFT_77738 [Wickerhamomyces anomalus NRRL Y-366-8]

MAKIKSSRSKQPPDGYDSIKPTLDEFDNKMKDVQSKALTKSGRKSEALWDIFRVSHQRSRYVYEMYYKKKLISKELYDWC

LKNRKIDANLIAKWKKQGYENLCCIKCIQGSENNNGGTCICRVPRATLEKHENVKFSECINCGCRGCASTD

>XP_011276158.1 hypothetical protein BN7_1545 [Wickerhamomyces ciferrii] >CCH42006.1 hypothetical protein BN7_1545 [Wickerhamomyces ciferrii]

MPKIRSTKSKKAPGGYDSIKPTLDEFDDKMKDVQSKALTKTGKKNEALWDIFRISHQRSRYIYEMYYKKKVISKDLYDWV

LKNRKINANLIAKWKKQGYEHLCCVKCIQGNESNNGGTCICRVPRATLEKNEKIKFTQCVNCGCRGCASTD

>BAO39794.1 bud site selection protein 31 [Kluyveromyces marxianus DMKU3-1042] >BAP71278.1 bud site selection protein 31 [Kluyveromyces marxianus]

MPRIKTNRTKKAPEGFDKISNTLDEFAIQLKEVQNQKGSKLSSKDTESTWRVFQIHHERSRYIYTLYYKRKAISKELYEW

LLREKYADKLLIAKWKKKGYENLCCLNCIQSDKTVNGKTCICRVPRATLKDTAAKNNEPVTFQQCVHCGCSGCASTD

>XP_004182411.1 hypothetical protein TBLA_0I02340 [Tetrapisispora blattae CBS 6284] >CCH62892.1 hypothetical protein TBLA_0I02340 [Tetrapisispora blattae CBS 6284]

MVRIRTKNSSKAPAGFDKIEPSLLNFELELKEIHDKKTSKLGAKSNENLWDIMRVNHERSRYIYSLYYKRKAISKELYAW

LLKEKYADKFLIAKWRKQGYEKLCCVRCIQTNETAHGGTCICRVPRAQLEKDASENSVAVTFQQCVHCGCRGCASND

>SCU89168.1 LADA_0E14026g1_1 [Lachancea dasiensis CBS 10888]

MPRIKTKRTKAAPEGFEQIKPTLTDFDHRLRKLQDNGASRMAAKANEGSWKVFQLTNERSRYIYDLYFKRKAISKELYDW

LLRERYADKMLISKWKKRGYEKLCCLKCIQSSESATQGNTCICRVPRATLVKNSSDGVVTFTRCVHCGCAGCSSSD

>XP_015469889.1 hypothetical protein AC631_00422 [Debaryomyces fabryi] >KSA03787.1 hypothetical protein AC631_00422 [Debaryomyces fabryi]

MAKISSSRRRKQPPEGYSKIEPTLAKLLAKSREAQTKSIKTENKNQALWPIIQVNHQINRYIYSLYYERELISEELYNWL

LQQKYANKNLIAKWKKQGYEKLCCLNCIMTSEKNHGTTCICRVPKTTLVKNDRSERVECITCGCKGCASTD

>XP_460855.2 DEHA2F11286p [Debaryomyces hansenii CBS767] >CAG89200.2 DEHA2F11286p [Debaryomyces hansenii CBS767]

MAKISSSKKNKQPPEGYSKIEPTLSKLLVKSREAQTKSIKTENKNQSLWPIIQINHQINRYIYSLYYERESISQELYNWL

LQQKYANKNLIAKWKKQGYEKLCCLNCIMTNEKNHGTTCICRVPKTTLVKNDRSERVECITCGCRGCASTD

>EJS44477.1 bud31p [Saccharomyces arboricola H-6]

MARVKTRRTKPAPEGFEKIKPTLTDFDIQLRDAQRDKPSKLASKSNEQLWEIMQIHHQRSRYIYTLYYKRKAISKDLYEW

LVREKYADKLLIAKWRKSGYEKLCCLRCIQKNETNNGSSCICRVPRAQLEEEAHKKDTQVSFHQCVHCGCRGCASTD

>CCE84585.1 Piso0_004133 [Millerozyma farinosa CBS 7064] >CCE83554.1 Piso0_004133 [Millerozyma farinosa CBS 7064]

MAKINRRKLKKPPEGYEKIEPTLVKLQEKLKQVQRSSIKTENKHSSLWPVFQVDHQINRYIYSLYYERKQISSELYEWLL

QQKYANKDLIAKWKKQGYEKLCCLSCIMVDEKNHKNTCVCRVPKSTLKENNDSPVECITCGCKGCASTD

>XP_003679288.1 hypothetical protein TDEL_0A07450 [Torulaspora delbrueckii] >CCE90077.1 hypothetical protein TDEL_0A07450 [Torulaspora delbrueckii]

MPRIRTNKKSAPEGFAKIEPTLREFELQLREVEQSKSSKLSSRANEELWQILRICHERSRYVYTLFYKRHAISKQLYEWL

LREKYADKLLIAKWKKQGYEKLCCMRCIQTSESAHGNSCICRVPRAQLELEAQKAGKAVGFQQCVHCGCRGCASTD

>XP_001646631.1 hypothetical protein Kpol_1028p48 [Vanderwaltozyma polyspora DSM 70294] >EDO18773.1 hypothetical protein Kpol_1028p48 [Vanderwaltozyma polyspora DSM 70294]

MPRIKTNRTRKAPDGFDKIEETLREFEIQLKEIQNKKTSKLSANSKENEWEIMRINNERSRYVYSLFYKRKAISRDLYEW

LLKEKYADKYLIAKWKRKGYEKLCCIRCIQTDETIQGKTCICRVPRIQLENESSRQENKVTFQQCVHCGCSGCSSTD

>CDF91341.1 ZYBA0S11-00628g1_1 [Zygosaccharomyces bailii CLIB 213] >CDH17231.1 probable Pre-mRNA-splicing factor BUD31 [Zygosaccharomyces bailii ISA1307] >AQZ18219.1 BUD31 (YCR063W) [Zygosaccharomyces parabailii] >SJM86955.1 probable Pre-mRNA-splicing factor BUD31 [Zygosaccharomyces bailii]

MVSKAPSGFEKVKPTLDEFESQLREVHGAKTSKVSAKKNEHLWQVLRIHHERSRYIYHLFYKRRAISKDLYEWLLRQGIA

DRQLIAKWKKRGYEKLCCLQCIQRSESTHGSTCICRVPRAQLERDDTAFHQCVHCGCHGCASTD

>CDH10984.1 probable Pre-mRNA-splicing factor BUD31 [Zygosaccharomyces bailii ISA1307] >AQZ13753.1 BUD31 (YCR063W) [Zygosaccharomyces parabailii]

MVSKAPPGFEKVKPTLDEFESQLREVHGAKTSKVSAKKNEHLWQVLRIHHERSRYIYHLFYKRRAISKDLYEWLLRQGIA

DRQLIAKWKKRGYEKLCCLQCIQRSESTHGSTCICRVPRAQLERDDTAFHQCVHCGCHGCASTD

>OWB65180.1 hypothetical protein B5S30_g504 [[Candida] boidinii] >OWB85070.1 hypothetical protein B5S33_g3727 [[Candida] boidinii]

MPKIRTIKKYRSNPPEGFQKIEPILIKFNNKLKEAESKNIKLNRPKNESLWEIYKLNHQRSRYIYDLYYKKKIISKILYD

WLLKNKFGDGDLIAKWKKQGYENLCCVKCINVSESNQGTTCICRVPKATLKKNLAITAAGENNDDTGDDKLGKEKDIKCM

TCGCRGCASSD

>OWB55511.1 hypothetical protein B5S28_g1383 [[Candida] boidinii] >OWB60400.1 hypothetical protein B5S29_g1274 [[Candida] boidinii] >OWB73454.1 hypothetical protein B5S31_g3196 [[Candida] boidinii] >OWB77211.1 hypothetical protein B5S32_g1372 [[Candida] boidinii]

MPKIRTIKKYRSNPPEGFQKIEPILIKFNNKLKEAESKNIKLNRPKNESLWEIYKLNHQRSRYIYDLYYKKKIISKILYD

WLLKNKFGDGDLIAKWKKQGYENLCCIKCINVSESNQGTTCICRVPKATLNKNLAITAIGDSNDDDTSDETSVKLGKEKD

IKCMTCGCRGCASSD

>OUM50936.1 hypothetical protein BVG19_g17 [[Candida] boidinii] >OWB52283.1 hypothetical protein B5S27_g3856 [[Candida] boidinii]

MPKIRTIKKYRSNPPEGFQKIEPILIKFNNKLKEAESKNIKLNRPRNESLWEIYRLNHQRSRYIYDLYYKKKIISKILYD

WLLKNKFGDGDLIAKWKKQGYENLCCVKCINVSESNQGTTCICRVPKATLKKNLAITAAGENNDDTGDDKLGKEKDIKCM

TCGCRGCASSD

>XP_001484229.1 hypothetical protein PGUG_03610 [Meyerozyma guilliermondii ATCC 6260] >EDK39512.1 hypothetical protein PGUG_03610 [Meyerozyma guilliermondii ATCC 6260]

MHLSFSLIMPRARRNDKPPEGYSKIEPTLNKLLAKLKDAQSASIKTENKKQALWPVFRLNHQISRYIYTMYYDRKVISKE

LYEWLLKQKFCNANLIAKWKKQGYENLCCINCIMTNETNHGSTCICRVPKSNLEDDEGKTIECITCGCRGCASSD

>XP_003685456.1 hypothetical protein TPHA_0D03890 [Tetrapisispora phaffii CBS 4417] >CCE63022.1 hypothetical protein TPHA_0D03890 [Tetrapisispora phaffii CBS 4417]

MPRINLKTAKNAPKGFEDIKETLQEFQEELDDIQSSEKSSKLAARANVSLWEIMRIHNERSRYVYNLFYKRKAISRELYE

WLLKNKYGDKYLISKWKKKGYEKLCCLRCIQSAETVHGTTCICRVPRAQLERNAEKDGDSKVSFTQCVHCGCHGCSSTD

>AGO10041.1 AaceriAAR051Cp [Saccharomycetaceae sp. 'Ashbya aceri']

MPRSPRSPPPAGFDKVEPTLAAFAQQLRDLQSASAPRGPRASASAWPVFRVAHERSRYVYTMYHRRRAISRDLYDWLLRH

RYADRYLIAKWRKQGYEKLCCLRCIQPGESQYGHTCICRVPRAALEQQSGADFEQCTRCGCRGCASTD

>CCK72074.1 hypothetical protein KNAG_0I02900 [Kazachstania naganishii CBS 8797]

MVLPHHKDVSKAAPDGYEKLAPTFKVFAQRLSDVHNEKESKLSTKANEKLWKIMQIHHERSLYVFKLYYKRKLISRDLYE

WLLKRKLADRNLIAKWRKKGYEKLCCLRCIQSDESQHGTTCICRVPRAQLEEDALRKGTQVSFKQCVHCGCHGCASSN

>XP_006689802.1 G10 protein [[Candida] tenuis ATCC 10573] >EGV60588.1 G10 protein [[Candida] tenuis ATCC 10573]

MPKVKSSKRGGKPPEGYTKIEPTISKLVQKLKDAQTQTEKHSVWKIIQINHQISRYVYTMHYNRKLIDKPLYEWLLKQKY

VDANLIAKWKKQGYEKLCCLNCIRKEDNNFGSSCICRVPKQDLSDDKPVECVKCGCKGCSSTD

>NP_982592.2 AAR051Cp [Eremothecium gossypii ATCC 10895] >AAS50416.2 AAR051Cp [Ashbya gossypii ATCC 10895] >AEY94702.1 FAAR051Cp [Eremothecium gossypii FDAG1]

MPRPPRSPPPAGFDKVEPTLAAFAEQLRDLQGVSAPRGPRASAAAWPVFRVTHERSRYVYTMYHRRRAISRALYDWLLRH

RYADRYLIAKWRKQGYEKLCCLRCIQPGESQYGHTCICRVPRAALELQSGAAFEQCTRCGCRGCASTD

>XP_447862.1 hypothetical protein [Candida glabrata CBS 138] >CAG60811.1 unnamed protein product [[Candida] glabrata] >KTA95897.1 Pre-mRNA-splicing factor BUD31 [[Candida] glabrata] >KTA98323.1 Pre-mRNA-splicing factor BUD31 [[Candida] glabrata] >KTB06083.1 Pre-mRNA-splicing factor BUD31 [[Candida] glabrata] >KTB19156.1 Pre-mRNA-splicing factor BUD31 [[Candida] glabrata] >KTB20435.1 Pre-mRNA-splicing factor BUD31 [[Candida] glabrata] >OXB42012.1 hypothetical protein B1J91_J03630g [[Candida] glabrata] >OXB47311.1 hypothetical protein B1J92_J03630g [[Candida] glabrata]

MKVKGYERVEPVLAEFERRLKEIGKDKSKSLGKGQGKEDLWRIVQIHSERSRYVYTMYYKRRAISRELYEWLLKKKVADR

RLIAKWRKRGYEKLCCLQCVQQSETNHGSTCICRVPRLQLEAEAEKKGVPVSFKECVNCGCHGCASTD

>XP_001387098.2 predicted protein [Scheffersomyces stipitis CBS 6054] >EAZ63075.2 predicted protein [Scheffersomyces stipitis CBS 6054]

MAKLAKKIKKSDPAPDGYSKIEPTLKKLQARMKEAQTSSGVSDKTKSKTQSLWIIYQLNHQISRYVYDMYYKKKLISREL

YDWLLLQSYVNSELIAKWKKQGYEKLCCVHCILVSDKNHKNPCICRVPKAKLLENNESEDKIKNLQCVTCGCRGCASTD

>SGZ49291.1 CIC11C00000001316 [[Candida] intermedia]

MPKASKSKKPPQGYEIVEPTLTKLLAKLKEAQKSSIKSETKTSSLWPILKLNHQISRYVYLMYYERKLITREVYDYLLKQ

KYVNGDLIAKWKKQGYEHLCCVKCIIRKDTNHGSTCICRVPKSEMGDRNKEGCVTCGCRGCSSTD

>ODV85057.1 hypothetical protein CANARDRAFT_23607 [Candida arabinofermentans NRRL YB-2248]

MPKVPSTNSKFKKKPPHGFSKIEPTLTKFSQKLKLAESKPLKLNTPKHESLWEIVQLNHQRSRYIYELYYKKKIISKELY

DWLIKNKFGDLELIAKWKKQGYENLCCLKCMLGKENNHGTTCICRVPKSSLKNTITGDGEVDNDNDNDNGHGKERDIRCV

NCGCRGCASGDL

>XP_003645027.1 Hypothetical protein Ecym_2488 [Eremothecium cymbalariae DBVPG#7215] >AET38210.1 Hypothetical protein Ecym_2488 [Eremothecium cymbalariae DBVPG#7215]

MKHRSPYGIYSIDSLLHLCVSVPTRIMKRTRTAGRTHSTVPPPGFENVKETLDDFDRQLKELQTDSAKASRLSARANEPA

WQVFRITHERSRYIYNLFYRRKAISRQLYRWLLNNRYADRHLIAKWKKRGYEKLCCIPCIQQTETQYGSTCICRVPRATL

EKNSVDGVTTFKNCSHCGCSGCASTD

>XP_020064630.1 Bud site selection protein 31 [Candida tanzawaensis NRRL Y-17324] >ODV79508.1 Bud site selection protein 31 [Candida tanzawaensis NRRL Y-17324]

MPKASRIKKNAPPPAGYDKVSPTLTKLQAKLKQAQVAQSVRTGSSASTKQLSLWPIMQINHQISRYVYDMYYKRKLISRE

LYDWLLVQSYVNGDLIAKWKKQGYEHLCCVQCIMVQDKNHKNACVCRVPKATLLKNGDEESTKAVECVTCGCRGCASTD

>XP_002619084.1 hypothetical protein CLUG_00243 [Clavispora lusitaniae ATCC 42720] >EEQ36120.1 hypothetical protein CLUG_00243 [Clavispora lusitaniae ATCC 42720] >OVF08356.1 putative U2 snRNP complex subunit [Clavispora lusitaniae]

MPKVSNKKTPPEGYDRIEPTLTKLREKLKDAQKASLKTETKNTSLWPIFKLNHQISRYVYMMYYERKLISRELYDYLLRQ

KYVNADLIAKWKKQGYEKLCCVNCIIVNEKNHETTCICRVPRSELKENRNKDGCVTCGCRGCASTD

>XP_017989693.1 HHL073Cp [Eremothecium sinecaudum] >AMD22697.1 HHL073Cp [Eremothecium sinecaudum]

MHENCKAILLLILWSCEKQPTTRTPNYPMQARQVPVPPKNAPPAGYEKIAKTIEHYEQQLKELQSDTAPTALSTRADEQT

WKIFRIINELSRYIYSLYYKRRAISKELYNWLLHHRYGDKNLIAKWKKQGYEKLCCIRCIQSTETQYGTTCICRVPRAVL

EKNSSNGVVTFKNCVHCGCNGCASTD

>GAV48950.1 hypothetical protein ZYGR_0N03550 [Zygosaccharomyces rouxii]

MPTAPPPGFEKIKPALDEFESQLRQIHHAKTSRIAAKKDENLWEILRISNERSRYIFNLFYKRKAISRDLYEWLLKNRLA

DRQLIAKWKKKGYEKLCCLKCIQRKETNHGNVCICRIPRAQLLEKDRNTFHQCNNCGCHGCSSTD

>XP_018712837.1 G10 protein [Metschnikowia bicuspidata var. bicuspidata NRRL YB-4993] >OBA22341.1 G10 protein [Metschnikowia bicuspidata var. bicuspidata NRRL YB-4993]

MPKAPRSKKPPADYEKVEPTLTKLLAKLKEAQSTSLQSSTANKHSSLWPILKLNHQISRYVYSMYYQRKAISKELYDYLI

RQKYVDADLIAKWKKQGYENLCCVGCIVVSEKNHGTTCICRVPRADVDGRNQDGCVTCGCRGCFSSD

>XP_020074631.1 G10 protein [Hyphopichia burtonii NRRL Y-1933] >ODV65564.1 G10 protein [Hyphopichia burtonii NRRL Y-1933]

MPKASKKSKIPEGFDKIEPTLNKLLAKLKESQAKSLKTTNKNESLWPIYQINHQISRYVYSLYYERKTILSELYQYLLKQ

KYVNKDLIAKWKKQGYEKLCCINCIIVNEKNHENTCICRVPRKQLEDDGKDFDRCITCGCKGCSSID

>ODV90452.1 hypothetical protein CANCADRAFT_2181 [Tortispora caseinolytica NRRL Y-17796]

MTKSARKSRRPPPKGYDEVKPTLDAFAERIEELSGVSSEETTWKMFQLLHQRSRYIYEMYYRKHAISKELYDWLLKARLG

DPLLIAKWKKQGYENLCCLRCIDTGSIGGKTTCICRVPKGSMTATDDDVKTIRCRPCGCRGCASGD

>GAV53862.1 hypothetical protein ZYGR_0AK03640 [Zygosaccharomyces rouxii]

MPTAPPPGLEKIKPALDEFESQLRQIHHAKTSKIAAKKDEKLWEILRISNERSRYIFNLFYKRKAISRDLYEWLLKNRLA

DRQLIAKWKKKGYEKLCCLKCIQRKETNHGNVCICRIPRAHLLERDRNTFRQCNNCGCHGCSSTD

>XP_002496801.1 ZYRO0D08448p [Zygosaccharomyces rouxii] >CAQ43589.1 Bud site selection protein 31 [Zygosaccharomyces rouxii] >CAR27868.1 ZYRO0D08448p [Zygosaccharomyces rouxii]

MPTAPPPGFEKIKPALDEFESQLRQIHHAKTSRIAAKKDENPWEILRISNERSRYIFNLFYKRKAISRDLYEWLLKNRLA

DRQLIAKWKKKGYEKLCCLKCIQRKETNHGNVCICRIPRAQLLEKDRNTFHQCNNCGCHGCSSTD

>XP_007377036.1 hypothetical protein SPAPADRAFT_56934 [Spathaspora passalidarum NRRL Y-27907] >EGW31003.1 hypothetical protein SPAPADRAFT_56934 [Spathaspora passalidarum NRRL Y-27907]

MPKIKKVKKNAPPPAGYSKLEPTLTKYQAKLKQAQKVDTTTNKHASLWKIYQIDHQISRYVYDMYVNKRISRELYDWLLL

QSYVNKDLIAKWKKPGYEKLCCVSCIMEKNHGGTCICRVPKVKLLENDNEDKVKTECITCGCKGCASTD

>EGA87768.1 Bud31p [Saccharomyces cerevisiae VL3]

MQLHHQRSRYIYTLYYKRKAISKDLYDWLIKEKYADKLLIAKWRKTGYEKLCCLRCIQKNETNNGSTCICRVPRAQLEEE

ARKKGTQVSFHQCVHCGCRGCASTD

>XP_018165859.1 hypothetical protein QG37_07624 [[Candida] auris] >KND96135.1 hypothetical protein QG37_07624 [[Candida] auris]

MPKANKRKIPEDFDKVKPTIEKLQLKLKEAQRKSIKTETKQTSLWPILKLDHQISRYVYSMYYEKKLISRELYDYLLRQK

YVNADLIAKWKKQGYEKLCCVNCIIAKEKNHGSTCICRVPRHQLKDDQEHPEGCVTCGCQGCASTNKDD

>XP_020546599.1 Protein BUD31 [Pichia kudriavzevii] >KGK39531.1 hypothetical protein JL09_g1278 [Pichia kudriavzevii] >ONH77662.1 Protein BUD31 [Pichia kudriavzevii] >OUT21969.1 hypothetical protein CAS74_002953 [Pichia kudriavzevii]

MEIYQQLGNSTQSLRNRTEYGQQNNATTGYAFDGNLSAGANDIRQGEMTAGKNNKITSNPVKISKRSSPPKGFLKIQPTL

KKFEDKLKELESEEHSSARPKHELHWDLHRINHQKSRYVYSLYYQRRLIDKQLYLWLLKHRFANGQLIAKWRKQGYETLC

CLECIGETVCVCRVPKSVRKTDPDFRCVKCGCRGCASSD

>XP_019019720.1 hypothetical protein PICMEDRAFT_30177 [Pichia membranifaciens NRRL Y-2026] >ODQ48607.1 hypothetical protein PICMEDRAFT_30177 [Pichia membranifaciens NRRL Y-2026]

MGTAGDGKPNGKSIRIPPRSQPPEGFEKILPTLKKFQDKMQTLESKEVNPSRPKHELYWDIHRVDHQKSRYIYSLYFQRK

LISQPLYIWLVKHKFVNGELIAKWRKRGYENLCCLQCIGETVCVCRVPKSVRKANEDFRCVKCGCRGCASSD

>XP_018211566.1 hypothetical protein OGAPODRAFT_44391, partial [Ogataea polymorpha] >OBA16651.1 hypothetical protein OGAPODRAFT_44391, partial [Ogataea polymorpha]

PEEFWKLQPTLVKFEEKMKRSESKIAKMGRPKHESLWEISQINHQRSRYIYEMFYKKKLITRPTYDWLLNHKYGDAELIA

KWRKQGYENLCCLQCIEKSNANSTCICRVPKSIETQQNPACRSCGCRGCA

>XP_003866178.1 Bud31 protein [Candida orthopsilosis Co 90-125] >CCG20738.1 Bud31 protein [Candida orthopsilosis Co 90-125]

MPRTTKSKLKIKKTSPPPPGYDKIAPTILKYKQKLKSATTNSTSPTTGSTIPGAKPTSLCPIYKITHDVTKYIYDLHTRQ

KISDELYTWLTLQDYVDSLLIAKWKKQGYEKLCCTQCITGGSTCICRVPKVELLKRGQDDKVDVECVTCGCRGCASSD

>CCE41798.1 hypothetical protein CPAR2_803480 [Candida parapsilosis]

MPRSSKSAKLKIKKTSPPPQGYDKIAPTILKYKQKLKTATTNNTTNTNASSSSTTDTFPPGTKPTSLWPIYKITHDVTRY

VYDLYQRDKISNELYTWLTLQDYVDALLIAKWKKQGYEKLCCTQCIGGGAGGGTCICRVPKVELLKRGQDDKVDVECVTC

GCRGCASSD

>ONH69098.1 Pre-mRNA-splicing factor cwf14 [Cyberlindnera fabianii]

MAKIRTSRTKKAPEGFDDIKPTLESFEERLKDAQSVALHKTGRKSESLWEIFRITHERSRYVYDLHYKKKAISKDLYEWL

LKQRYADANLIAKWKKQGYENLSKGDTGKE

>XP_002549075.1 cell cycle control protein cwf14 [Candida tropicalis MYA-3404] >EER32947.1 cell cycle control protein cwf14 [Candida tropicalis MYA-3404]

MPKIKKPKKNGPPPEGYSKIEPTLTKYRDKLKTAQSKPDPTKSKQSSLWIIYELNYKISRYVYDMYKNKRISKDLYDWLL

LQNYVNSDLIAKWKKSGYEKLCCINCISTNTNGGGTCICRVPKAKLIEKDPEKVNTECITCGCRGCSSSD

>XP_723540.1 U2 snRNP complex subunit [Candida albicans SC5314] >EEQ42239.1 hypothetical protein CAWG_00443 [Candida albicans WO-1] >KGQ97972.1 bud site selection protein 31 [Candida albicans P37005] >KGR17091.1 bud site selection protein 31 [Candida albicans P78048] >KGR22621.1 bud site selection protein 31 [Candida albicans P37037] >KGT71442.1 bud site selection protein 31 [Candida albicans 12C] >KGU16560.1 bud site selection protein 31 [Candida albicans 19F] >KGU17838.1 bud site selection protein 31 [Candida albicans L26] >KGU34352.1 bud site selection protein 31 [Candida albicans P75063] >KHC60290.1 bud site selection protein 31 [Candida albicans P37039] >KHC83734.1 bud site selection protein 31 [Candida albicans SC5314] >KHC89170.1 bud site selection protein 31 [Candida albicans SC5314] >AOW26620.1 U2 snRNP complex subunit [Candida albicans SC5314]

MPKIKKPKKNGPPPEGYSKIEPTLTKYRNKLKSAQANPDPTKSKQSSLWIIYQLNYKITRYVYDTYVAKRISKELYDWLL

LQNDINKDLIAKWKKPGYEKLCCINCISTNTNGGGTCVCRVPKAKLLEKDPEKVNIECITCGCRGCASSD

>KGR14212.1 bud site selection protein 31 [Candida albicans P57072] >KHC40993.1 bud site selection protein 31 [Candida albicans P76055] >KHC41659.1 bud site selection protein 31 [Candida albicans P76067] >KHC48118.1 bud site selection protein 31 [Candida albicans Ca6] >KHC55951.1 bud site selection protein 31 [Candida albicans P60002] >KHC67599.1 bud site selection protein 31 [Candida albicans P75010]

MPKIKKPKKNGPPPEGYSKIEPTLTKYRNKLKSAQTNPDPTKSKQSSLWIIYQLNYKITRYVYDTYVAKRISKELYDWLL

LQNDINKDLIAKWKKPGYEKLCCINCISTNTNGGGTCVCRVPKAKLLEKDPEKVNIECITCGCRGCASSD

>KGQ90645.1 bud site selection protein 31 [Candida albicans P94015] >KGU13231.1 bud site selection protein 31 [Candida albicans P87] >KGU32176.1 bud site selection protein 31 [Candida albicans P34048] >KGU36774.1 bud site selection protein 31 [Candida albicans P57055] >KHC74284.1 bud site selection protein 31 [Candida albicans P75016] >KHC84386.1 bud site selection protein 31 [Candida albicans P78042]

MPKIKKPKKNGPPPEGYSKIEPTLTKYRNKLKSAQTNPDPTKSKQSSLWIIYQLNYKITRYVYDTYAAKRISKELYDWLL

LQNDINKDLIAKWKKPGYEKLCCINCISTNTNGGGTCVCRVPKAKLLEKDPEKVNIECITCGCRGCASSD

>KGR02718.1 bud site selection protein 31 [Candida albicans GC75]

MPKIKKPKKNGPPPEGYSKIEPTLTKYRNKLKSAQANPDPTKSKQSSLWIIYQLNYKITRYVYDTYAAKRISKELYDWLL

LQNDINKDLIAKWKKPGYEKLCCINCISTNTNGGGTCVCRVPKAKLLEKDPEKVNIECITCGCRGCASSD

>XP_002417574.1 bud site/cell polarity selection protein, putative [Candida dubliniensis CD36] >CAX45229.1 bud site/cell polarity selection protein, putative [Candida dubliniensis CD36]

MPKIKKPKKNGPPPEGYSKVEPTLTKYRSKLKSAQTNPDPTKSKQSSLWIIYQLNYKITRYVYDTYVAKRISKELYDWLL

LQNDINNDLIAKWKKPGYEKLCCINCISTNTNGGGTCVCRVPKAKLLEKDPEKVNIECITCGCRGCASSD

>CDK25048.1 unnamed protein product [Kuraishia capsulata CBS 1993]

MVARKPPADYVKVQPTLDAFAKKLKDLSRDDSLWEVLKINHQRSRYIYEMYYKKKLISKELYSWLGKKKVIDINLIAKWR

KQGYENLCCLKCISEKTCICRVPHQNDFKQCVNCGCKGCASSD

>OEJ85379.1 Pre-mRNA-splicing factor BUD31 [Hanseniaspora osmophila]

MKTPPPQGYEKIQKTLDSYAIELKNITTKNSSTNDXKQKLYTSSNQDFWEISRIAHKRSRYIFDLFYKRKLISKELYQWC

LDQGFADKHLIAKWKKRGYEKLCCLKCIQRVETNEGDKACICRVPRATLLKQQQQQNDKSDDGNEENPKSKNETTQSSI

LNTKIAFKQCQNCGCSGCASTD

>KTB19474.1 Pre-mRNA-splicing factor BUD31 [[Candida] glabrata]

MYYKRRAISRELYEWLLKKKVADRRLIAKWRKRGYEKLCCLQCVQQSETNHGSTCICRVPRLQLEAEAEKKGVPVSFKEC

VNCGCHGCASTD

>OEJ82450.1 Pre-mRNA-splicing factor BUD31 [Hanseniaspora opuntiae]

MIYNSAPPPEFKTFEGKLNEFDIXLKERIIEARELAKDDKQKGLREGLREDSEIFKITNEKTRYVFDLYYIEKTMSRKVF

DWILKEKLVDTLLLAKWKKKGYEQLCCVRCINRKEFLTGDKVCICRVSTNGQSKAAKKKKKVKEGEGEDNAVCTLCGCSG

CASNP

>KKA03173.1 Pre-mRNA-splicing factor HuBUD31 [Hanseniaspora uvarum DSM 2768]

MIFNSKPPEEFKNFQEKLNEFDIILKQRIIEAREKAKEDKQKGLREGLREDSEIFKINNEKTRYIYDLYYVEKTMPKKVY

QWILKEKLIDNVLLAKWKKKGYEQLCCARCINRKEFLAGDKVCICRVSTNSQNKGKKNKKKGGDGDDKEITICTLCGCNG

CTTQQ

>SGZ40370.1 related to Pre-mRNA-splicing factor BUD31 [Hanseniaspora guilliermondii]

MLYNSIPPPEFKIIEGKLNEFDIKLKERIIEAREQAKEDKQNGLREGLREDSEIFKITNEKTRYIFNLYYVEKKLPKKVF

DWIIKEKLIDTLLLAKWKKKGYEQLCCVRCINRKEFLTGDKVCICRVSTNEQNKKNKKKKQVKEAENKDSVCTLCGCSGC

TSKP

>XP_001527891.1 conserved hypothetical protein [Lodderomyces elongisporus NRRL YB-4239]
>EDK42233.1 conserved hypothetical protein [Lodderomyces elongisporus NRRL YB-4239]

MPKSTKKPKKNSLPPADFSKIAPTINQYKQKLRHAQLQPLDSKTTLSRISLTWPITRLLHNCTRFVQQQNDEGELSPELFEWLKVQD
YVDEKLLNKWGKRGYEKLCCLGCINRLGQGNGAVCVCRVPKVELLKRKNRKQLREQKGGNANGEQNDQNDQNDQGEEEDLEEESTDV
DKVDVECVTCGCRGCASTD

Taphrinomycotina

>NP_595965.1 G10 protein [Schizosaccharomyces pombe 972h-]

MPRLRTSRTKRPPDGFDEIEPTLIEFQDRMRQIENTMGKGTKTEMLAPIFQLHHQRSRYIYDLYYKREAISTELYNWLLKQNYADGN
LIAKWKKPGYEKLCCLRCIQTAESKFGSTCICRVPKSKLDKDQRVRCTHCGCNGCASCD

>XP_013018179.1 G10 protein [Schizosaccharomyces octosporus yFS286]

MPRIRTSRTKRPPEGFEEIEPTLLEFQERMRQVENTTGKGSKSESLAPIFQLHHQRSRYVYDLYYKREAISSELYNWLLKQNYADAN
LIAKWKKPGYEKLCCLRCIQTAESKFGSTCICRVPKAKLGKDAPSTCVHCGCRGCASGD

>XP_013022759.1 G10 protein [Schizosaccharomyces cryophilus OY26]

MPRIRTSRTKRPPEGFEEIEPTLLEFQERMREAENTTGKGSKSESLAPIFQLHHQRSRYVYDLYYKREAISTELYNWLLKQNYADAN
LIAKWKKAGYEKLCCLRCIQTTESKFGSTCICRVPKAKLGKDAPSTCVHCGCRGCASGD

>XP_002173438.1 G10 protein [Schizosaccharomyces japonicus yFS275]

MPRIRTSRSKRPPEGFDAIEPTLLEFQDMMRQAENTPSKKTKSEALAPIFRIHHQRSRYVYDLYYKREAISTELYEWLLKQNYADAN
LIAKWKKPGYEKLCCLKCIQTSESKFGSTCICRVPKAKLGKDTHVECVHCGCHGCASGS

>XP_007872522.1 pre-mRNA-splicing factor cwf14 [Pneumocystis murina B123]

MPRIRTLRAKPPPEGFDDIEPTLHDFAQKMKDIENEPHENKSRQGALAPIFQIHHQRSRYIYDLYYKREAISKELYEWLLKQGYADG
NLIAKWKKNGYEKLCCLRCIQTKEMNFNSTCICRVPKIHLPKATIVECVHCGCRGCASCD

>XP_018230437.1 pre-mRNA-splicing factor cwf14 [Pneumocystis jirovecii RU7]

MPRIRTLRAKPPPEGFDDIEPILHDFAQKMKDIENEPYENKSRQGALAPIFQIHHQRSRYIYDLYYKREAISKELYEWLLKQGYADG
NLIAKWKKNGYEKLCCLRCIQTKEMNFNSTCICRVPKAHLPKATIVECVHCGCRGCASCD

>XP_018225425.1 pre-mRNA-splicing factor cwf14 [Pneumocystis carinii B80]

MPRIRTLRAKLPPEGFDDIEPILHDFAQKMKDIENEPHENKSRQGALAPIFQIHHQRSRYIYDLYYKREAISKELYDWLLKQGYADG
NLIAKWKKNGYEKLCCLRCIQTKEMNFNSTCICRVPKIHLPKATIVECVHCGCRGCASCD

>XP_019025952.1 bud site selection-related protein [Saitoella complicata NRRL Y-17804]

MPRIKTQRTKKAPEGFDEIESTLLDFAQKMKDAENVSHEGKRKNEALWAIFQISHQRSRYIYELYYKREAISRELYDWLLKQNYADA
NLIAKWKKQGYEKLCCLRCIQMKEQNFGTTCICRVPKAKLSKGAPVECLHCGCRGCASSD

>ORY78233.1 cell cycle control protein cwf14 [Protomyces lactucaedebilis]

MPRIRTNRAKRPPAGFEDVEPMLEDFAQKMKDVENAPHENKSKTGTLAPIFQIHHQRSRYIYDLYYKRETISKELYEYLLKQGYGDG
NLIAKWKKPGYEKLCCLRCIQTKETNFNTTCICRVPKVKLGEGELIECHQCGCRGCGSSD

>OLL22976.1 Pre-mRNA-splicing factor cwf14 [Neolecta irregularis DAH-3]

MPKVRTLRTKKPPPGYAEIEATLLEFSRKMKDAENTPHEGLSKRQSLWPIFQISHQRSRYIYELFYKRESISEDLYQWLLKQGYADG
KLIAKWKKNGYEKLCCLQCIQSKETNFNGTCICRVPRAKVDTDKNVECITCGCRGCCSGD
